# MOF/polymer hybrids through *in situ* free radical polymerization in metal-organic frameworks†

**DOI:** 10.1039/d2mh01202b

**Published:** 2023-01-19

**Authors:** Marzena Pander, Rodrigo Gil-San-Millan, Pedro Delgado, Cristina Perona-Bermejo, Urszula Kostrzewa, Karol Kaczkowski, Dominik J. Kubicki, Jorge A. R. Navarro, Wojciech Bury

**Affiliations:** a Faculty of Chemistry, University of Wrocław 14 F. Joliot-Curie 50-383 Wrocław Poland wojciech.bury@uwr.edu.pl; b Departamento de Química Inorgánica, Universidad de Granada, Av. Fuentenueva S/N 18071 Granada Spain jarn@ugr.es; c Department of Physics, University of Warwick Coventry CV4 7AL UK dominik.kubicki@warwick.ac.uk

## Abstract

We use the free radical polymerization initiator 4,4′-azobis(cyanovaleric acid) coordinated to the open metal sites of metal-organic frameworks (MOFs) to give rise to highly uniform MOF/polymer hybrids. We demonstrate this strategy on two robust zirconium MOFs (NU-1000 and MOF-808), which are the most effective catalysts for degradation of chemical warfare nerve agents. The resulting hybrid materials maintain their hydrolytic catalytic activity and have substantially improved adhesion to polypropylene and activated carbon textile fibers, yielding highly robust MOF/polymer/textile hybrid systems. These composites are suitable for the green production of active protective clothing and filters capable of detoxifying organophosphorus warfare agents.

## Abstract 2

A simple yet powerful strategy of carrying out free radical polymerization of methacrylate monomers using a MOF-based initiator. The obtained composites maintain high catalytic activity towards a chemical warfare agent (CWA) simulant.

## Graphical abstract

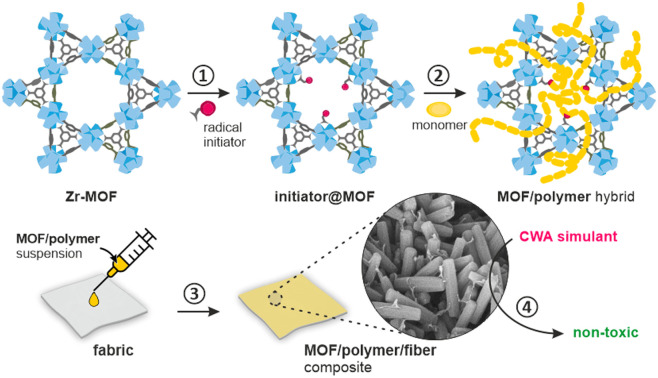

**New concepts**

Efficient integration of Metal-Organic Frameworks (MOF) and organic polymers into hybrid materials is of fundamental importance for enabling broad industrial use of MOFs, because of their superior elasticity, processability and ease of deposition. The MOF/polymer composites with fibers (or textiles) can significantly improve the technological processes of applications in filtration, chemical warfare or antimicrobial protection, catalysis, energy storage, sensors, and drug delivery. However, the existing synthetic approaches have substantial drawbacks, such as heterogeneity, which creates weak points in the composites, and in some cases require complex chemistry that does not easily carry over to other MOFs and polymers. A fundamentally different approach to synthesizing these materials is therefore necessary. In our work, we overcome these limitations and introduce a new synthetic protocol, which enables precise control of the MOF-polymer interaction and can be applied to a variety of MOFs and polymers with different chemistries. The resulting MOF/polymer hybrids exhibit substantially enhanced processability as demonstrated by improved adhesion to selected textile fibers and show that the MOF–polymer hybrids have high activity for decomposition of a model nerve agent.

## Introduction

The principles of reticular chemistry developed over the past two decades have brought about an unprecedented variety of new periodic structures that can be designed and synthesized in a controlled fashion.^1^ The most prominent example of this are metal-organic frameworks (MOFs), a class of porous coordination polymers, with unique and programmable properties.^2,3^ Over the last decade, zirconium-based MOFs (Zr-MOFs) have attracted particular attention because of their high thermal and chemical stability.^4^ Zr-MOFs have been shown to excel in sorption and separation processes,^5^ and have been used in biomedical applications,^6,7^ heterogeneous catalysis,^8^ and electronic devices,^9^ among others. These materials can also be readily modified after synthesis, which substantially broadens the scope of their applications.^10^

**Scheme 1:**
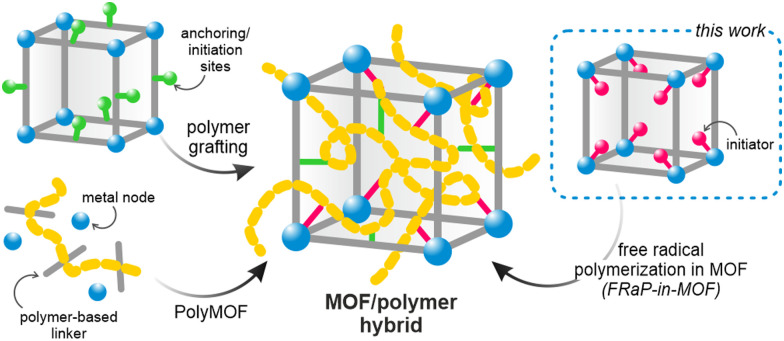
Synthetic strategies for the preparation of MOF/polymer hybrids.

However, the key roadblock on the way to their wider industrial use is their insoluble crystalline powder form, which makes them exceptionally difficult to process.^11^ Two main approaches to addressing this shortcoming have been previously explored: amorphization into liquid or glassy states,^12,13^ and combining MOFs with organic polymers.^14–16^ The latter strategy is particularly appealing as it, in principle, provides a large parameter space and could be highly tunable. The previously reported strategies yielding MOF/polymer composites rely on mixing the two components^14,17^ or carrying out the polymerization in the presence of the MOF powder.^18–20^ However, these approaches lead to heterogeneous composites with a non-uniform distribution of the two components, and sometimes result in complete blockage of the MOF pores.^21^ To overcome these limitations, MOF/polymer hybrids were proposed, whereby the framework and polymer are covalently bound (Scheme 1).^22^ In this approach, the polymer loading level and its location within the MOF structure can be controlled by choosing specific anchoring sites.^14^ Other hybridization strategies include *in situ* synthesis of oligo- or polyMOFs^23–27^ and covalent modification of MOFs’ surface by atom transfer radical polymerization (ATRP).^28–31^ However, these methods require laborious multi-step synthesis of the MOF linkers and cannot be easily scaled up or adapted to new systems. We realized that a more universal and straightforward approach is necessary to enable the exploration of vast compositional space of conceivable MOF/polymer hybrids. This would also enable combining polymers and MOFs with well-established functional properties to yield easily processable and ready-to-use formulations.

**Fig. 1:**
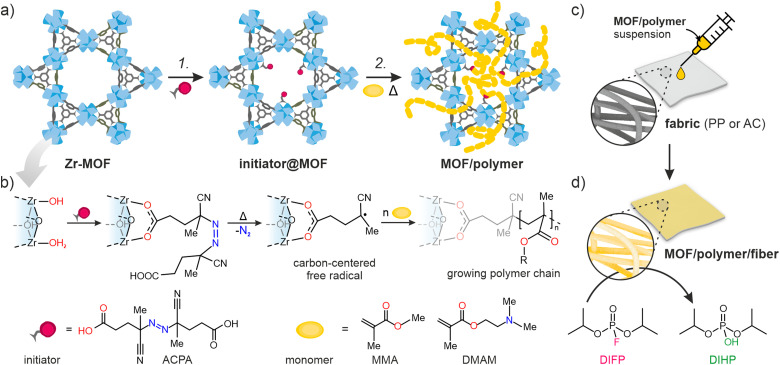
The two-step protocol for synthesizing MOF/polymer hybrids and their composites with fibers. (a) Reaction scheme. Step 1: Solvent-assisted ligand incorporation (SALI) of ACPA (initiator) into a Zr-MOF; step 2: free-radical polymerization in a MOF (*FRaP-in-MOF*) of acrylate monomers, (b) SALI of 6- (MOF-808) or 8-connected (NU-1000) Zr-nodes with ACPA followed by temperature-induced generation of carbon-centered radicals, (c) drop-casting of MOF/polymer hybrids on PP and AC fibers, (d) hydrolysis of a nerve agent simulant, diisopropyl fluorophosphate (DIFP), catalyzed by the MOF/polymer/fiber composite (conditions: RT, 24 h).

Here, we introduce the novel Free-Radical Polymerization in a MOF (*FRaP-in-MOF*) strategy for the preparation of MOF/polymer hybrids with high affinity for non-covalent attachment to functional textile fibers (Fig. 1). We take advantage of a single component MOF-based initiator, which is active in *FRaP-in-MOF* of vinyl monomers (Scheme 1 and Fig. 1a). We focus on mesoporous Zr-MOFs, such as MOF-808^32,33^ and NU-1000,^34^ that are highly efficient catalysts for the hydrolytic degradation of extremely toxic organophosphorous chemical warfare agents (CWAs), such as sarin or soman.^35–37^ In the first step, the azo-initiator, 4,4′-azobis(cyanovaleric acid) (ACPA), was post-synthetically attached to the Zr_6_-nodes in MOF-808 and NU-1000 through solvent-assisted ligand incorporation (SALI).^38,39^ In the second step, the initiator@MOF materials were used to initiate *FRaP-in-MOF* of methyl methacrylate (MMA) and 2-dimethylaminoethyl methacrylate (DMAM). We show that MOF/polymer hybrids can be easily deposited on polypropylene (PP) or activated carbon (AC) fabrics by drop-casting to form three-component MOF/polymer/fiber composites with excellent integrity (Fig. 1c) and catalytic activity for the degradation of a model toxic agent simulant, diisopropyl fluorophosphate (DIFP) (Fig. 1d).

## Results and discussion

### Functionalization of Zr-MOFs with the 4,4′-azobis(cyanovaleric acid) radical initiator and its thermal stability

**Fig. 2:**
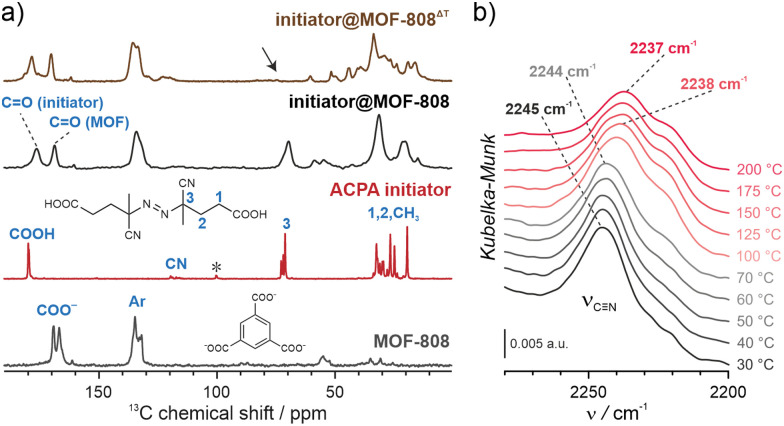
Thermal decomposition of the ACPA initiator. (A) ^13^C CP-MAS spectra of MOF-808, pristine ACPA, and initiator@MOF-808 before and after thermal activation; the arrow indicates a signal sensitive to the initiator thermal activation. (B) VT-DRIFT spectra of initiator@MOF-808 (the C≡N vibration range) measured in the temperature range of 30–200 °C.

**Fig. 3:**
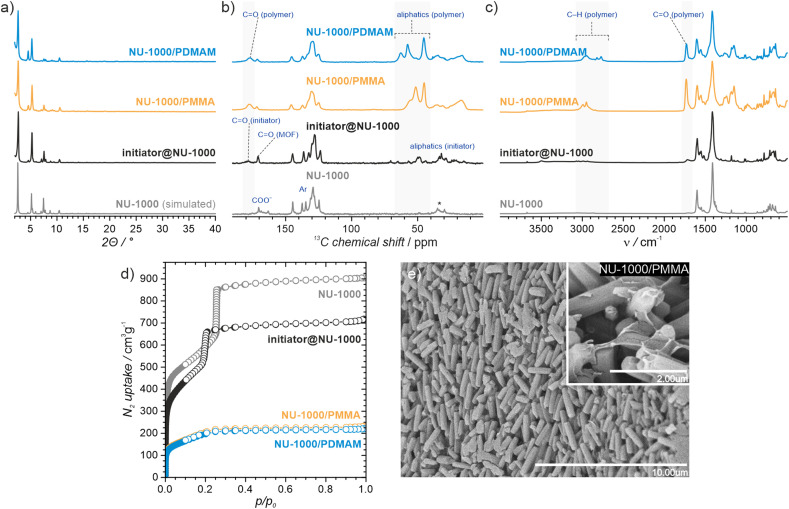
Structural and morphological characterization of the initiator@NU-1000, NU-1000/polymer and pristine NU-1000 materials. (a) PXRD analysis; (b) ^13^C CP-MAS spectra; (c) DRIFT spectra; (d) N_2_ sorption isotherms at 77 K (filled and open circles denote adsorption and desorption, respectively); (e) SEM image of the NU-1000/PMMA hybrid.

NU-1000 and MOF-808 were prepared following the literature protocols (see details in the ESI†).^40,41^ In terms of node connectivity, which describes the number of carboxylate groups (belonging to linkers) bound to one metal node, NU-1000 and MOF-808 contain 8- and 6-connected Zr_6_-nodes, respectively (Fig. S1, ESI†). The remaining coordination sites on the nodes (4 for **NU-1000** and 6 for **MOF-808**) can be completed post-synthetically with other non-structural carboxylate ligands using SALI.^38^ Here, we employed 4,4′azobis(cyanovaleric acid) (ACPA) which contains two carboxylic groups capable of coordinating to the Zr_6_ -nodes, in addition to being a common radical initiator that undergoes homolytic bond cleavage and forms free radicals when exposed to elevated temperatures or UV light (Fig. 1b).^42^ To avoid accidental ACPA decomposition during SALI, all manipulations were carried out at room temperature. The amount of ACPA in the resulting initiator@MOF was calculated based on the ^1^H NMR spectra of samples dissolved in a D_2_SO_4_/DMSO-d_6_ solution (Fig. S10 and S11, ESI†). For NU-1000 and MOF-808 we observed approximately 2.5 and 3.0 ACPA molecules per Zr_6_-node, respectively. The PXRD analysis (Fig. 3a and Fig. S3, ESI†) and SEM images (Fig. S38 and S39, ESI†) showed that the crystallinity and microcrystal morphology of MOFs after SALI were unaffected. The presence of ACPA in the initiator@MOF materials was corroborated by solid-state NMR (Fig. 2a and Fig. S16, ESI†) and diffuse reflectance infrared Fourier transform (DRIFT) studies (Fig. S19 and S20, ESI†).

To assess the porosity of initiator@MOF, we performed N_2_ sorption measurements at 77 K (Fig. 3d and Fig. S27, ESI†). The incorporation of ACPA into the MOF leads to a slight decrease of the total pore volume and the Brunauer–Emmett–Teller (BET) surface area (Table S2, ESI†). The total pore volumes were 0.36 cm^3^ g^−1^ for initiator@MOF-808, 0.81 cm^3^ g^−1^ for pristine MOF-808, 1.10 cm^3^ g^−1^ for initiator@NU-1000, and 1.39 cm^3^ g^−1^ for pristine NU-1000. A similar trend was observed for BET surface areas: 794 m^2^ g^−1^ for initiator@MOF-808, 1127 m^2^ g^−1^ for pristine MOF-808, 1838 m^2^ g^−1^ for initiator@NU-1000, and 2052 m^2^ g^−1^ for NU-1000. Pore size distributions (PSD) calculated using Density Functional Theory (DFT) (Fig. S28, ESI†) showed that the ACPA moieties mostly occupy the large cavities in MOF-808, and mesoporous channels in NU-1000 (Fig. S1, ESI†). To confirm organic monomer pore accessibility, we exposed the initiator@MOFs to MMA and DMAM vapors at room temperature for 24 h. Afterwards, we calculated the monomer loadings based on ^1^H NMR spectra of samples dissolved in D_2_SO_4_/DMSO-d_6_ solution (Fig. S12–S15, ESI†). For initiator@NU1000, we observed about 27 MMA and 7 DMAM molecules per Zr_6_-node, while for initiator@MOF-808, 14 MMA and 3 DMAM molecules per Zr_6_-node. These results clearly indicate that the porosity of initiator@MOF is accessible for the organic monomers, which can be further polymerized inside the MOF.

We next confirmed that thermal activation of initiator@MOF leads to bond cleavage in the ACPA moiety. While the lifetime of the radicals is too short to permit detection by CW EPR, we carried out liquid- and solid-state NMR, and infrared spectroscopy, which unambiguously confirmed the structural change of ACPA. First, the solid initiator@MOF samples were heated at 70 °C for 24 h to allow thermal decomposition of ACPA (the resulting material is denoted initiator@MOF^ΔT^). Next, the initiator@MOF^ΔT^ samples were dissolved in a D_2_SO_4_/DMSO-d_6_ solution and ^1^H NMR spectra were collected that showed a new set of characteristic signals in the aliphatic region (Fig. S10 and S11, ESI†). We compared these results with NMR spectra of pure ACPA before and after thermal treatment under similar conditions (Fig. S9, ESI†), and assigned these signals to the radical recombination and decomposition products of ACPA.^42^ The ^13^C CP MAS NMR spectra of the solid initiator@MOF-808 before and after heat treatment reveal that the signal at 70 ppm, corresponding to the quaternary C–N=N carbon, disappears after high temperature is applied (Fig. 2a). This occurs because the loss of the electron withdrawing N=N bridge leads to increased shielding of the quaternary carbon shifting its signal down in frequency to the crowded aliphatic region. Finally, we recorded variable temperature-DRIFT (VT-DRIFT) spectra on initiator@MOF during heating from room temperature to 200 °C with a ramp rate of 10 °C min^−1^ (Fig. 2b and Fig. S21, S22, ESI†). They show a gradual shift of the C≡N vibration band from 2245 cm^−1^ to 2237 cm^−1^, corroborating the temperature-triggered irreversible ACPA transformation.

### Radical polymerization of selected monomers initiated by initiator@MOF materials.

Having confirmed the successful incorporation of the initiator into MOF-808 and NU-1000, we proceeded to test the ability of the resulting materials to promote *FraP-in-MOF* using two vinyl monomers, MMA and DMAM (Fig. 1a), which differ in size, reactivity, and functionality. All polymerization reactions were carried out in pure monomers under dinitrogen. To exclude the possibility that the unmodified MOFs can induce polymerization, we performed control experiments, which showed that they are, indeed, inactive. These experiments also excluded the possibility of spontaneous self-polymerization of the neat monomers (see Section S14, ESI†). The *FRaP-in-MOF* reactions were carried out at 70 °C (for MMA) and at 50 °C (for DMAM) for 48 hours using 100 mg of initiator@MOF for every batch. After each *FRaP-in-MOF* reaction, we performed extensive washing with methanol and acetone to remove unreacted monomer and excess polymer that was not strongly attached to the MOF (for details see the ESI†). The formation of separated polymer chains, that were not attached to the MOF, may be caused by a spontaneous transfer of radical species during polymerization. We found that *FRaP-in-MOF* followed by washing consistently yielded reproducible results, as shown by tests repeated on several batches of NU-1000/PMMA (see Section S13, ESI†). To quantify the composition of the MOF/polymer hybrids, we used TGA and ICP-OES (see section S12 for details, ESI†). In all the MOF/polymer samples, we observed a similar polymer content of approximately 30 wt%. Based on these values, we estimated the number of polymerized monomers per Zr_6_-node in the final MOF/polymer hybrids (Table S5, ESI†). We observed similar trend of polymer loadings of 15 MMA (and 13 DMAM) molecules/node in NU-1000/polymer, and 4 MMA (and 5 DMAM) molecules/node in MOF-808/polymer hybrids. Matrix assisted laser desorption and ionisation with mass spectrometry (MALDI-MS) analysis of digested sample of NU-1000/PMMA (Fig. S72, ESI†) confirmed the presence of polymer chains with a length up to 20 mers. We also noticed the increased number of DMAM molecules/node, as compared to the adsorption experiment, which we attributed to the better polymerization efficiency in liquid phase of this monomer.

We confirmed the presence of polymers in MOF/polymer hybrids with infrared and solid-state NMR spectroscopy, and SEM. Fig. 3c and Fig. S23–S26 (ESI†) show the DRIFTS spectra of MOF/polymer samples. In all MOF/polymer materials, we observed the presence of characteristic vibrations of carbonyl C=O and aliphatic C–H bonds at 1730 cm^−1^ and below 3000 cm^−1^, respectively. Fig. 3b shows the ^13^C CP MAS NMR spectra of the MOF/polymer materials. While pristine NU-1000 is characterized by relatively narrow peaks, the peaks broaden when the initiator is introduced and then broaden further when the polymers are formed. This result shows that each of these synthetic steps decreases conformational freedom of both the host framework and guest molecules corroborating that the polymers are embedded within the MOF and in direct atomic-level contact with the linkers. The SEM images of MOF/polymer hybrids show some characteristic features and significant differences in the arrangement of crystallites as compared to the pristine MOFs (see Section S10.2, ESI†). For example, in NU-1000/PMMA the PMMA layer wraps around the microcrystals of NU-1000 (Fig. 3e). In addition, the arrangement of microcrystals in NU-1000/PMMA is more uniform, as compared to pristine NU-1000, owing to the improved adhesion between microcrystals induced by the polymer phase, and the lack of larger MOF crystals (diameter > 3 μm) due to the washing procedure (Fig. S36b and S38, ESI†).

**Fig. 4:**
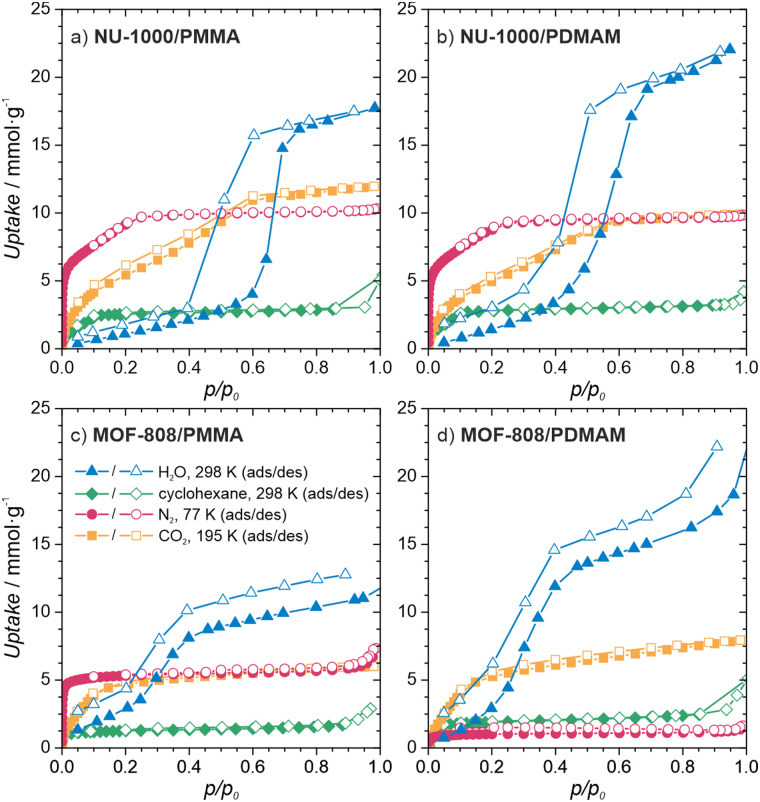
Characterization of the MOF/polymer hybrids porosity. Sorption isotherms of (a) NU-1000/PMMA, (b) NU-1000/PDMAM, (c) MOF-808/PMMA, (d) MOF-808/PDMAM.

To gain insight into the properties of the MOF/polymer hybrids in the context of their catalytic potential, we performed sorption studies using N_2_ (at 77 K), CO_2_ (at 195 K), cyclohexane (at 298 K), and H_2_O (at 298 K). The adsorption–desorption isotherms are shown in Fig. 3d and 4. The N_2_ sorption isotherms show good porosity for all MOF/polymer hybrids, except MOF-808/PDMAM. The CO_2_ isotherms, however, confirm significant porosity of all materials. It is thus noteworthy that MOF-808/PDMAM is porous towards CO_2_ but not N_2_ and cyclohexane. We suggest this is likely owing to the smaller kinetic diameter of CO_2_ than N_2_ and cyclohexane molecules allowing the former to diffuse in its constricted pore structure. The significant decrease in pore volume for MOF/polymer materials confirms that the polymer chains are located in the MOF pores, in agreement with the solid-state NMR data (see Table S2 and Fig. S29, S31, ESI†). Moreover, all NU-1000/polymer hybrids showed higher uptake of all the adsorbates as compared to MOF-808/polymer materials, which agrees with the difference in porosity of the corresponding pristine MOFs. The adsorption of H_2_O vapors shows small differences in total water uptake for all the materials (10–22 mmol g^−1^); however, we observed that the adsorption/desorption branches for NU-1000/PDMAM are shifted to lower *p/p*_0_ indicative of higher water affinity than NU-1000/PMMA (see Table S2, ESI†). The same relation is true for MOF-808/PDMAM and MOF-808/PMMA, with the former being more hydrophilic and showing two times higher water uptake than the latter. This effect may be explained in terms of higher hydrophilicity of PDMAM compared to PMMA, because the former contains residual amino groups.

### Preparation and water stability of 3-component MOF/polymer/fiber composites.

To date, MOF crystals with CWA detoxification activity have been deposited or incorporated *in situ* into various types of fibers, including cotton,^43–46^ poly(vinylidene)fluoride (PVDF),^47,48^ poly(ethylene terephthalate) (PET),^49^ polypropylene,^50^ activated carbon (AC),^51^ and a variety of TiO_2_-modified polymers.^52–55^ Those works have shown that covalent binding between the MOF and polymer benefits the integrity of the MOF/polymer/fabric composite,^56^ and its catalytic properties.^57^ However, the previously mentioned approaches give rise to either non-homogeneous composites, poor attachment to fiber and/or are not easy to transfer to industrial processes. The incorporation of polymer chains into the MOF structure substantially enhances their applicability across the board. For example, the polymer may enable binding to surfaces such as fabrics, beads, or membranes.^58^

**Fig. 5:**
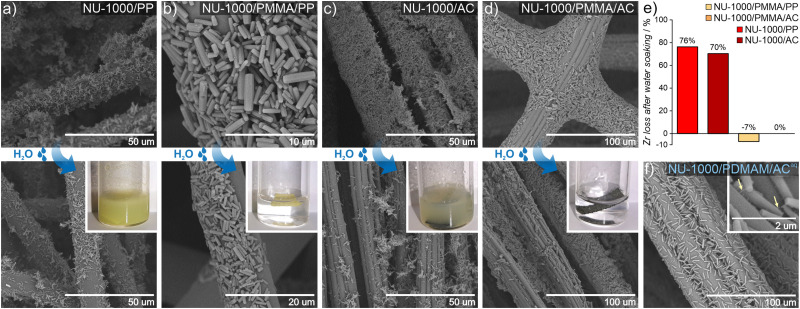
Impact of *FRaP-in-MOF* on MOF crystals attachment to textile fibers. SEM images of the MOF/fiber and MOF/polymer/fiber composites before (top) and after (bottom) water treatment: (A) NU-1000/PP, (B) NU-1000/PMMA/PP, (C) NU-1000/AC and (D) NU-1000/PDMAM/AC; (E) Zr loss (in %) after soaking the composites in water (based on ICP-OES, estimated error is ±10%); (F) SEM image of the NU-1000/PDMAM/AC^aq^ composite prepared using the aqueous dispersion of NU-1000/PDMAM. The arrows indicate the polymer phase involved in composite adhesion.

We explore this idea here by preparing MOF/polymers integrated into polypropylene (PP) and activated carbon (AC) fibers to enable their use in protective suits. We chose these two fibers because of their substantially different physical properties: AC possesses a hydrophobic porous network and is extremely heat resistant, while PP is highly impermeable, non-porous and melts easily. MOF/polymer/fiber composites were synthesized by impregnating fabrics with MOF/polymer suspensions in THF (see the ESI† for details). We also prepared control samples using pristine NU-1000 and MOF-808 without polymers. SEM images show that for the 3-component composites, homogeneous integration with uniform coverage and good MOF-fiber and MOF-MOF adhesion was achieved (Fig. 5c and d, see Section S10.3, ESI†). On the other hand, for pristine MOFs, we observed a non-uniform distribution of MOF microcrystals within the composite.

An additional requirement for protective military suits is that they should be highly water-resistant, and this adds the challenge of preventing MOF leaching during washing.^59^ Therefore, to assess the stability and strength of adhesion between the MOF/polymer hybrids and the selected fibers, we designed and performed a water stability test. We suspended pieces of MOF/polymer/fiber and MOF/fiber composites in water at room temperature, without stirring (see ESI† for further details) and carried out three soaking cycles. We then compared the MOF content in the dried composites before and after water soaking using ICP-OES analysis. For all MOF/polymer/fiber materials, we observed remarkable stability in water without substantial MOF leaching, while for the MOF/fiber samples, immediate leaching of the MOF powder was evident by visual inspection, and confirmed by the ICP-OES measurements, with a MOF loss of 76% and 70% for NU-1000/PP and NU-1000/AC, respectively. (Fig. 5e). This experiment demonstrates the substantially improved attachment of MOF/polymers to fibers with various physical characteristics, which enhances their stability in water. We attribute this improved stability to crystal surface polarity modification after the polymerization process with the concomitant higher compatibility to the PP and AC fiber surfaces.

We also tested the possibility to prepare uniform dispersions of MOF/polymer materials in water, because green, water-based fabric impregnation may be of industrial relevance. NU-1000/PDMAM could be uniformly dispersed in water, and the obtained mixture was afterwards used for coating of hydrophobic AC fibers. As a result, a very uniform distribution of NU-1000/PDMAM microcrystals was obtained, leading to MOF/PDMAM/AC^aq^ composite (Fig. 5f).

### Catalytic activity of MOF/polymer and MOF/polymer/fiber materials in DIFP degradation.

**Fig. 6:**
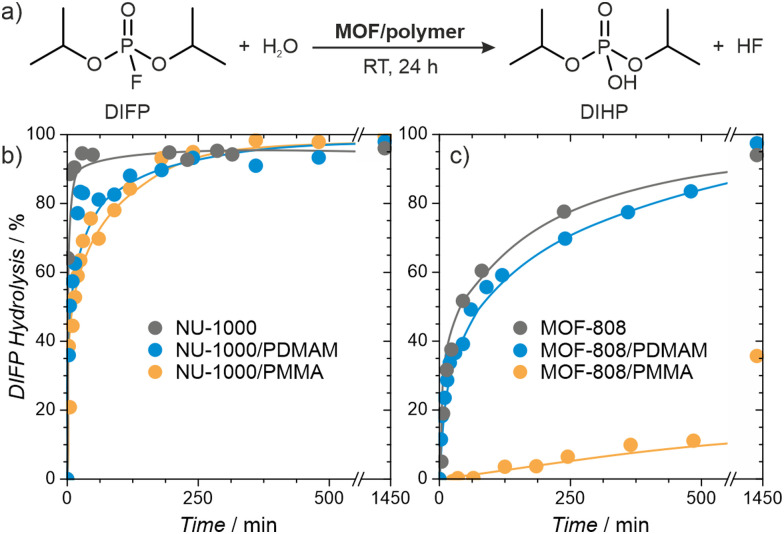
Hydrolysis of the nerve agent simulant (DIFP) catalysed by MOF/polymer hybrids. (a) Reaction scheme; and reaction kinetics for (b) NU-1000/polymer and (c) MOF-808/polymer hybrids. The reactions were carried out at room temperature for 24 hours, using DIFP:MOF/polymer ratio of 2 : 1 in bufferless solutions.

Organophosphorus and organosulfur chemicals, termed nerve and vesicant agents, respectively, are subclasses of CWAs of special concern due to their acute toxicity and availability.^60–62^ Diisopropylfluorophosphate (DIFP) contains the P–F bond present in some G-type nerve agents but is significantly less toxic, and we use it here as a nerve agent simulant. The pristine MOFs are highly active catalysts, but the presence of a polymer within the pores could conceivably reduce their activity. We followed the catalytic activity of the MOF/polymer hybrids in the hydrolysis of DIFP to nontoxic diisopropylphosphate (DIHP), at room temperature, in bufferless solutions, over 24 hours and compared them with those of pristine MOF-808 and NU-1000 (Fig. 6). Remarkably, the NU-1000/polymer composites maintained high activity, similar to pristine NU-1000, despite their nearly four-fold lower porosity. The performance of MOF-808/PMMA was significantly worse, resulting in only 36% of DIFP being decomposed after 24 hours. On the other hand, the less porous MOF-808/PDMAM achieved a DIFP conversion of 97% after 24 h, matching the activity of the pristine MOF-808. We attributed this remarkable improvement to the presence of the amino functional groups of PDMAM which increased the local basicity/nucleophilicity of the catalyst (Table S9, ESI†).^63^

Having confirmed the high activity of MOF/polymer hybrids, we carried self-cleaning tests for the MOF/polymer/fiber composites. Depending on the MOF/polymer content attached to the AC or PP fibers, different ratios of catalyst were used, namely 15 and 5-molar excess of DIFP for NU-1000 and MOF-808 based composites, respectively. The evolution of the detoxification reaction was evaluated using a spike of 2.0 μL of water and 0.3 μL of DIFP at 30 °C (MOF/polymer/PP) or 60 °C (MOF/polymer/AC) followed by adsorbate phase extraction after 24 h with 2 mL of CH_2_Cl_2_. The results show 66% and 97% of DIFP being hydrolysed for NU1000/PDMAM/PP and MOF-808/PDMAM/PP, respectively. In the case of (MOF/polymer/AC) composites 0% and 42% of DIFP are hydrolysed for NU1000/PDMAM/AC and MOF-808/PDMAM/AC, respectively. The poorer performance of the MOF/polymer/AC is a consequence of the competitive DIFP adsorption on the hydrophobic activated carbon pore structure. These results confirm that MOF/polymer hybrids are a viable way for creating fabric composites that can be applied in protective clothing against CWAs.

## Conclusions

In summary, we developed a versatile strategy for making MOF/polymer hybrids using *in situ* free radical polymerization inside the MOF (*FRaP-in-MOF*) by taking advantage of the uniform distribution of the initiating centers throughout the MOF and its high porosity for vinyl monomers. Owing to the direct coordination of the initiator to the inorganic nodes of the MOF, we obtained MOF/polymer materials with improved and uniform dispersion of both components. The in-grown polymers enhance the stability and processability of the MOF/polymer hybrids, with improved adhesion to fibers and fabrics, and introduce the potential for fine-tuning of their properties, *e.g.*, pH or hydrophilicity. We demonstrated that MOF/polymer/fiber composites are stable in water and active in decomposition of a model nerve agent simulant. Currently, the accessible initiator@MOF architectures are limited to the carboxylate-based MOF structures with lower node connectivity which can undergo SALI modification. However, this methodology can be readily extended to other types of monomers undergoing free radical polymerization offering MOF/polymer hybrids of desired complexity.

## Data availability

All data used in this work is available from the corresponding authors upon request.

## Author contributions

M. P. – investigation, data analysis, visualization, funding acquisition, writing – original draft; R. G. S. M. – investigation, data analysis, writing – original draft; P. D., C. P. B., U. K., K. K., – investigation, data analysis; D. J. K – investigation, data analysis, visualization, resources, writing – review & editing; W. B., J. A. R. N. – conceptualization, supervision, resources, funding acquisition, writing – review & editing.

## Conflicts of interest

There are no conflicts to declare.

## Supplementary Material

MH-010-D2MH01202B-s001
