# Supplementary material for: MOF/polymer hybrids through in situ free radical polymerization in metal-organic frameworks
Source: Mater Horiz. 2023 Jan 19;10(4):1301–8. doi: 10.1039/d2mh01202b (PMC10068906; doi:10.1039/d2mh01202b)
Supplement: MH-010-D2MH01202B-s001 [file MH-010-D2MH01202B-s001.pdf]

Electronic Supplementary Material (ESI) for Materials Horizons.
This journal is © The Royal Society of Chemistry 2022

Supporting Information for

MOF/polymer hybrids through *in situ* free radical polymerization in Metal-Organic Framework

Marzena Pander,[a] Rodrigo Gil-San-Millan,[a] Pedro Delgado,[b] Cristina Perona-Bermejo,[b] Urszula Kostrzewa,[a] Karol Kaczkowski,[a] Dominik J. Kubicki,[c,*] Jorge A. R. Navarro,[b,*] and Wojciech Bury[a,*]

[a]*Faculty of Chemistry, University of Wrocław, 14 F. Joliot-Curie, 50-383 Wrocław, Poland*

[b]*Departamento de Química Inorgánica, Universidad de Granada, Av. Fuentenueva S/N, 18071 Granada, Spain*

[c]*Department of Physics, University of Warwick, Coventry CV4 7AL, United Kingdom*

*Corresponding authors: wojciech.bury@uwr.edu.pl, jarn@ugr.es, dominik.kubicki@warwick.ac.uk

Table of Contents

S1. Materials 3

S2. Instrumentation 3

S3. Experimental procedures 4

S3.1. Synthesis of MOF materials 4

S3.2. Preparation of initiator@MOF via SALI reaction 5

S3.3. Preparation of MOF/polymer hybrids 5

S3.4. Fabrics coating procedure 5

S4. PXRD analysis 6

S5. NMR analyses in solution 8

S5.1. NMR spectra of ACPA and its stability studies 8

S5.2. ^{1}H NMR spectra of initiator@MOF samples 10

S5.3. ^{1}H NMR spectra of monomers loaded in initiator@MOF samples 12

S6. Solid-state ^{13}C CP MAS NMR spectra of the materials 13

S7. DRIFT spectra 14

S7.1. DRIFT spectra of the ACPA initiator and its thermal stability studies 14

S7.2. DRIFT spectra of initiator@MOF samples 15

S7.3. DRIFT spectra of MOF/polymer samples 18

S8. Sorption studies 22

S8.1. N_2 sorption isotherms 22

S8.2. Carbon dioxide sorption isotherms 26

S8.3. Cyclohexane and water vapours sorption isotherms 27

S10. SEM images and EDS analysis 28

S10.1. SEM images of initiator@MOF samples 28

S10.2. SEM images of MOF/polymer materials 29

S10.3. SEM images of MOF/polymer/fiber composites 31

S11. TGA-DTG analysis 38

S12. Composition of MOF/polymer hybrids from TGA and ICP-OES 40

S13. Reproducibility tests of the *FRaP-in-MOF* protocol 41

S14. Preparation of MOF/polymer hybrids - control experiments 43

S15. Stability of the MOF/polymer/fiber composites 46

S16. Catalytic studies with diisopropylfluorophosphate (DIFP) 48

S16.1. Reaction of DIFP with MOF/polymer hybrids 48

S16.2. Reaction of DIFP with MOF/polymer/fiber composites 49

S17. MALDI-MS spectra of MOF/polymer hybrids 51

S18. References 52

S1. Materials

Zirconium oxide dichloride octahydrate ($ZrOCl_2 \times 8H_2O$, Alfa Aesar, 98%), 1,3,5-benzenetricarboxylic acid (BTC, TCI, 98%), benzoic acid (Acros, 99%), trifluoroacetic acid (TCI, 99%), formic acid (Chempur, 85%), hydrochloric acid (Stanlab Sp. J., 35-38%), 4,4'-azobis(4-cyanovaleric acid) (ACPA, Sigma-Aldrich, 98%), methyl methacrylate (MMA, Alfa Aesar, 99%, stab.), 2-(Dimethylamino)ethyl methacrylate (DMAM, Alfa Aesar, 97%, stab. with ca 0.2% 4-methoxyphenol). 1,3,6,8-tetrakis(p-benzoic acid)pyrene (H_4TBAPy) was synthesized as described in literature.[1]

Solvents: *N,N*-dimethylformamide (Karpinex, analytical grade), ethanol (J.T.Baker, 99.5%), dichloromethane (VWR, analytical grade), acetonitrile (POCH, 99%), acetone (Chempur, 98%), toluene (Stanlab Sp. J., 98%), deuterated chloroform ($CDCl_3$, stab. with Ag, Sigma-Aldrich, ≥99.8 atom% D), deuterated dimethylsulfoxide (DMSO-d_6, Deutero, 99.8 atom% D), deuterated water (Sigma-Aldrich, 99.9 atom% D), deuterated sulfuric acid (D_2SO_4, Sigma-Aldrich, 96-98% solution in D_2O, 99.5 atom% D), were used as received.

FLEXZORB FM30K carbonaceous fabrics (AC fabrics) were obtained from CalgonCarbon company. Polypropylene (PP) fabrics were purchased from FECSA (Spain).

S2. Instrumentation

Powder X-ray diffraction (PXRD) data were collected on a Bruker D8 ADVANCE diffractometer equipped with a copper lamp (CuKα radiation, λ = 1.5406 Å) at 30 kV and 40 mA with a slit of 0.1°. Standard measurements were done in 2θ range of 4°-40° with a 2θ step of 0.008° and a counting time of 0.5 s.

^{1}H nuclear magnetic resonance (NMR) spectra in solutions were recorded using a Bruker Avance 500 spectrometer at 298 K and were calibrated on the residual solvent signal (DMSO-d_6: 2.50 ppm, $CDCl_3$: 7.26 ppm). The NMR samples of obtained MOFs before and after functionalization were prepared by digesting approximately 1 mg of dried material in D_2SO_4 and then diluting it with 0.6 ml of DMSO-d_6.

Solid-state MAS NMR ^{1}H-^{13}C CP MAS spectra were recorded at room temperature on a Bruker Avance III 11.7 T (125.75 MHz), Bruker Avance Neo 14 T (150.75 MHz), and Bruker Avance Neo 20 T (213.79 MHz) spectrometers equipped with 3.2 mm CPMAS probes and referenced to solid adamantane (38.48 ppm for the CH_2 signal)[2]. 55-80 kHz ^{1}H decoupling was used. Further experimental details are given in Table S1 (section S6).

Diffuse reflectance infrared Fourier transform (DRIFT) spectra were collected on Nicolet iS50 FT-IR Spectrometer (Thermo Scientific) with a Praying Mantis DRIFT accessory. The spectra were collected in a 4000-400 cm^{-1} range with number of scans set to 128. Samples were prepared under air atmosphere by grinding in a mortar with KBr and then placed under N_2 purge for collection time. Variable-temperature DRIFTS were collected using high temperature reaction chamber (Harrick Scientific Products Inc), accessory with temperature control performed with EZ-ZONE software under the N_2 atmosphere.

Nitrogen sorption isotherms were measured at 77 K on a Micromeritics ASAP 2020. Prior to the measurements, the samples were degassed at 120 °C for 24 h (the **initiator@MOF** samples were activated at 30 °C for 24 h). The Brunauer-Emmett-Teller (BET) theory was used to calculate the specific surface areas of obtained materials based on N_2 sorption measurements for collected samples. For all N_2 isotherm analyses we ensured that the two consistency criteria described by Roquerol et al.[3] and Walton et al.[4] were satisfied. The pore

sized distribution plots were derived from sorption data by DFT calculations using a carbon slit pore model with a N_2 kernel.

Scanning electron microscopy (SEM) images were collected on a Hitachi S-3400N-II variable-pressure scanning electron microscope. Samples were sputter-coated with 7 nm Au to facilitate viewing by SEM. Energy dispersive X-ray spectra (EDS) were obtained using an EDS Thermo Scientific Ultra Dry system.

Optical emission spectrometry with excitation by argon inductively coupled plasma (ICP-OES) was performed on the Thermo Fisher Scientific iCAP 7400 DUO instrument. The analyzed samples were dissolved in 1 ml of piranha solution (H_2SO_4/H_2O_2, v/v = 3/1) and diluted with distilled water.

Thermogravimetric analyses (TGA) were recorded on a Setaram SETSYS 16/18 instrument. Samples for thermogravimetric characterization were placed in alumina crucibles in synthetic air (O_2:N_2 = 20:80) (flow rate: 1 dm^3/h) at heating rate 5 ºC/min, samples were studied between 30 and 1000 ºC.

Matrix-assisted laser desorption/ionisation mass spectrometry (MALDI-MS) spectra were acquired in linear mode on JEOL JMS-S3000 SpiralTOF™-plus Ultra-High Mass Resolution MALDI-TOFMS. The 2 mg of NU-1000/PMMA was soaked in K_3PO_4 solution for 24 hours (to decompose the MOF network), the obtained residue was then dispersed in THF. The collected sample was then mixed with 2,5-dihydroxybenzoic acid (DHB, dissolved in THF, used as a matrix).

S3. Experimental procedures

S3.1. Synthesis of MOF materials

NU-1000: The material was synthesized according to the procedure described by Islamoglu et al. where benzoic acid and trifluoroacetic acid are used as co-modulators.[5]

MOF-808: The material was synthesized following the literature procedure with some minor modifications.[6] Typically, 1.08 g of zirconium oxide dichloride octahydrate ($ZrOCl_2 \times 8H_2O$, 3.35 mmol) and 0.745 g of 1,3,5-benzenetricarboxylic acid (H_3BTC, 3.55 mmol) were mixed in 32 ml of DMF. Then, 66 ml of formic acid was added to the reaction mixture, and sonicated for 10 min. The sealed reaction vessel was heated at 120 °C for 24 h. After that time, the white solid was formed. The product was centrifuged and washed thoroughly with DMF. Then the DMF was exchanged to acetone and soaked in that solvent overnight. The product was washed additionally with acetone (2 x 30 ml) and dried at 80 °C overnight.

The obtained Zr-MOFs were characterized by PXRD, ^{1}H NMR, DRIFTS, TGA and N_2 sorption measurements.

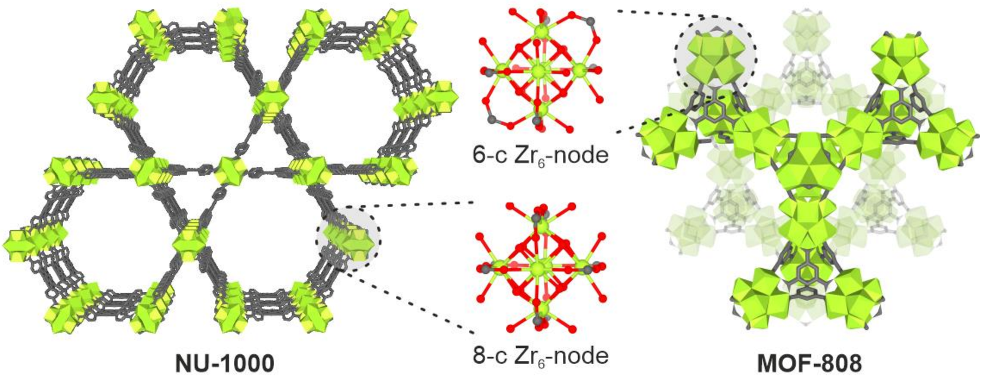

Figure S1. Structures of **NU-1000** (left) and **MOF-808** (right) materials with the 8-connected and 6-connected Zr_6-nodes, respectively.

S3.2. Preparation of initiator@MOF via SALI reaction

The SALI reaction with 4,4'-azobis(4-cyanovaleric acid) (ACPA) radical initiator was performed following the general literature procedure[7] with some modifications.

initiator@NU-1000: 250 mg of 4,4'-azobis(4-cyanovaleric acid) (0.891 mmol) was dissolved in 20 ml of ethanol in a falcon tube. Then, 200 mg (0.092 mmol) of **NU-1000** was added to the solution. The reaction was shaken at 25 °C (600 rpm, Eppendorf ThermoMixer) for 72 h. After that time, the solids were centrifuged, soaked in a fresh portion of ethanol (40 ml) and kept for another 24 h (25 °C, 600 rpm). Then the solid was washed with ethanol (3 × 30 ml) and acetone (2 × 30 ml) with short incubation (approx. 1 h) between washings to afford complete removal of the non-coordinated ACPA. The resulting solid was dried at 25 °C for 24 h under vacuum.

initiator@MOF-808: The SALI procedure was analogous to the described above for **initiator@NU-1000**. Typically, 100 mg of **MOF-808** (0.074 mmol), 200 mg of ACPA (0.714 mmol) and 10 ml of ethanol were used for one reaction.

The obtained **initiator@MOF** samples were characterized by PXRD, DRFITS, TGA and N_2 sorption measurements. The number of incorporated ACPA molecules was determined by ^{1}H NMR analysis of the **initiator@MOF** samples digested in a D_2SO_4/DMSO-d_6 mixture.

S3.3. Preparation of MOF/polymer hybrids

MOF/PMMA: Typically, 100 mg of **initiator@MOF** was placed in a Schlenk flask and degassed. Then, 4 ml of methyl methacrylate (MMA, 37.5 mmol) was added under N_2. The reaction vessel was sealed, and the obtained suspension was stirred at room temperature for 30 minutes. Then, the reaction was stirred at 70 °C for 48 h. The obtained product was thoroughly washed with methanol (to remove the excess of monomer). After that, the MOF/PMMA material was stirred in 100 ml of acetone at room temperature for at least 1 h. The obtained suspension was centrifuged (600 rpm, 15 min) and the remaining solid was washed several times with acetone. The final product was dried at 60 °C overnight.

MOF/PDMAM: Typically, 100 mg of **initiator@MOF** was placed in the Schlenk flask and degassed. Then, 5 ml of 2-dimethylaminoethyl methacrylate (DMAM, 29.6 mmol) was added under N_2. The reaction vessel was sealed, and the obtained suspension was stirred at room temperature for 30 minutes to afford uniform dispersion of monomer in the pores of the MOF. Then, the reaction was stirred at 50 °C for 48 h. The obtained products then undergo the same washing procedure with methanol and acetone, as described above for **MOF/PMMA**.

S3.4. Fabrics coating procedure

MOF/polymer/fiber composites were prepared by a wet deposition in THF. A suspension of MOF/PMMA or MOF/PDMAM in THF was added dropwise over the surface of polypropylene (PP) or activated carbon (AC) fabrics. Once the suspension homogeneously coated the fabric, it was dried at 70 °C for 15 min. Afterwards the fabric was turned over and a subsequent deposition carried out on the other side of the fabric, thus completing one cycle. A coloration in the fabric was visible in some cases (Fig. S2). The amount of Zr %wt on the fabric was determined by ICP-OES.

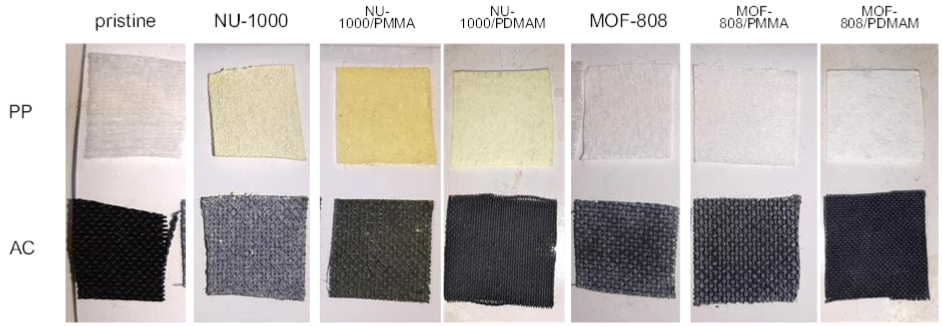

Figure S2. MOF/polymer/fiber composites prepared in this work.

S4. PXRD analysis

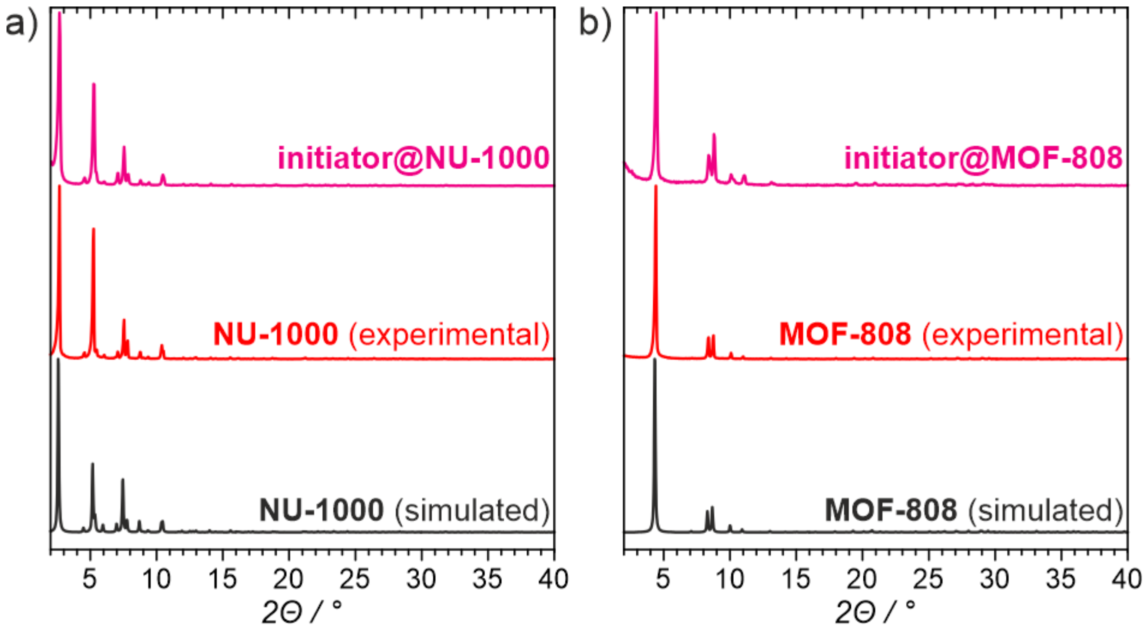

Figure S3. PXRD patterns of a) **NU-1000** before and after SALI reaction, b) **MOF-808** before and after SALI reaction with the radical polymerization initiator molecule.

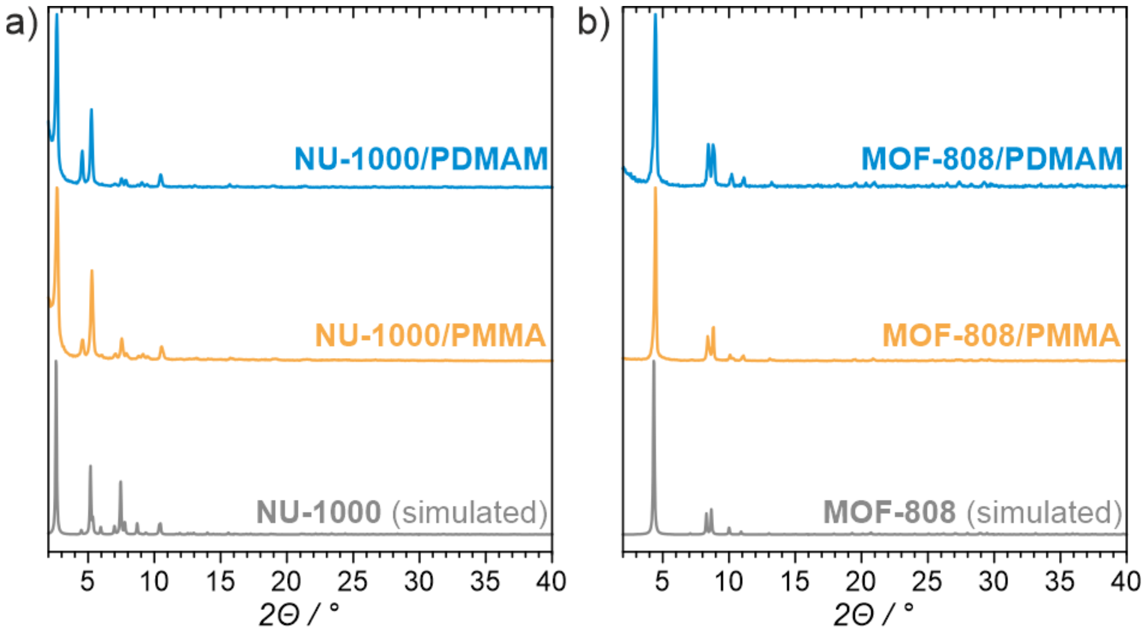

Figure S4. PXRD patterns of a) **NU-1000/polymer** hybrids and b) **MOF-808/polymer** hybrids.

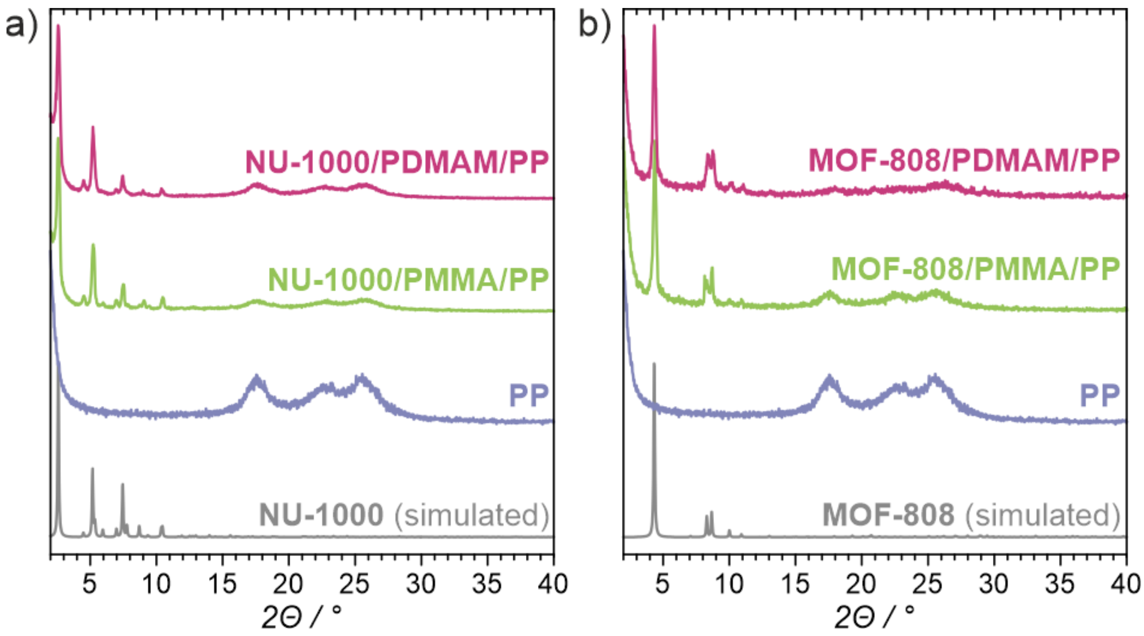

Figure S5. PXRD patterns of PP-based **MOF/polymer/fiber** composites with a) **NU-1000** and b) **MOF-808**.

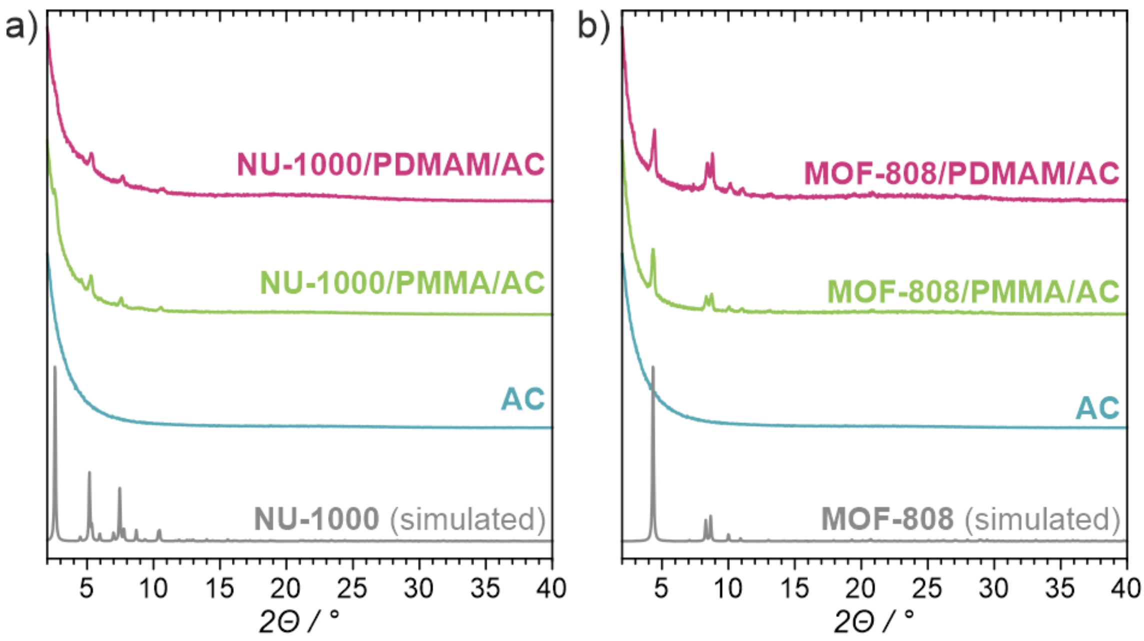

Figure S6. PXRD patterns of AC-based **MOF/polymer/fiber** composites with a) **NU-1000** and b) **MOF-808**.

S5. NMR analyses in solution

S5.1. NMR spectra of ACPA and its stability studies

The 4,4'-azobis(4-cyanovaleric acid) (ACPA) contains two stereogenic centers and forms different stereoisomers. Commercially available sources of ACPA typically contain a mixture of *rac*- and *meso*- diastereoisomers (Fig. S7)[8].

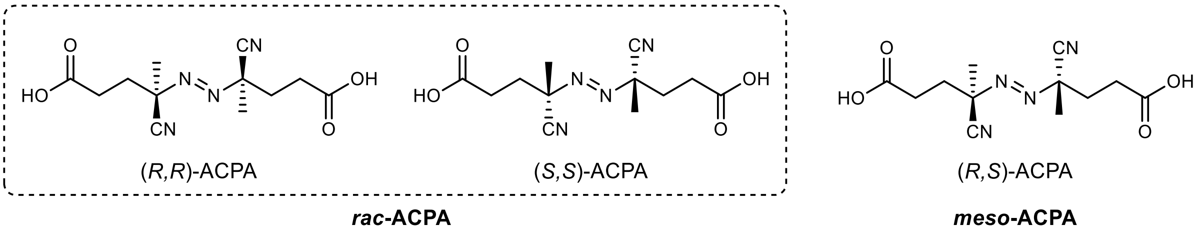

Figure S7. The structures of the isomers of ACPA molecule.

The ACPA content in the **initiator@MOF** samples was determined based on the relevant ^1H NMR spectra of the samples digested in D_2SO_4/DMSO-d_6 solution (v/v = 10/1, Fig. S10-S11). Before that, the spectra of ACPA initiator (in acid form) were recorded in different solvents, including D_2O, DMSO-d_6 and DMSO-d_6/D_2SO_4 solution (v/v = 10/1). The same pattern of signals was observed in all of the tested solutions, differing in the chemical shifts of the protons of the methyl groups, as shown on the Figures S8. Based on the literature NMR data[8] collected for the ACPA sodium salt, we assigned the singlet at 1.74 ppm to the protons of the methyl group of *meso*-ACPA and the singlet at 1.68 ppm to its racemic form (*rac*-ACPA) in the sample measured in deuterated water (Fig. S8a). It is worth noting, that the addition of deuterated sulfuric acid shifts the ACPA peaks to lower frequencies, which we attributed to the possible protonation of the nitrogen atoms of ACPA (Fig. S8c).

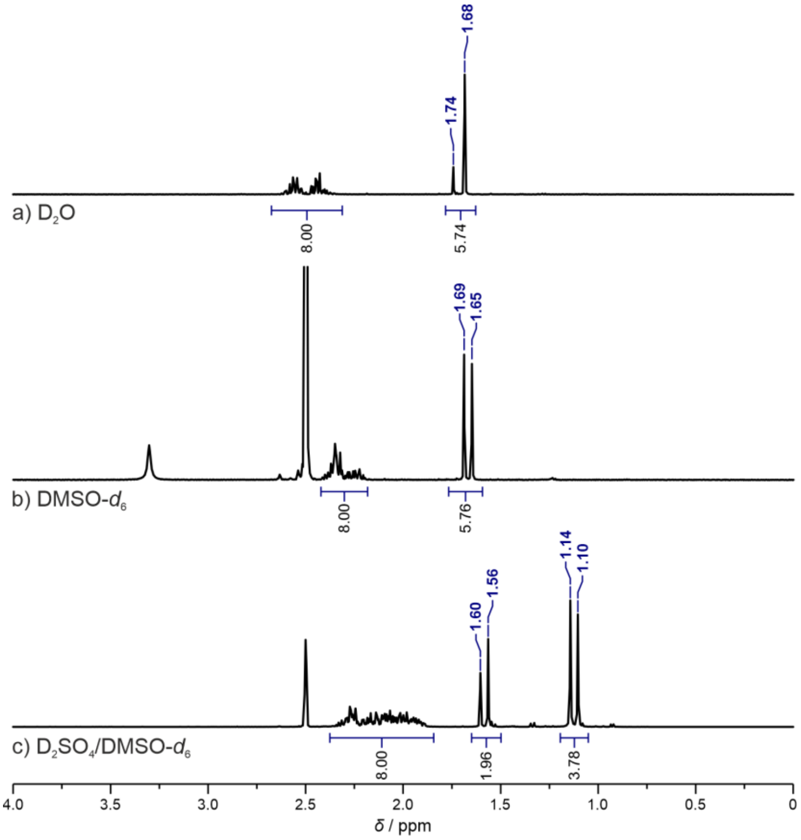

Figure S8. ^1H NMR spectra of ACPA initiator measured in various deuterated solvents: a) D_2O, b) DMSO-d_6 and c) mixture of D_2SO_4/DMSO-d_6 (v/v = 1/10).

The ^{1}H NMR spectrum of ACPA in its native form was compared with the spectrum of the ACPA molecule after heating it at 70 °C for 24 hours. On the recorded spectrum (Fig. S9b), new sets of signals were observed which can be assigned to the different decomposition products of ACPA initiator[8].

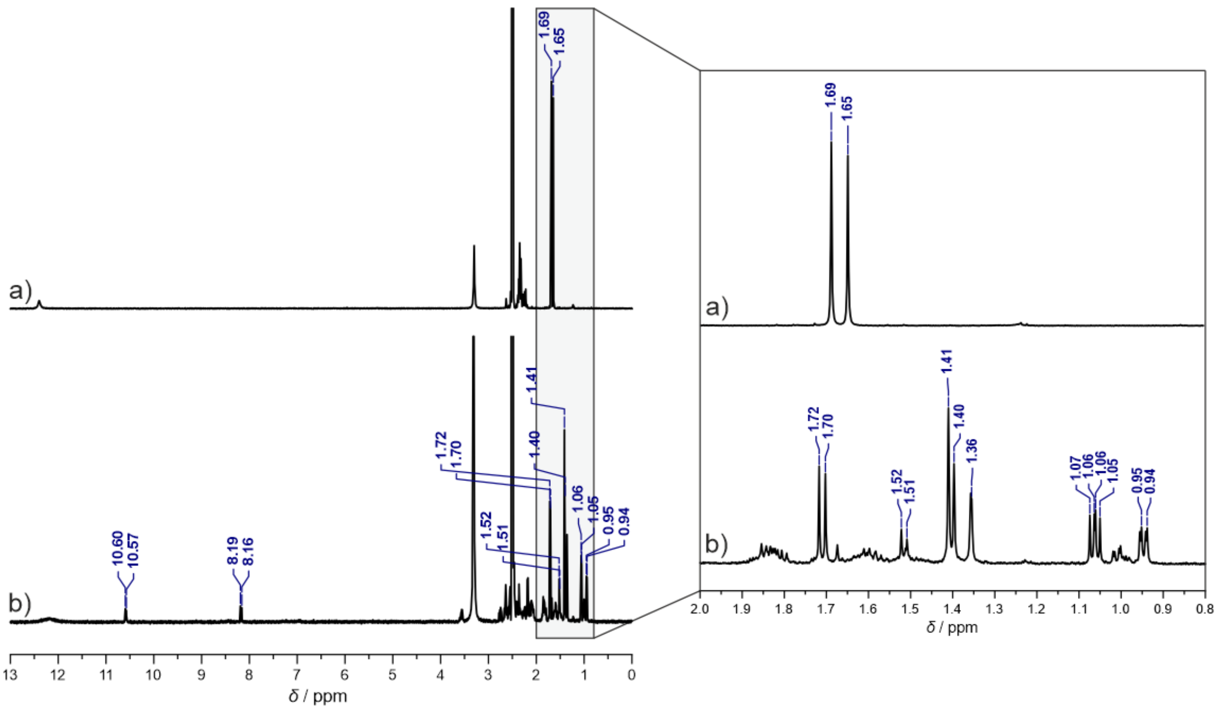

Figure S9. ^{1}H NMR spectra of ACPA initiator in DMSO-d_6 before (a) and after heating at 70 °C for 24 h (b).

S5.2. ^{1}H NMR spectra of initiator@MOF samples

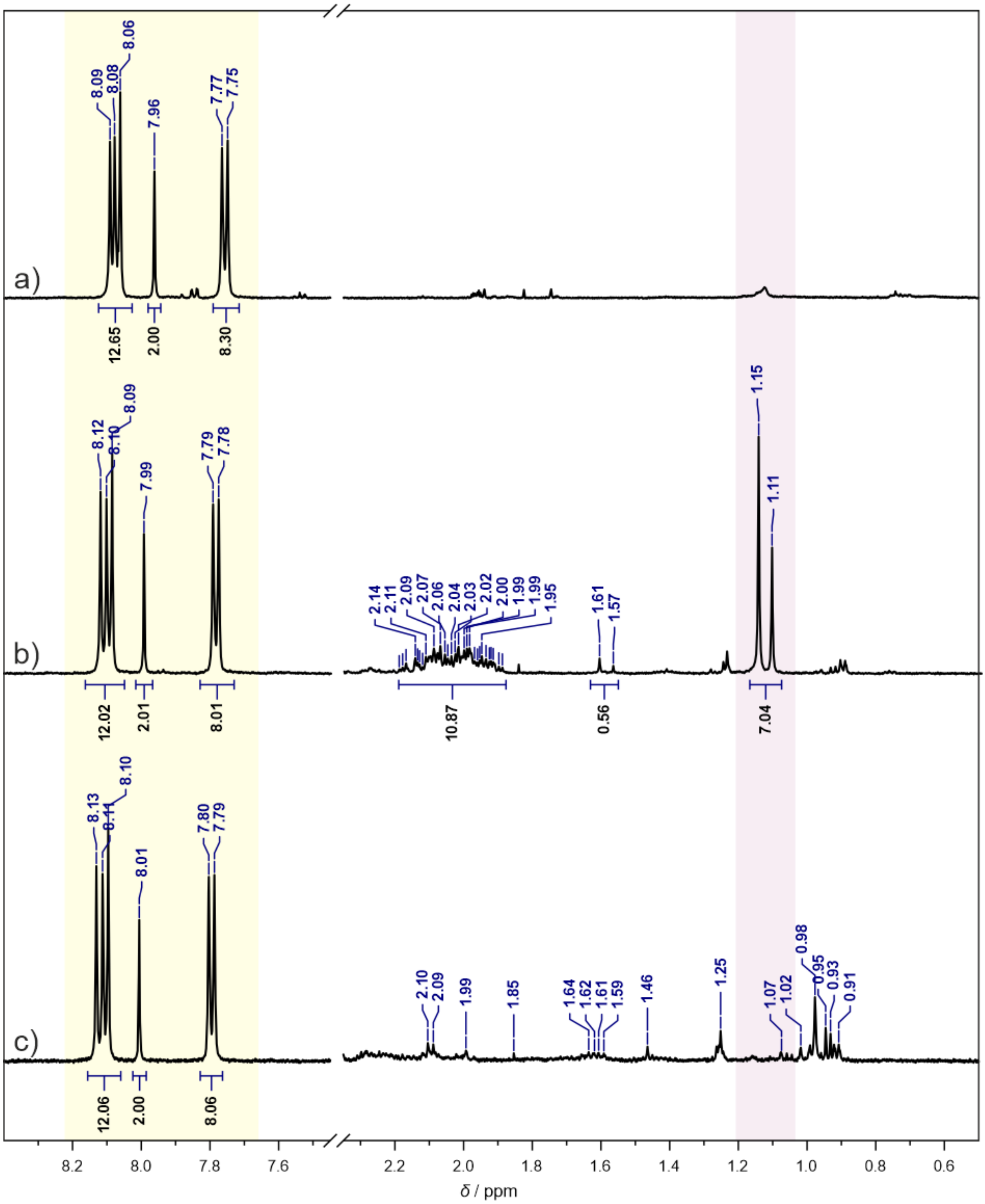

Figure S10. ^{1}H NMR of **NU-1000** and **initiator@NU-1000** materials digested in D_2SO_4/DMSO-d_6 solution: a) **NU-1000** material before SALI reaction, b) **initiator@NU-1000** material dried at room temperature, c) **initiator@NU-1000** after heating in 120 °C for 24 hours. The proton signals annotated with yellow color come from the TBAPy^{4-} linker of **NU-1000** material, violet color annotates the proton signals of methyl group of ACPA initiator molecules.

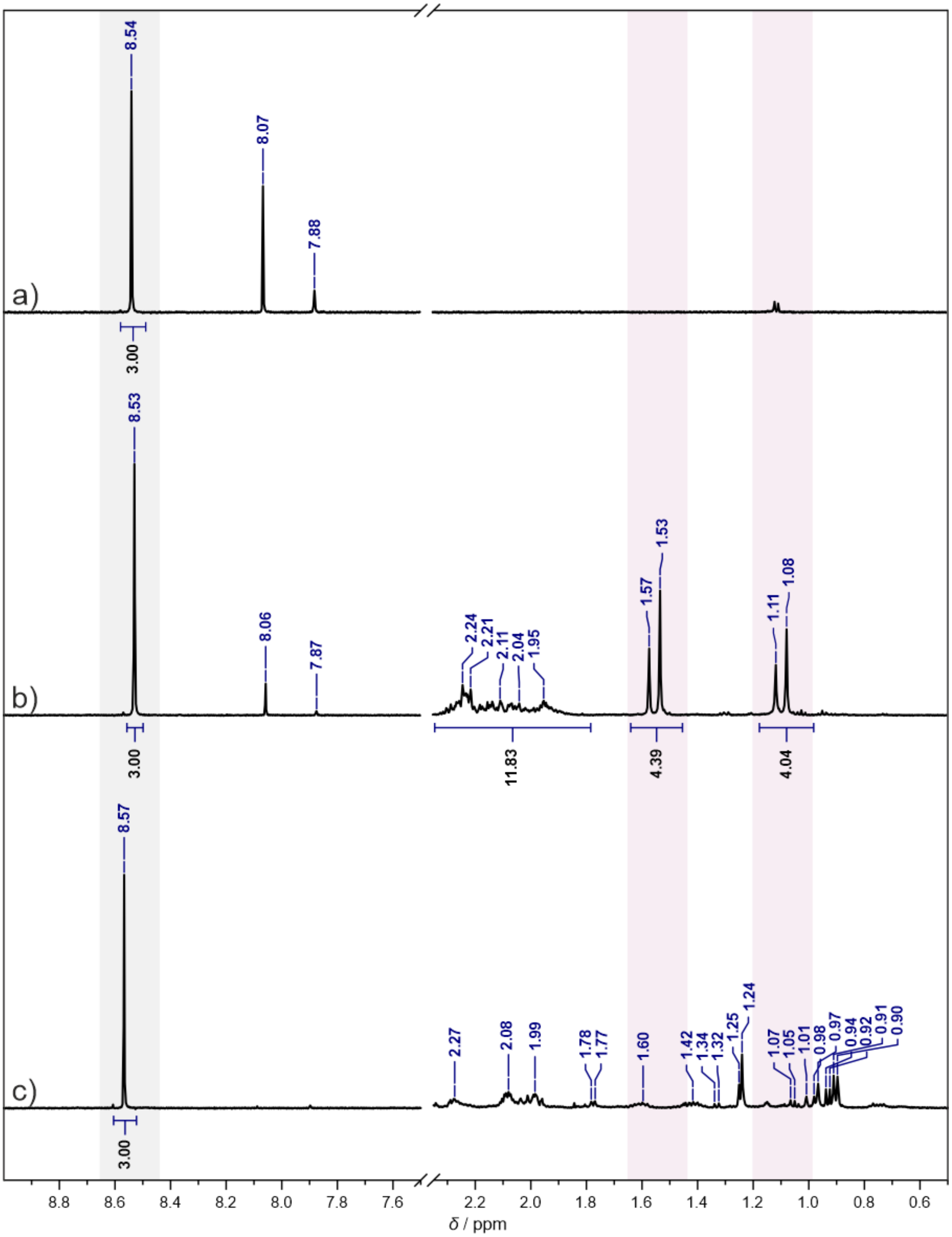

Figure S11. ^{1}H NMR of **MOF-808** and **initiator@MOF-808** digested in D_2SO_4/DMSO-d_6 solution: a) **MOF-808** before SALI reaction, b) **initiator@MOF-808** dried at room temperature, c) **initiator@MOF-808** after heating in 120 °C for 24 h. The proton signal annotated with grey color comes from the BTC^{3-} linker of **MOF-808**, violet color annotates the proton signals of methyl group of ACPA initiator.

S5.3. ^{1}H NMR spectra of monomers loaded in initiator@MOF samples

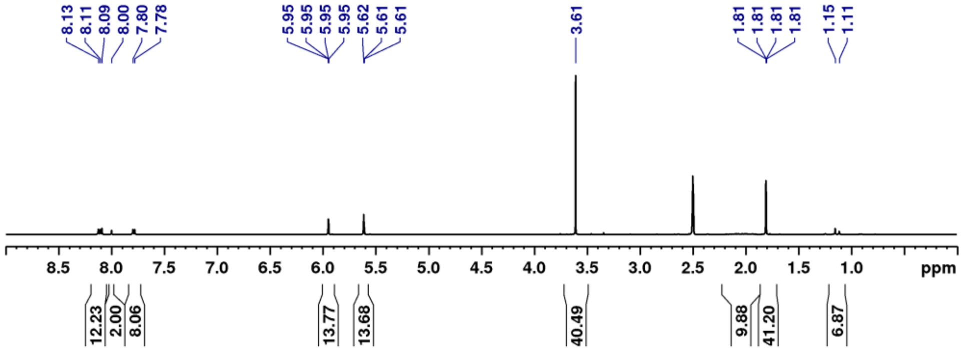

Figure S12. ^{1}H NMR of MMA loaded from vapour phase at room temperature in **initiator@NU-1000**. The calculated loading is 27 MMA molecules per Zr_6-node.

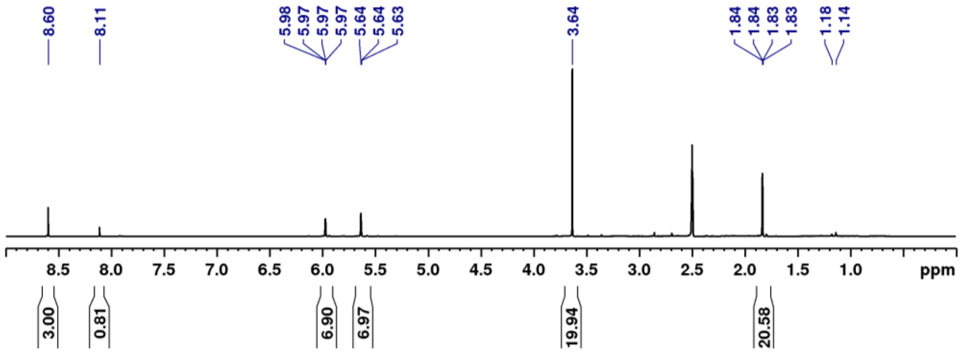

Figure S13. ^{1}H NMR of MMA loaded from vapour phase at room temperature in **initiator@MOF-808**. The calculated loading is 14 MMA molecules per Zr_6-node.

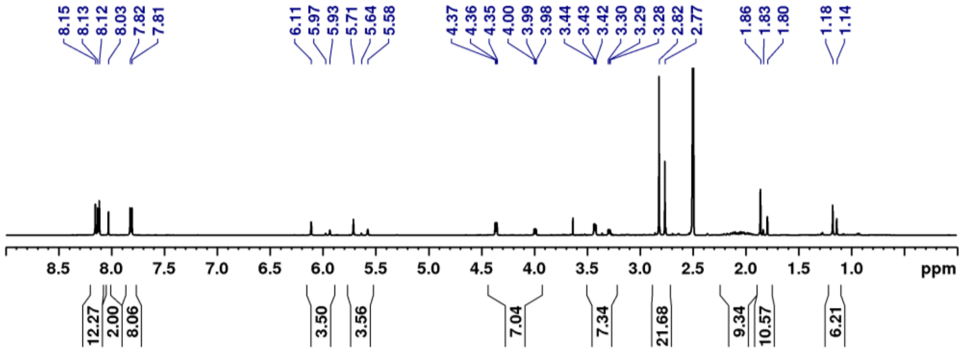

Figure S14. ^{1}H NMR of DMAM loaded from vapour phase at room temperature in **initiator@NU-1000**. The calculated loading is 7 DMAM molecules per Zr_6-node.

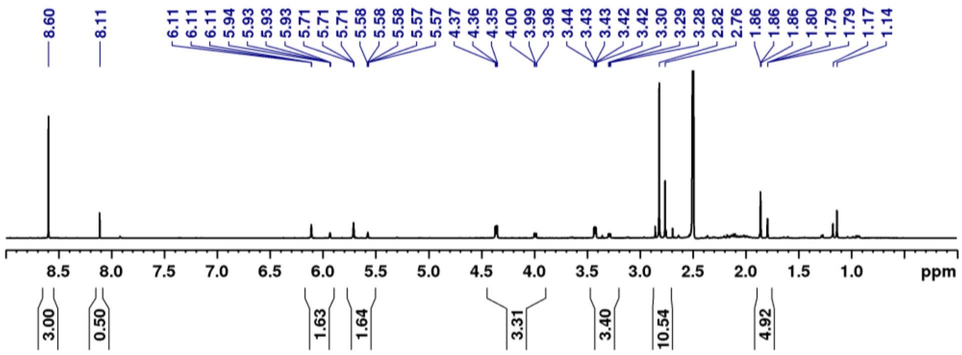

Figure S15. ^{1}H NMR of DMAM loaded from vapour phase at room temperature in **initiator@MOF 808**. The calculated loading is 3 DMAM molecules per Zr_6-node.

S6. Solid-state ^{13}C CP MAS NMR spectra of the materials

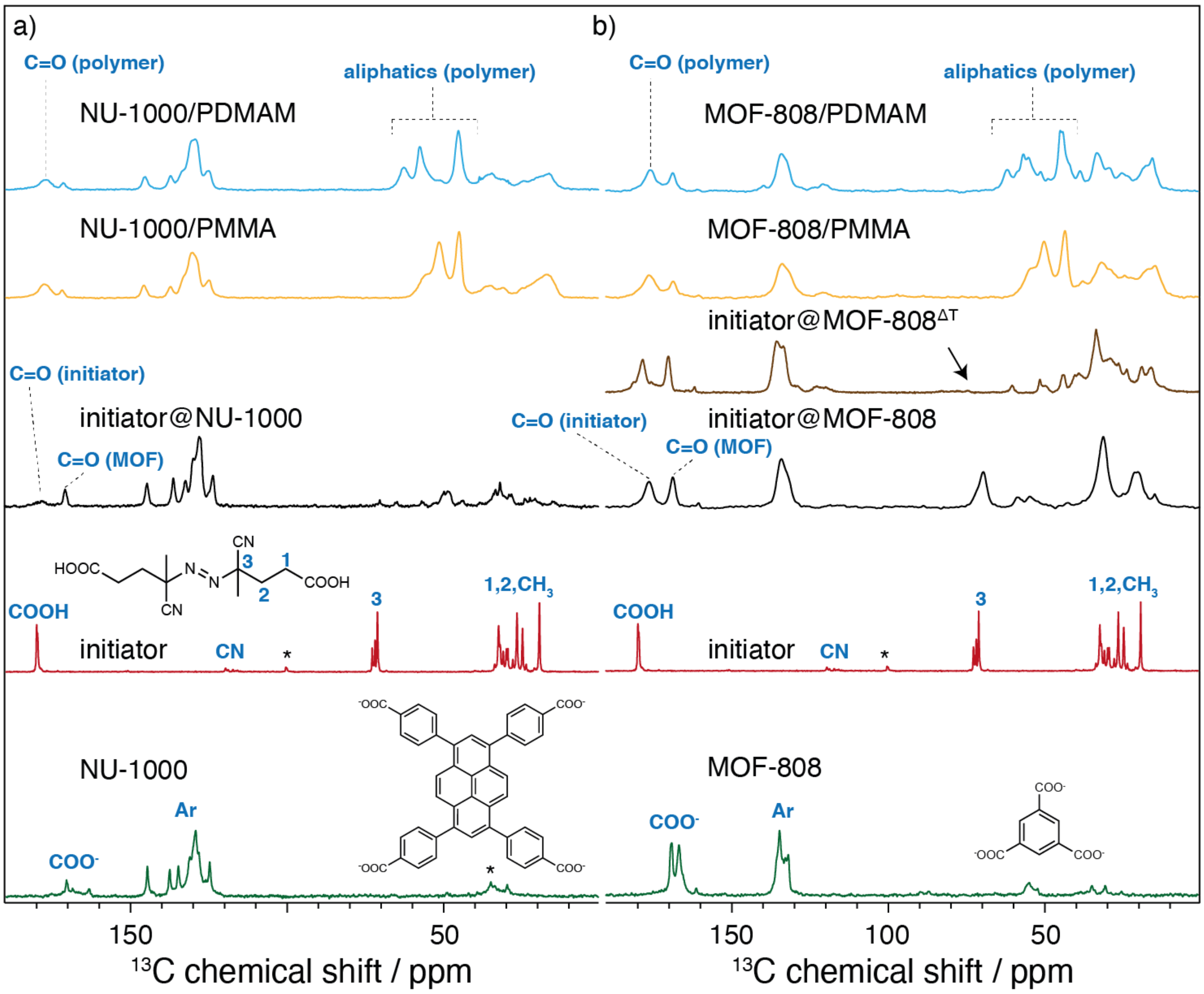

Figure S16. ^{13}C CP-MAS spectra of a) the **NU-1000**, and b) **MOF-808** family of materials. The structures of the corresponding linkers are shown at the bottom. Asterisks indicate spinning sidebands.

Table S1. Acquisition and processing details of the solid-state NMR spectra. All experiments were recorded at 20 kHz MAS using cross-polarization.

material	^1H T_1 [s]	Recycle delay [s]	Number of scans	Magnetic field [T]	Apodization [Hz]
initiator (ACPA)	1.4	2.0	612	20	5
NU-1000	0.8	1.0	3434	11.7	20
initiator@NU-1000	0.6	0.8	3786	14	50
NU-1000/PMMA	0.6	0.8	5462	20	50
NU-1000/PDMAM	1.0	1.5	6104	20	50
MOF-808	0.4	0.5	3554	14	20
initiator@MOF-808	0.3	0.5	5477	14	100
initiator@MOF-808$^{\Delta T}$	0.3	0.5	150126	14	10
MOF-808/PMMA	0.6	0.8	7217	20	50
MOF-808/PDMAM	1.0	1.5	6104	20	50

S7. DRIFT spectra

S7.1. DRIFT spectra of the ACPA initiator and its thermal stability studies

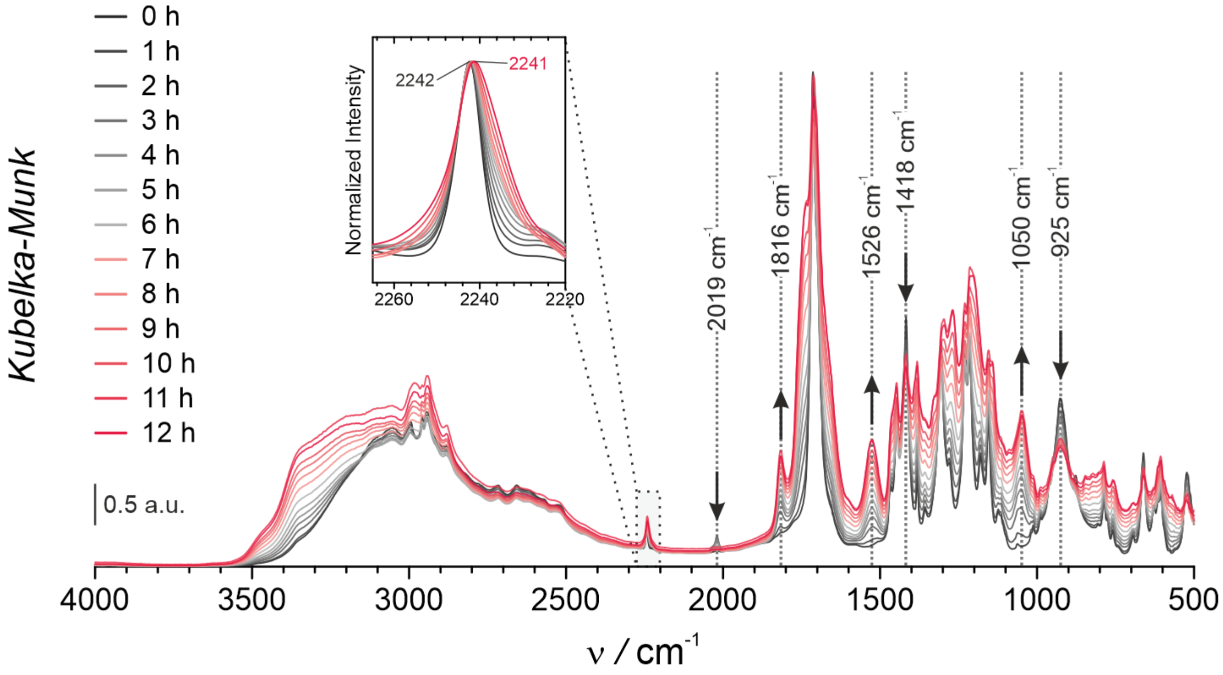

Figure S17. The DRIFT spectra of ACPA measured at 70 °C for 12 h (spectra recorded every 1 hour). Black curve represents the starting sample of ACPA, the red curve represents the sample after incubation at 70 °C for 12 h. The observed increase/decrease of selected bands over time is annotated with arrows. Inset shows a normalized intensity of the ν(C≡N) band vibration region.

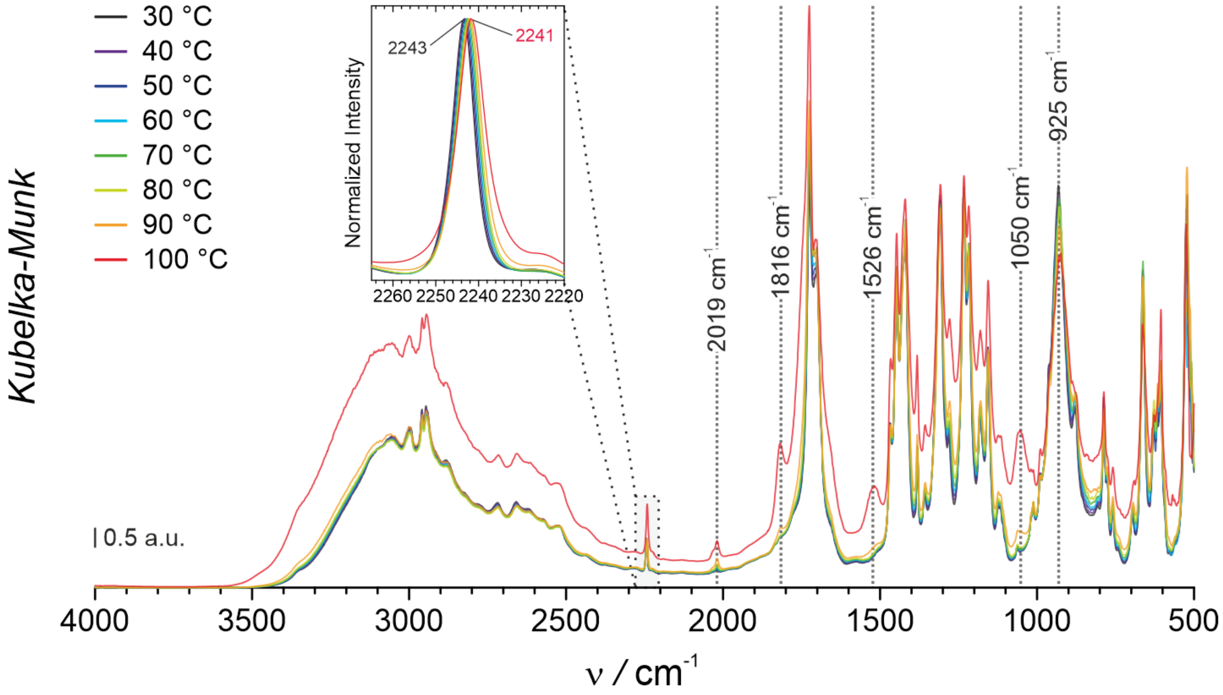

Figure S18. The VT-DRIFT analysis of ACPA, the spectra were measured in the temperature range of 30 - 100 °C (spectra were collected every 10 °C, the heating rate was 5 °C/min, prior to measurement the sample was incubated at given temperature for 5 min). The observed changes of the intensities of selected bands are annotated. The inset shows a normalized intensity of the ν(C≡N) band region.

S7.2. DRIFT spectra of initiator@MOF samples

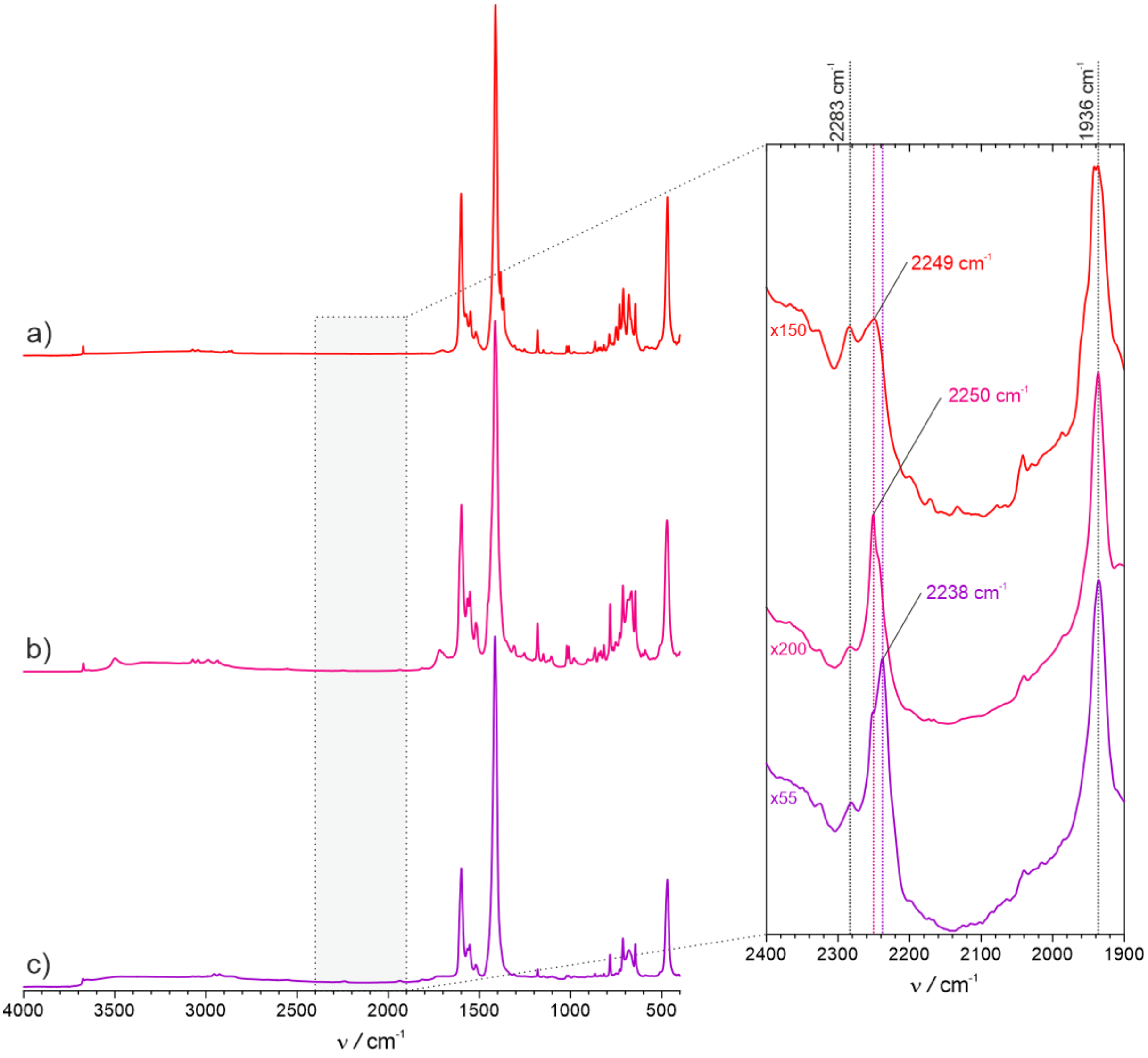

Figure S19. Normalized DRIFT spectra of **NU-1000** and **initiator@NU-1000**: a) pristine **NU-1000**, b) **initiator@NU-1000** dried at room temp., c) **initiator@NU-1000** after heating at 120 °C for 24 h (**initiator@NU-1000$^{\Delta T}$**). The inset shows the zoomed spectra in the range of 2400 - 1900 cm^{-1} (C≡N vibrations). For a better clarity, the spectra were normalized with respect to the band at 1936 cm^{-1}.

For **initiator@NU-1000** the increase of the intensity of the band at 2250 cm^{-1} was observed during heating, and the shift to 2238 cm^{-1} of the respective band was observed, which can be assigned to the C≡N group vibration of the decomposition products of ACPA initiator.

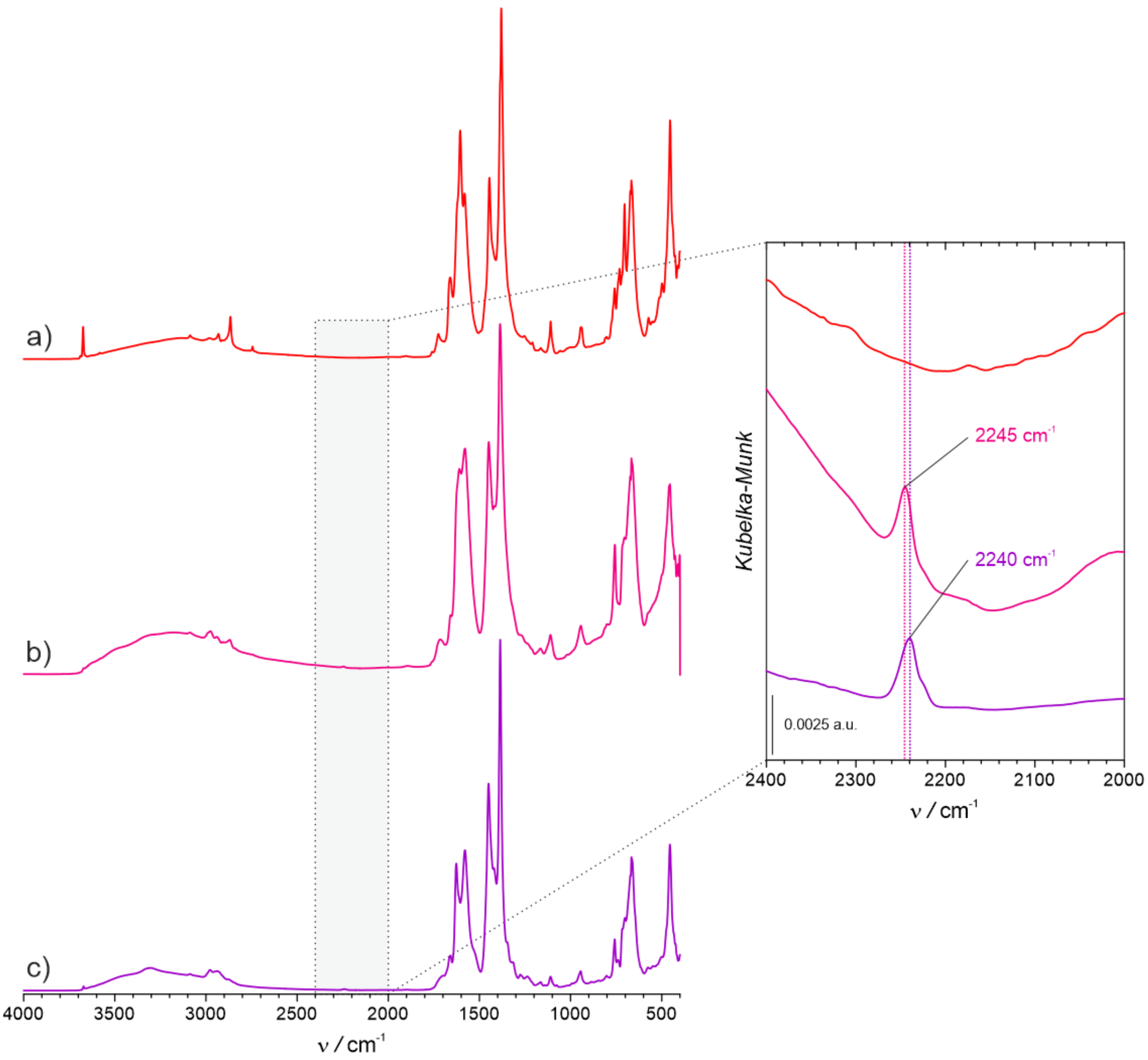

Figure S20. Normalized DRIFT spectra of **MOF-808** and **initiator@MOF-808**: a) pristine **MOF-808**, b) **initiator@MOF-808** dried at room temp., c) **initiator@MOF-808** after heating at 120 °C for 24 h (**initiator@MOF-808$^{\Delta T}$**). The inset shows the zoomed spectra in the range of 2400 - 1850 cm^{-1} (C≡N vibrations). For a better clarity, the spectra were normalized with respect to the band at 1900 cm^{-1}.

For **initiator@MOF-808** the increase of the intensity of the band at 2245 cm^{-1} was observed during heating, and the shift to 2240 cm^{-1} of the respective band was observed, which can be assigned to the C≡N group vibration of the decomposition products of ACPA initiator.

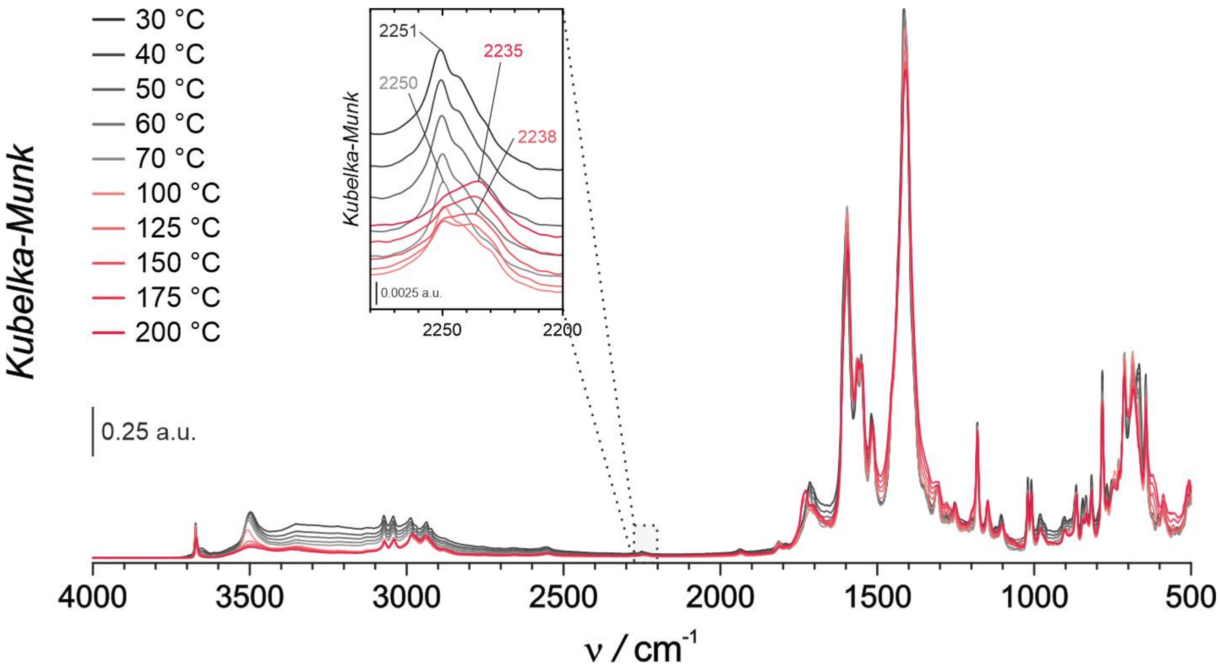

Figure S21. The VT-DRIFT spectra of **initiator@NU-1000**. The spectra were measured in the temperature range of 30-200 °C with the heating rate of 5 °C/min (prior to measurement the sample was incubated at given temperature for 5 min.). The inset shows the region of C≡N vibration band.

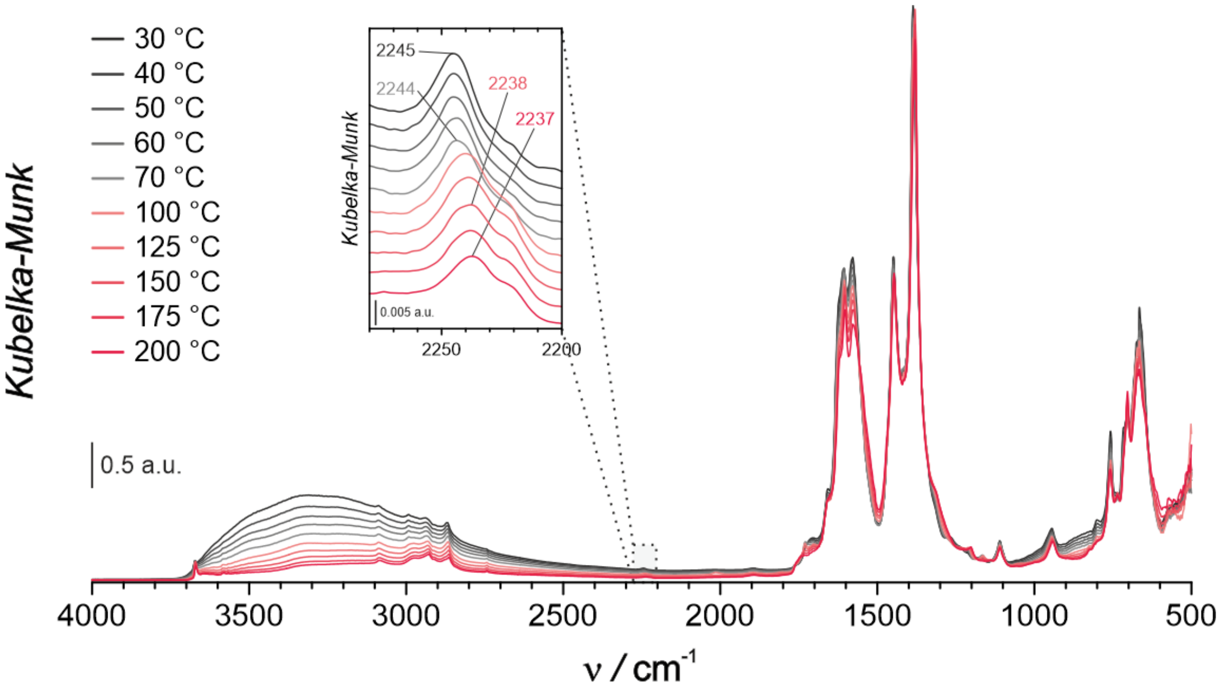

Figure S22. The VT-DRIFT spectra of **initiator@MOF-808**. The spectra were measured in the temperature range of 30-200 °C with the heating rate of 5 °C/min (prior to measurement the sample was incubated at given temperature for 5 min.). The inset shows the region of C≡N vibration band.

S7.3. DRIFT spectra of MOF/polymer samples

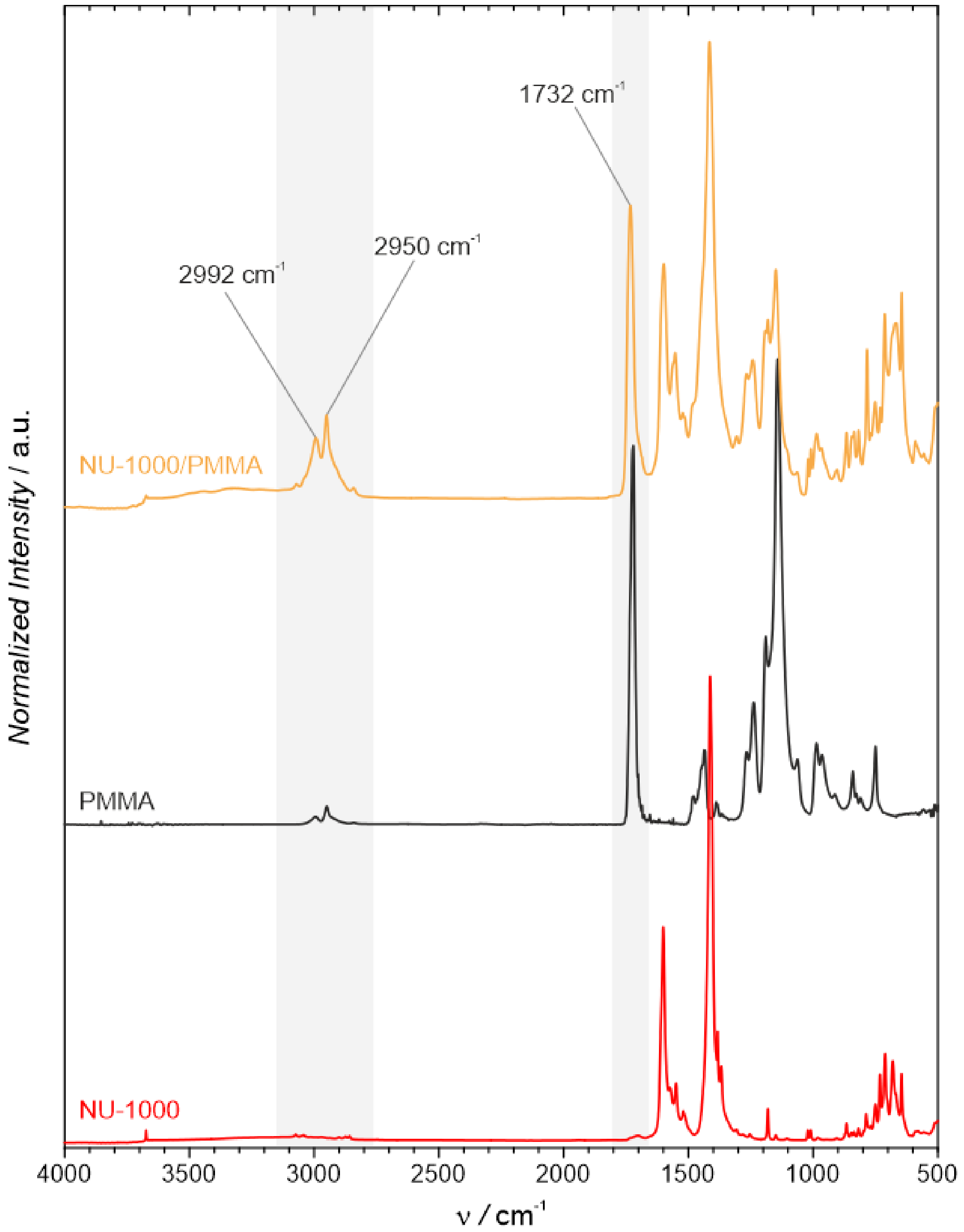

Figure S23. Normalized DRIFT spectrum of **NU-1000/PMMA** material. The spectra of **NU-1000** (DRIFT, red line) and **PMMA** (ATR, black line) are shown for comparison The annotated part of the spectra around 3000 cm^{-1} and 1730 cm^{-1} highlights the characteristic *v*(C-H) and *v*(C=O) vibrations of the polymer chains, respectively.

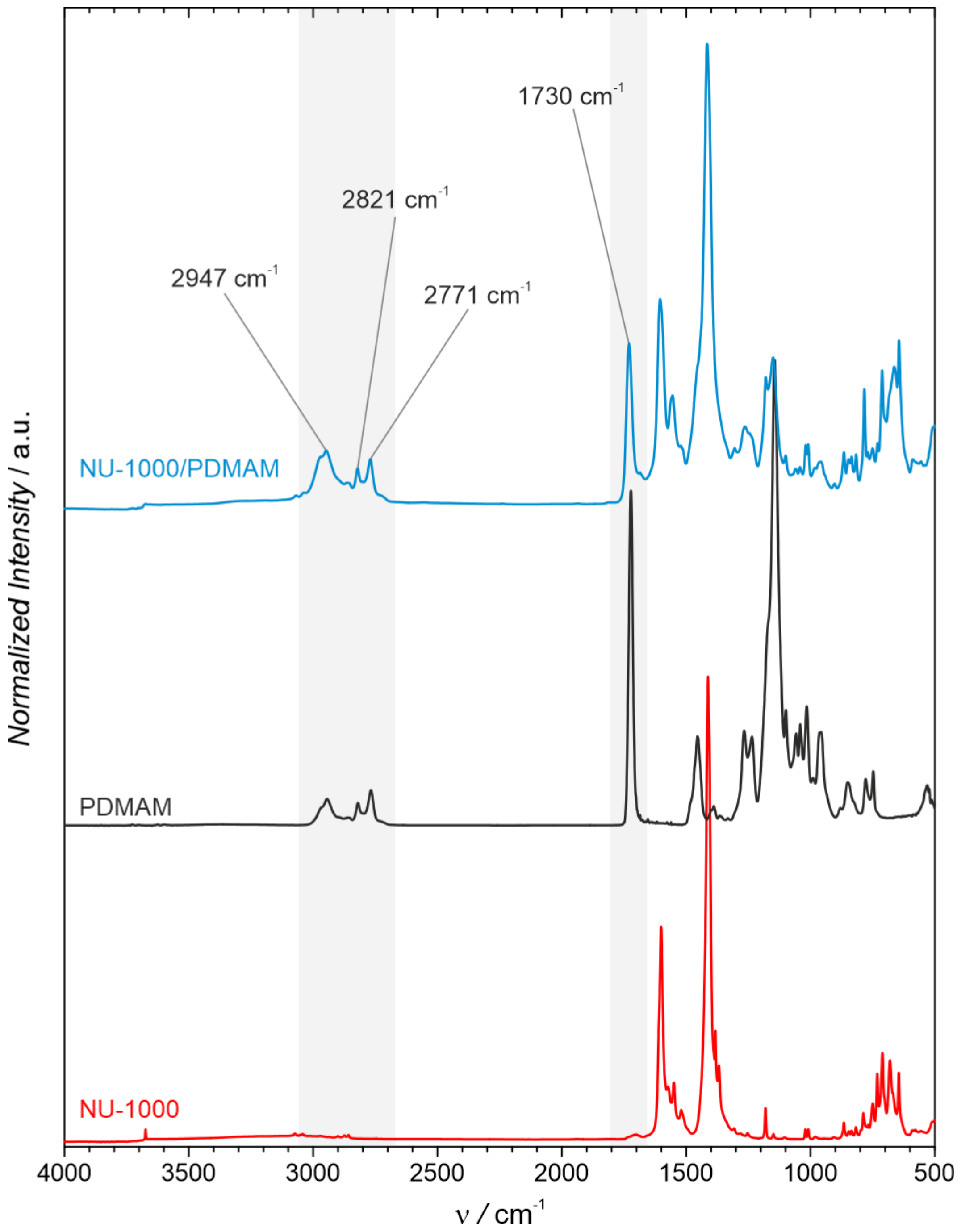

Figure S24. Normalized DRIFT spectrum of **NU-1000/PDMAM**. The spectra of **NU-1000** (DRIFT, red line) and **PDMAM** (ATR, black line) are shown for comparison The annotated part of the spectra around 3000 cm^{-1} and 1730 cm^{-1} highlights the characteristic ν(C-H) and ν(C=O) vibrations of the polymer chains, respectively.

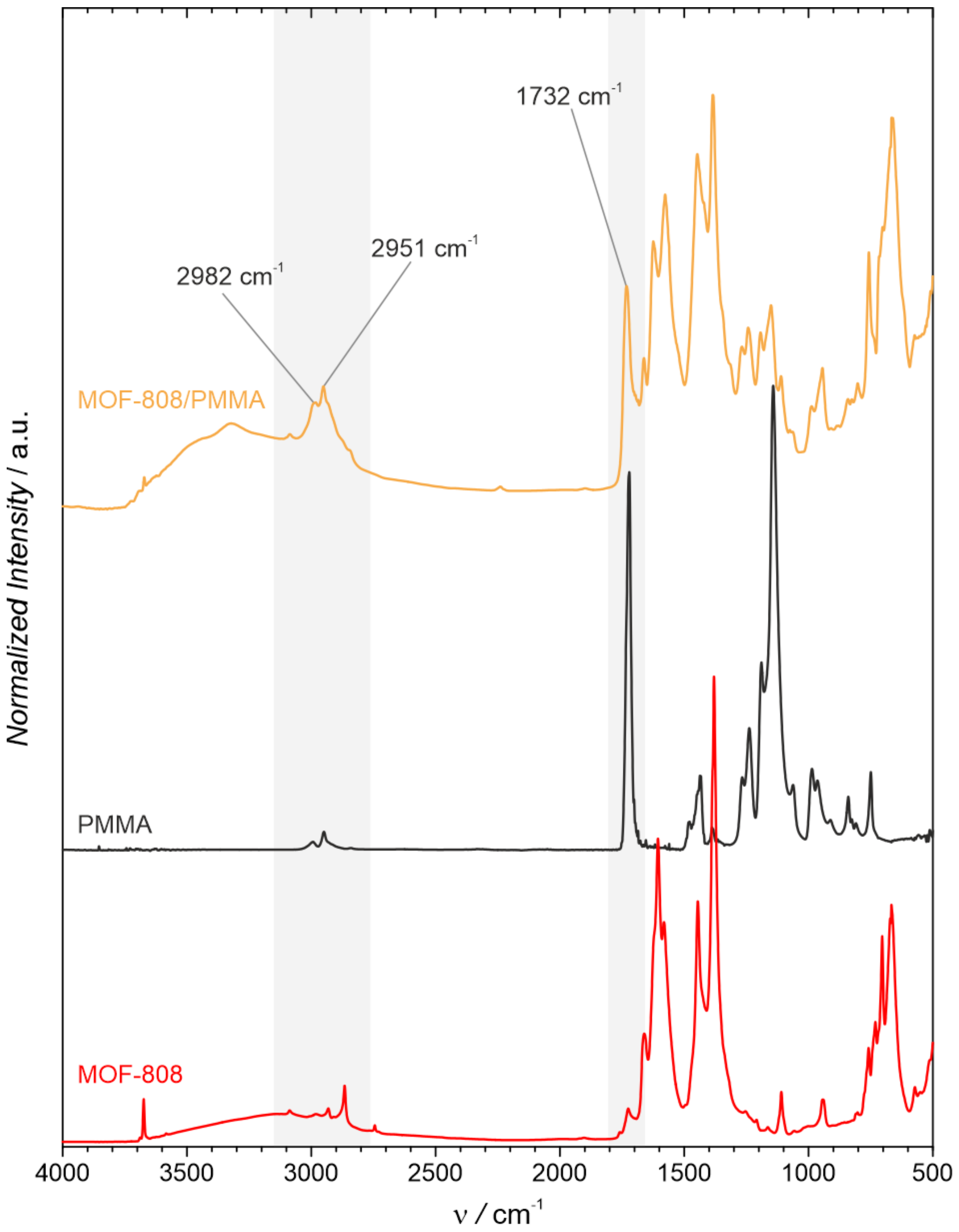

Figure S25. Normalized DRIFT spectrum of **MOF-808/PMMA**. The spectra of **MOF-808** (DRIFT, red line) and **PMMA** (ATR, black line) are shown for comparison The annotated part of the spectra around 3000 cm^{-1} and 1730 cm^{-1} highlights the characteristic *v*(C-H) and *v*(C=O) vibrations of the polymer chains, respectively.

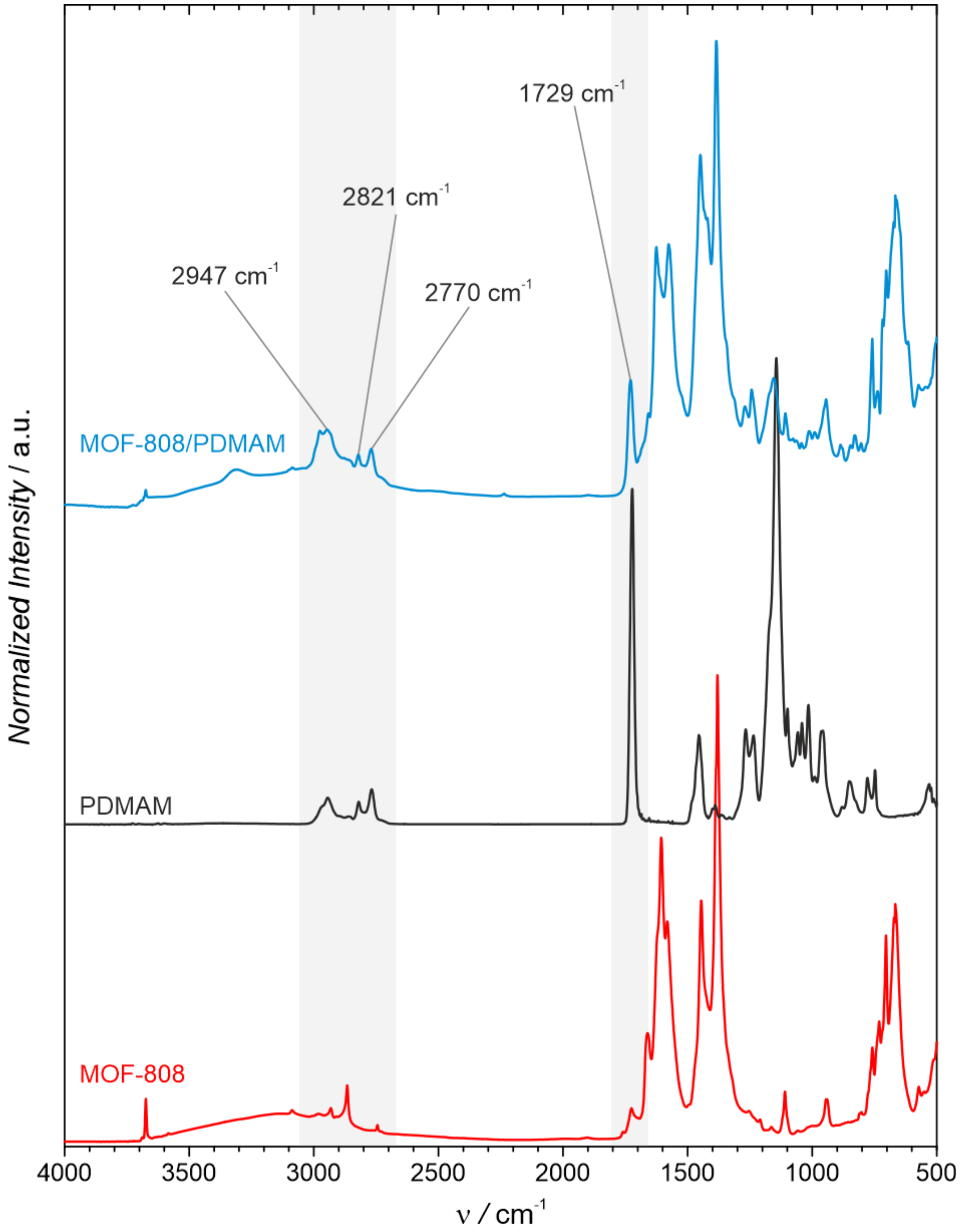

Figure S26. Normalized DRIFT spectrum of **MOF-808/PDMAM** material. The spectra of **MOF-808** (DRIFT, red line) and **PDMAM** (ATR, black lines) are shown for comparison The annotated part of the spectra around 3000 cm^{-1} and 1730 cm^{-1} highlights the characteristic ν(C-H) and ν(C=O) vibrations of the polymer chains, respectively.

S8. Sorption studies

S8.1. N_2 sorption isotherms

The Brunauer-Emmett-Teller (BET) theory was used to calculate the specific surface areas of obtained materials. For all isotherm analyses we ensured that the consistency criteria described by Roquerol et al.[3] and Walton et al.[4] were satisfied. The obtained BET surfaces areas are collected in **Table S2**. The pore size distribution (PSD) plots were derived from sorption data by the DFT method using a carbon slit pore model with the N_2 kernel.

Table S2. BET parameters and sorption data for Zr-based materials.

sample	*C*	Q_m (cm^3/g STP)	S_{BET} (m^2/g)	V_{pore} (cm^3/g)[a]
**NU-1000**	397.3	471.4	2052	1.39
**initiator@NU-1000**	136.3	422.3	1838	1.10
**NU-1000/PMMA**	169.8	161.3	702	0.35
**NU-1000/PDMAM**	146.0	158.6	691	0.34
**MOF-808**	354.5	259.0	1127	0.81
**initiator@MOF-808**	2310.3	182.5	794	0.36
**MOF-808/PMMA**	1389.4	108.3	471	0.22
**MOF-808/PDMAM**	1390.6	19.6	85	0.04

[a] Total pore volume calculated from single-point adsorbate uptake at the relative pressure, $p/p_0 = 0.96$.

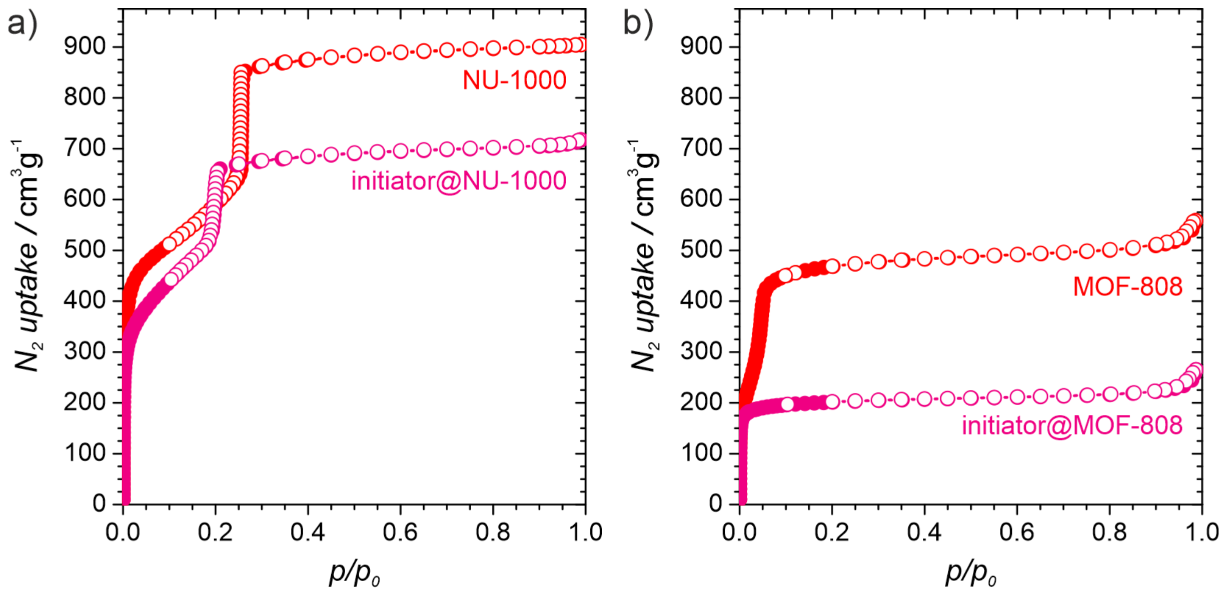

Figure S27. N_2 sorption isotherms measured at 77 K for: a) pristine **NU-1000** and **initiator@NU-1000**, b) pristine **MOF-808** and **initiator@MOF-808** (filled symbols - adsorption, open symbols - desorption).

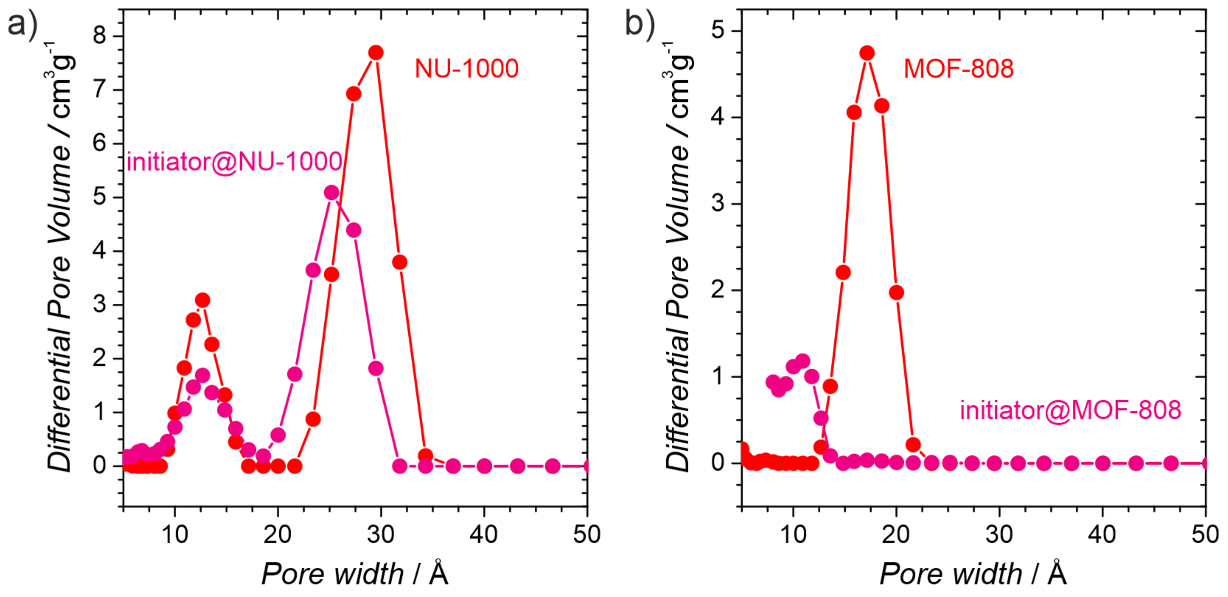

Figure S28. Density functional theory (DFT) pore size distribution (PSD) of selected Zr-MOFs before and after SALI functionalization with ACPA: a) **NU-1000** and **initiator@NU-1000** and b) **MOF-808** and **initiator@MOF-808**.

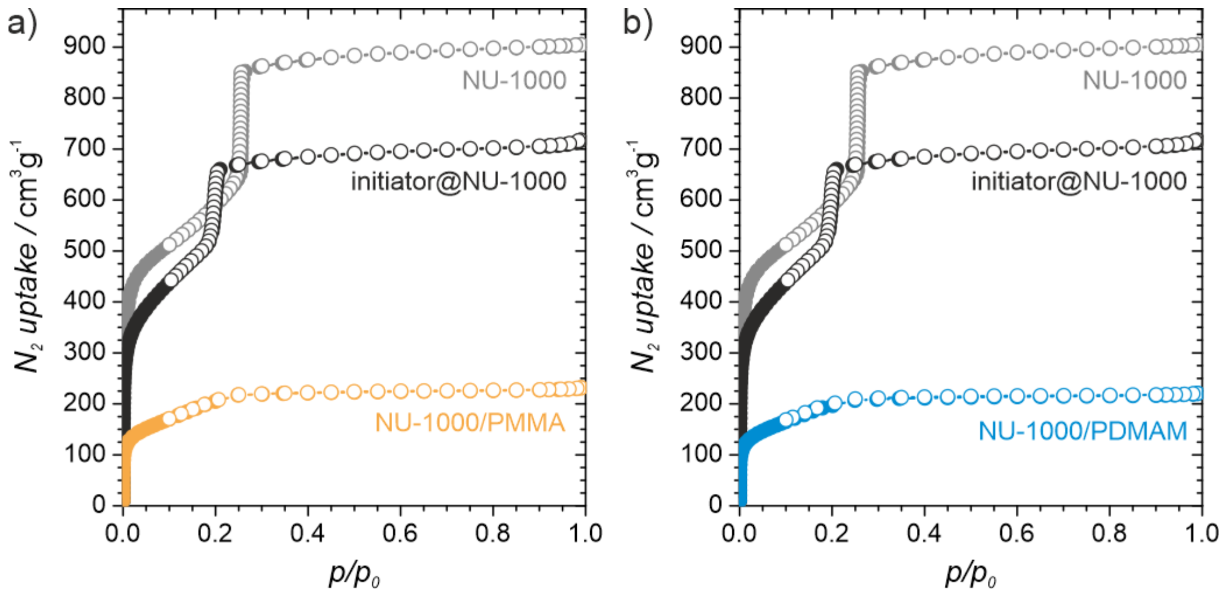

Figure S29. N_2 sorption isotherms measured at 77 K for: a) **NU-1000/PMMA** and b) **NU-1000/PDMAM**. The isotherms of **NU-1000** and **initiator@NU-1000** are shown for comparison (filled symbols - adsorption, open symbols - desorption).

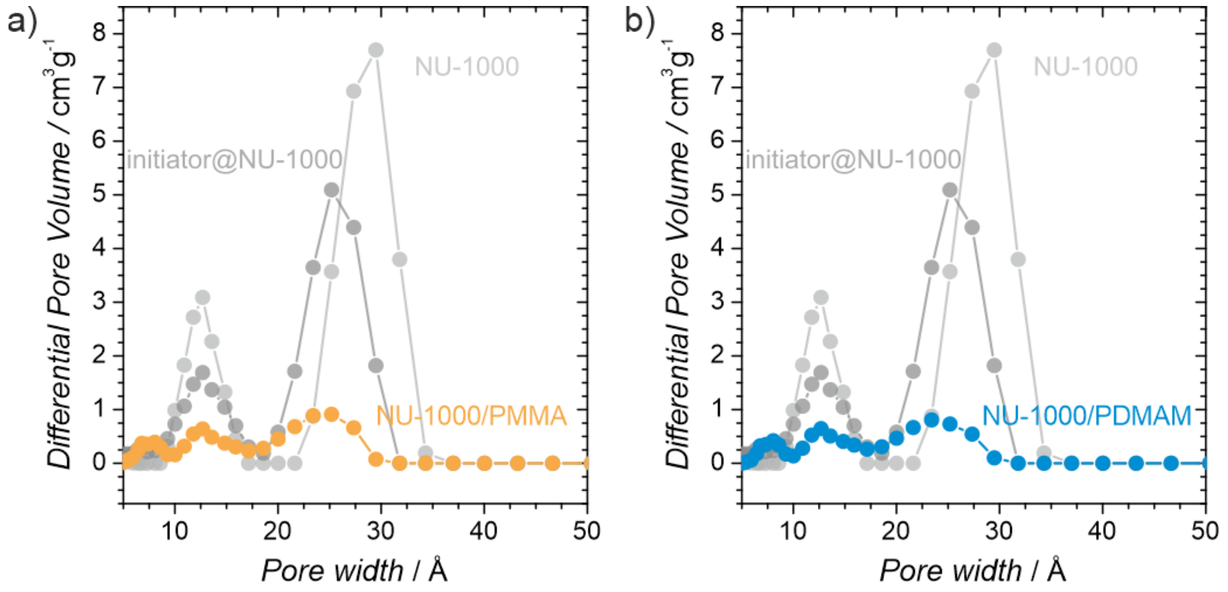

Figure S30. Density functional theory (DFT) pore size distribution (PSD) for: a) **NU-1000/PMMA** and b) **NU-1000/PDMAM**. The PSDs of **NU-1000** and **initiator@NU-1000** are shown for comparison.

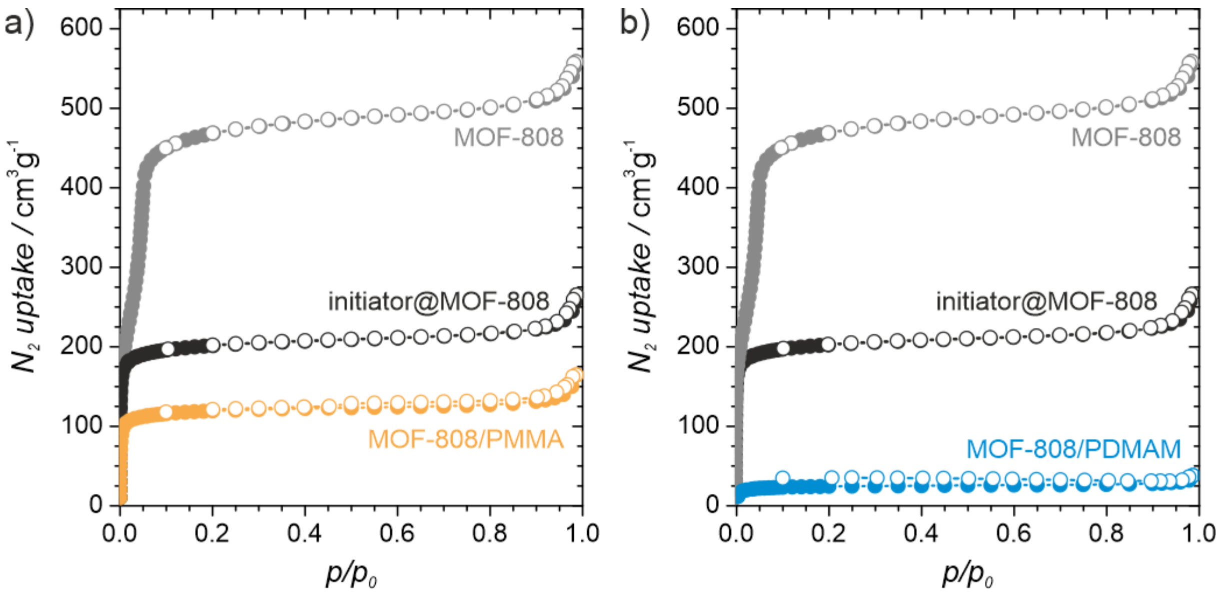

Figure S31. N_2 sorption isotherms measured at 77 K for a) **MOF-808/PMMA** and b) **MOF-808/PDMAM**. The isotherms of **MOF-808** and **initiator@MOF-808** are shown for comparison (filled symbols - adsorption, open symbols - desorption).

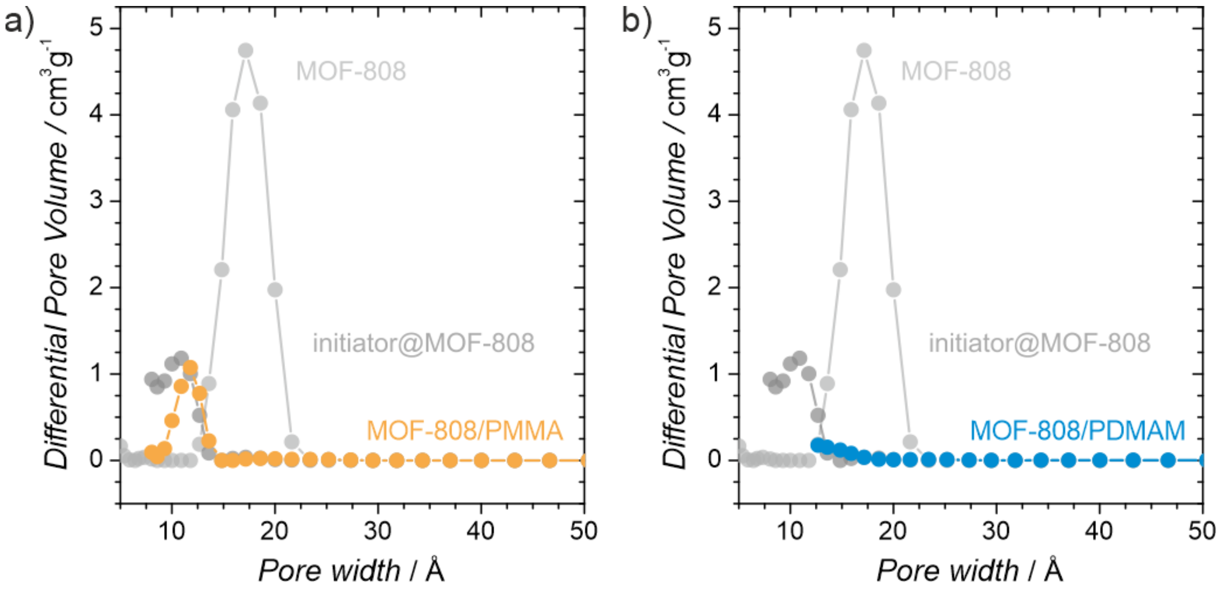

Figure S32. Density functional theory (DFT) pore size distribution (PSD) of a) **MOF-808/PMMA** and b) **MOF-808/PDMAM**. The PSDs of **MOF-808** and **initiator@MOF-808** are shown for comparison.

S8.2. Carbon dioxide sorption isotherms

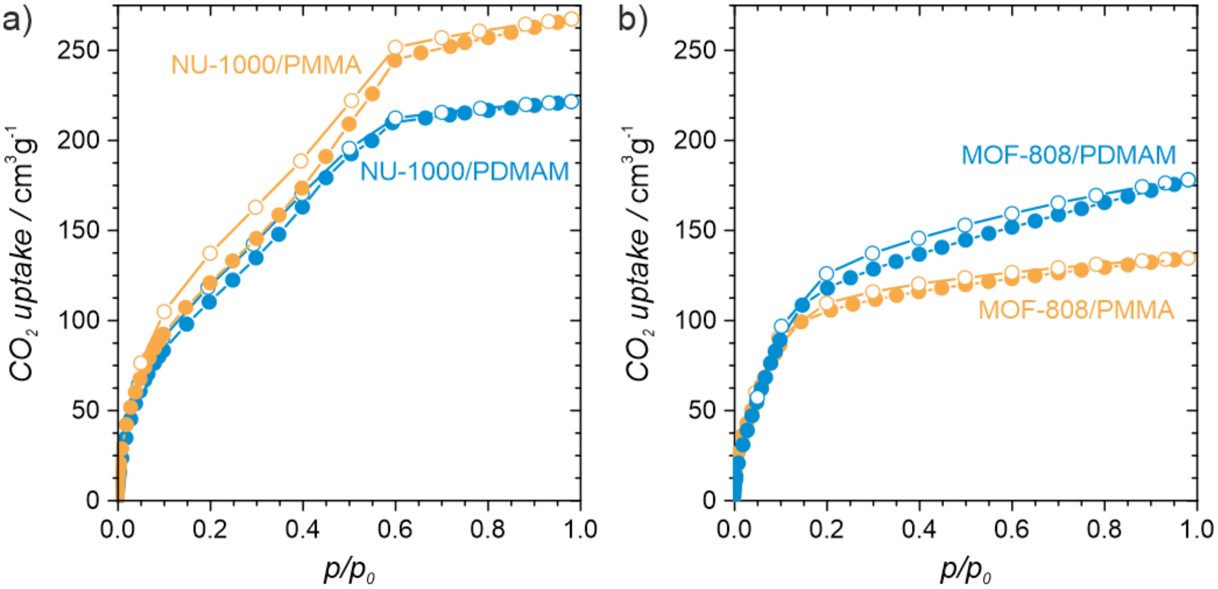

Figure S33. CO_2 sorption isotherms measured at 195 K for a) **NU-1000/polymer** and b) **MOF-808/polymer** hybrids (filled symbols - adsorption, open symbols - desorption).

Table S3. Total pore volumes of CO_2 (195 K) for **MOF/polymer** materials.

**sample:**	**NU-1000/PMMA**	**NU-1000/PDMAM**	**MOF-808/PMMA**	**MOF-808/PDMAM**
**V_{pore} (cm^3/g)[a]**	0.42	0.35	0.21	0.28

[a] Total pore volume calculated from a single-point adsorbate uptake at the relative pressure, p/p_0 = 0.98.

S8.3. Cyclohexane and water vapours sorption isotherms

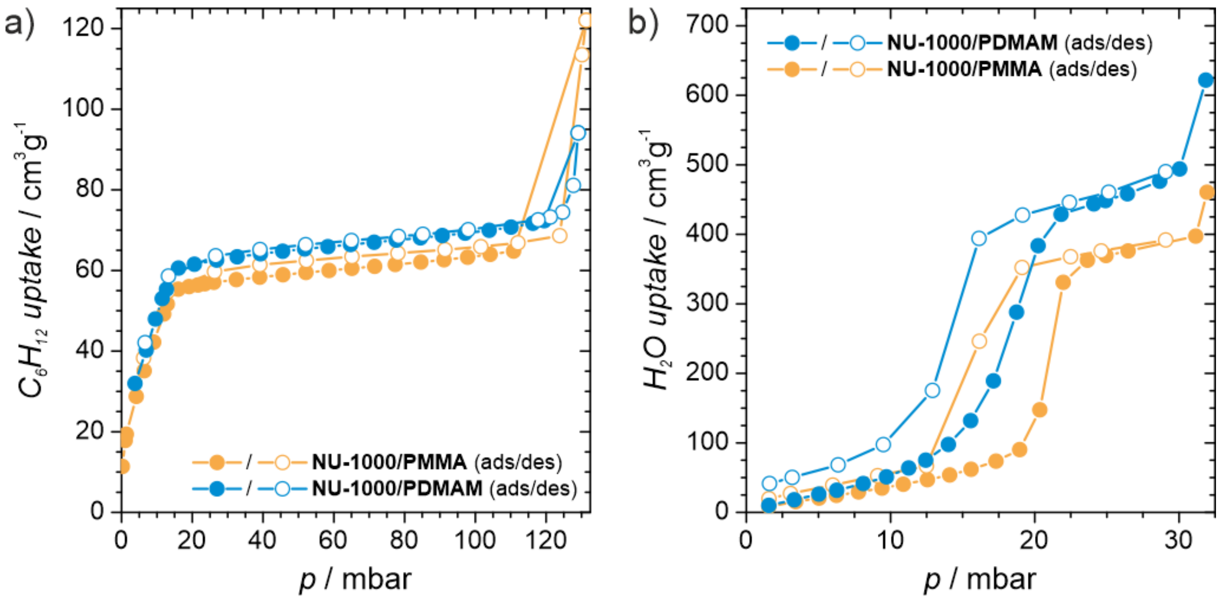

Figure S34. Sorption isotherms measured at 298 K for a) cyclohexane and b) water vapours for **NU-1000/polymer** hybrids; (filled symbols - adsorption, open symbols - desorption).

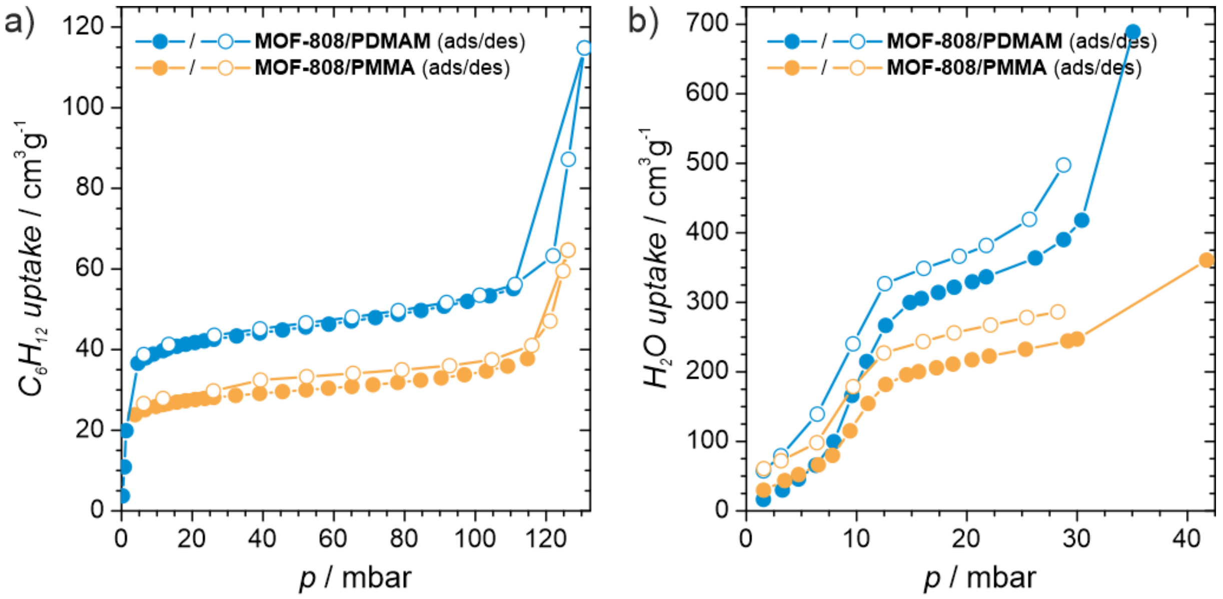

Figure S35. Sorption isotherms measured at 298 K for a) cyclohexane and b) water vapours for **MOF-808/polymer** hybrids; (filled symbols - adsorption, open symbols - desorption).

S10. SEM images and EDS analysis

S10.1. SEM images of initiator@MOF samples

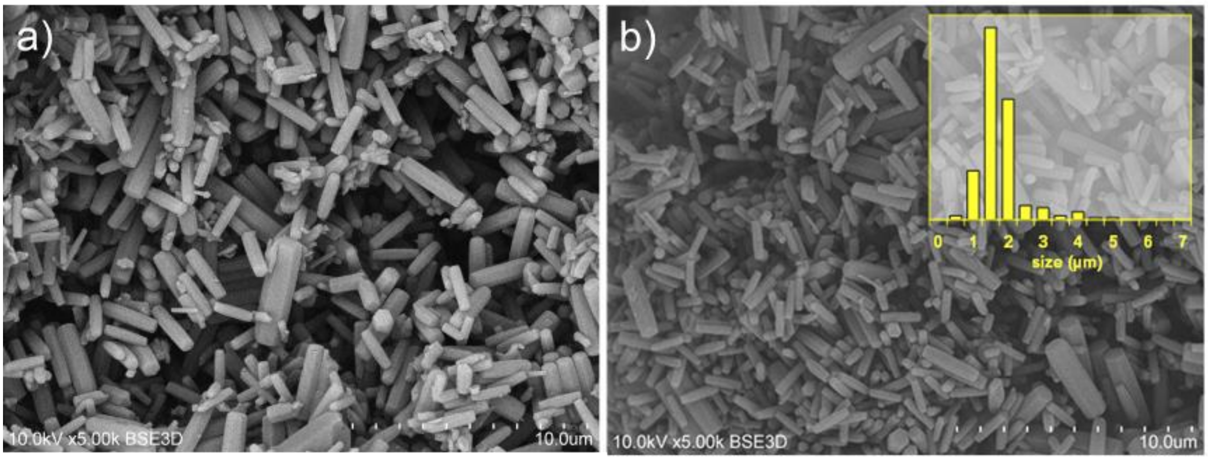

Figure S36. SEM images of a) **NU-1000** and b) **initiator@NU-1000** (crystal size distribution as an inset).

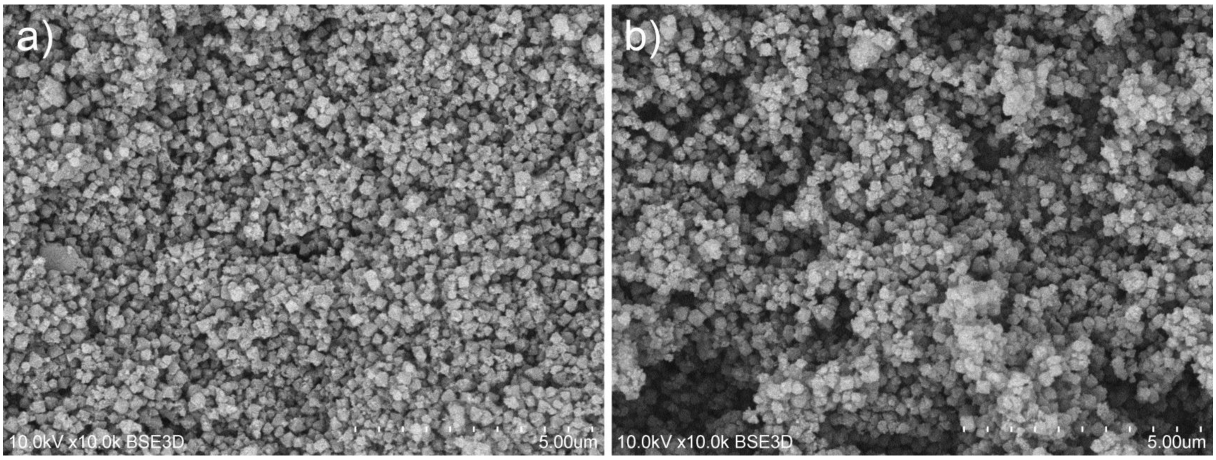

Figure S37. SEM images of a) **MOF-808** and b) **initiator@MOF-808**.

S10.2. SEM images of MOF/polymer materials

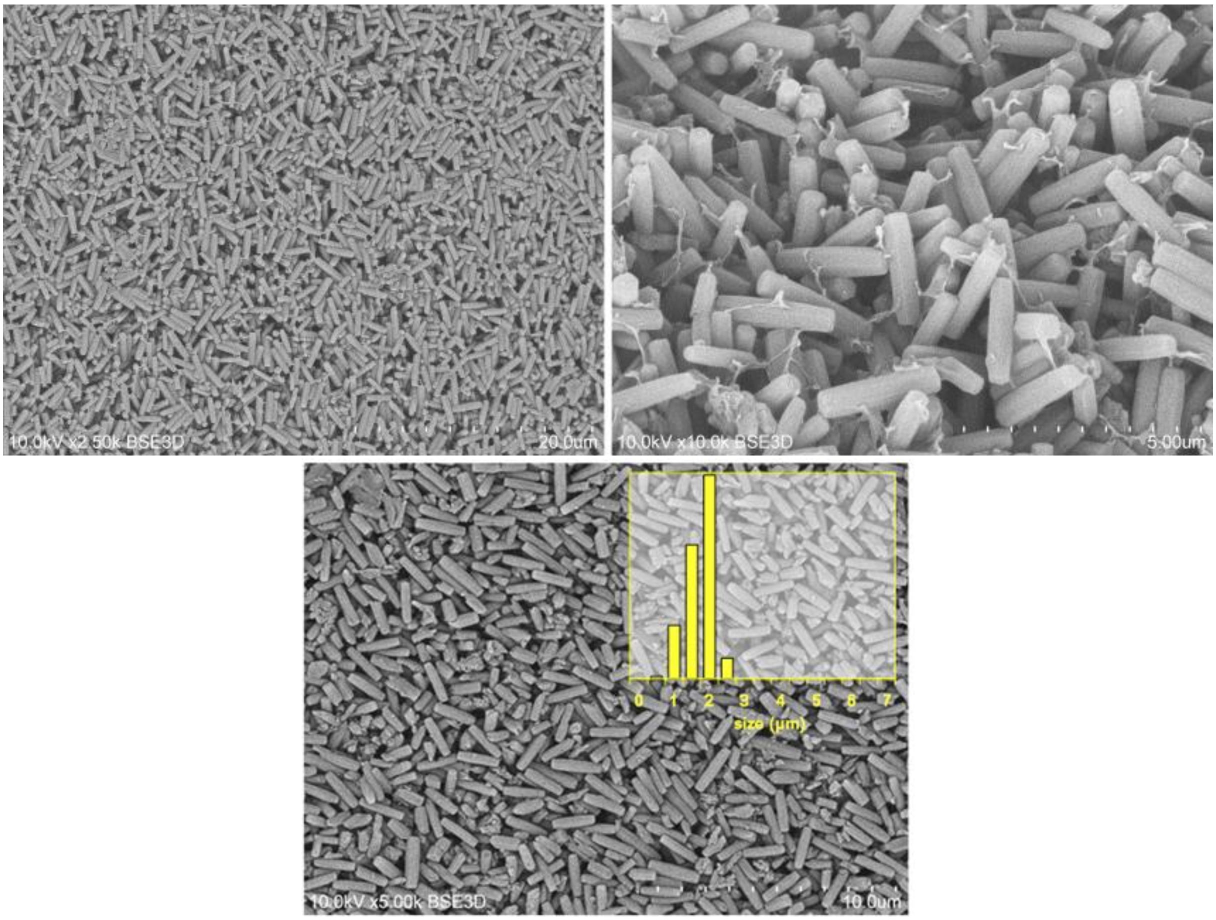

Figure S38. SEM images of the **NU-1000/PMMA** (particle size distribution as an inset).

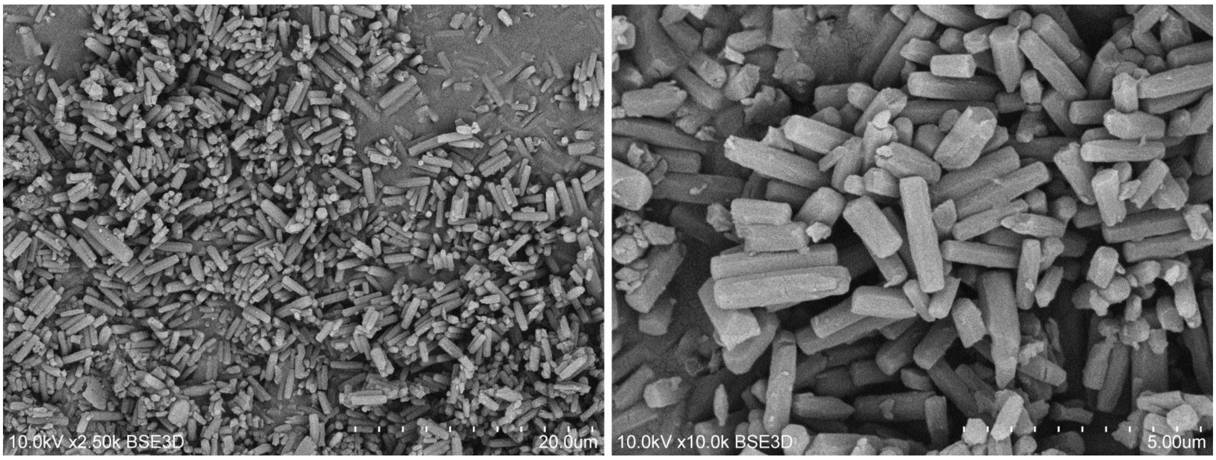

Figure S39. SEM images of the **NU-1000/PDMAM**.

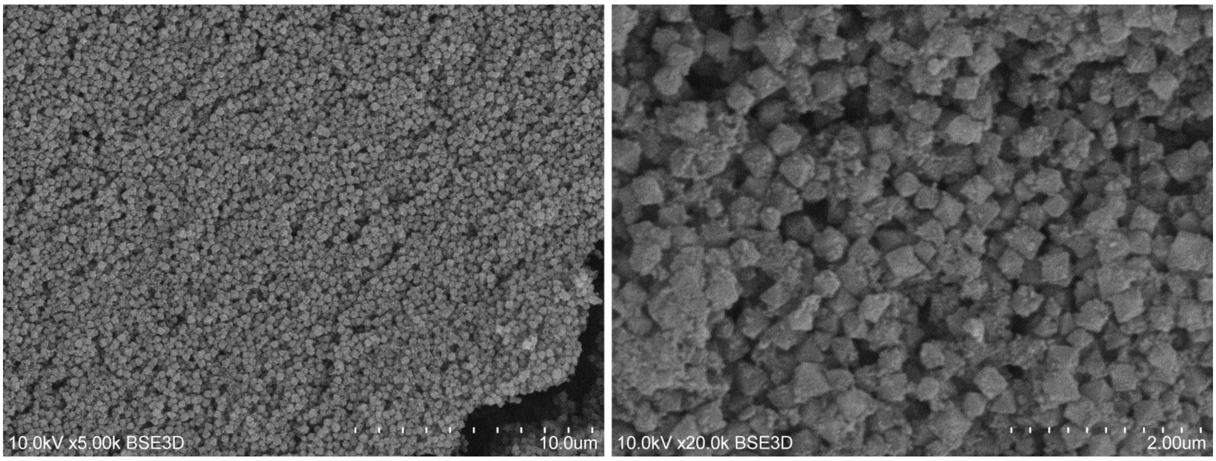

Figure S40. SEM images of the **MOF-808/PMMA**.

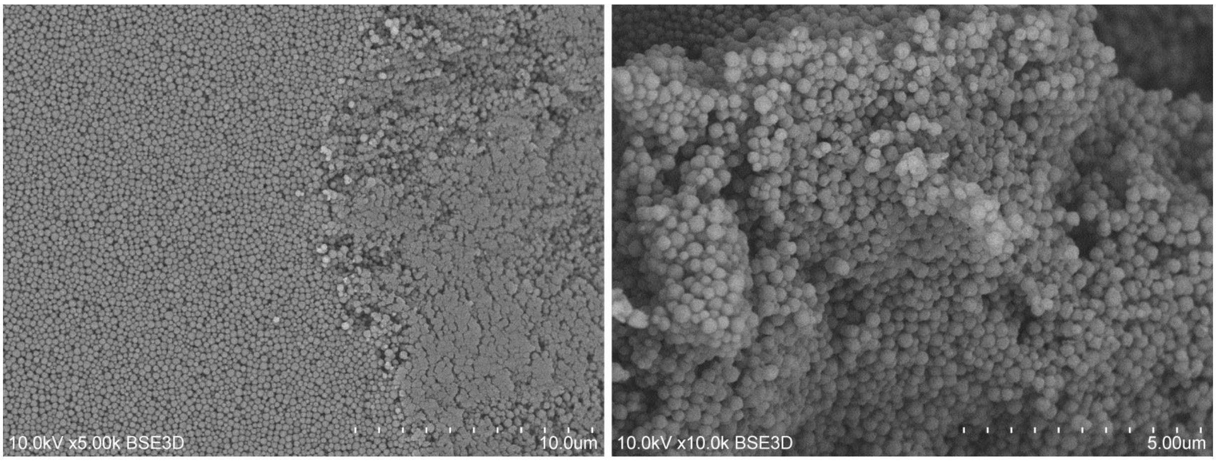

Figure S41. SEM images of the **MOF-808/PDMAM**.

S10.3. SEM images of MOF/polymer/fiber composites

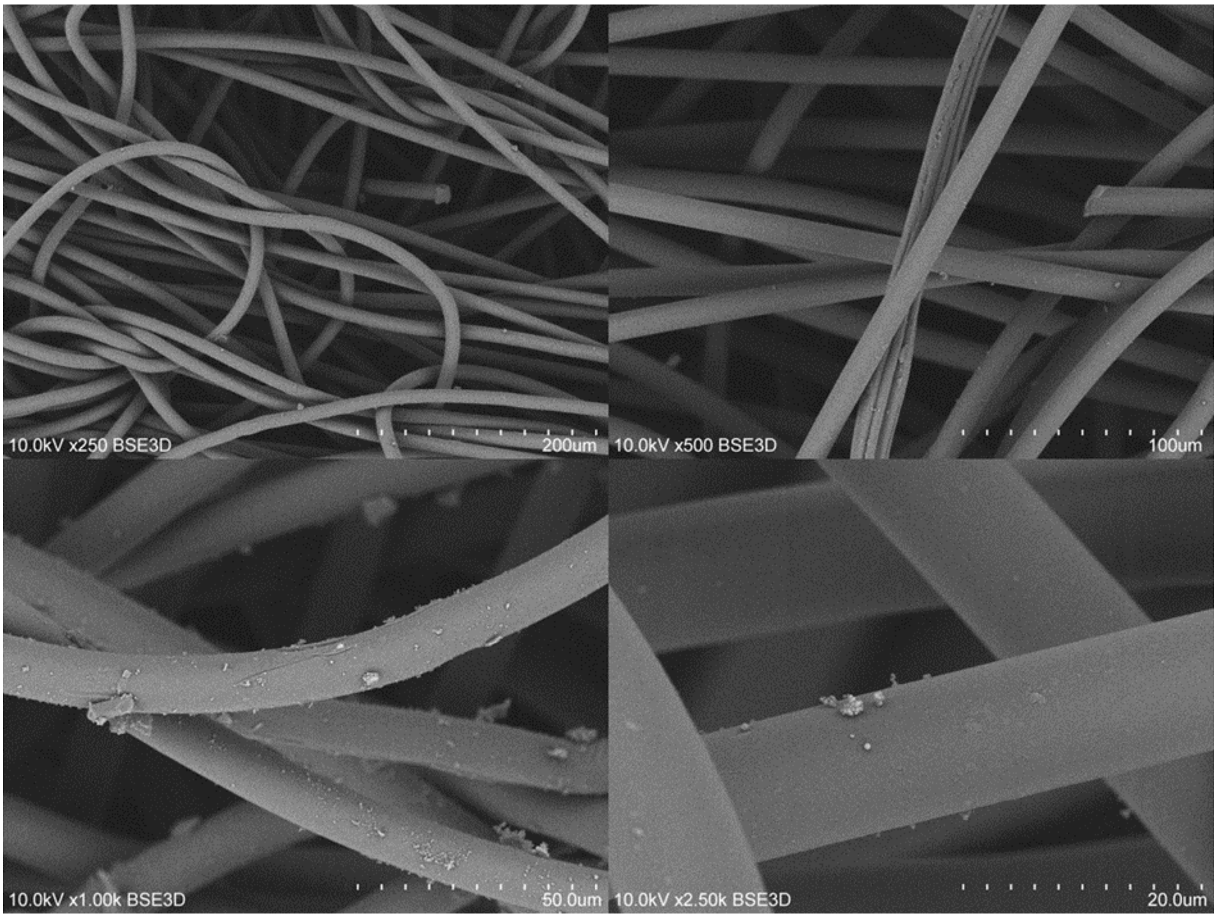

Figure S42. SEM images of the polypropylene (PP) fabric.

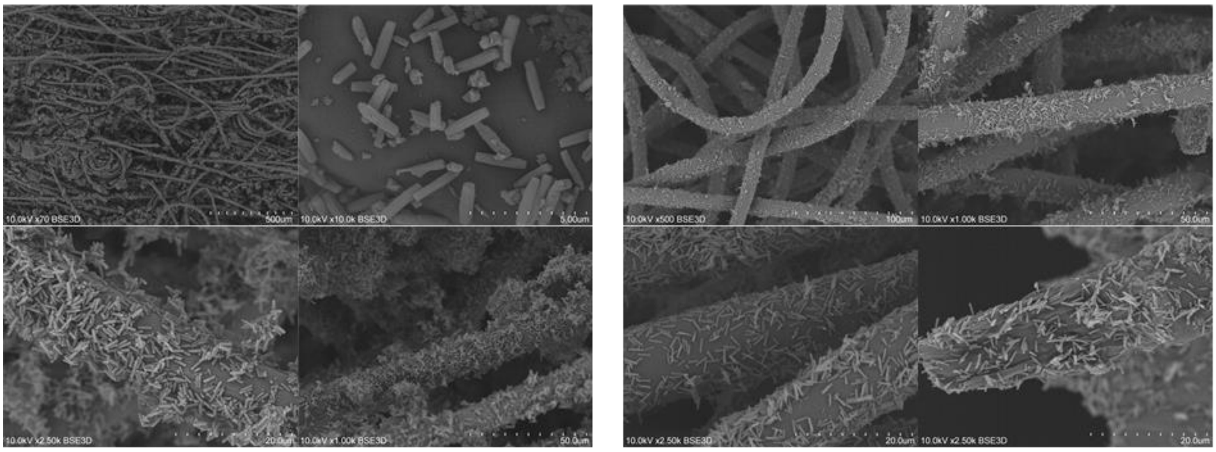

Figure S43. SEM images of the **NU-1000/PP** composite, prepared with the pristine **NU-1000**, before (left) and after (right) water treatment.

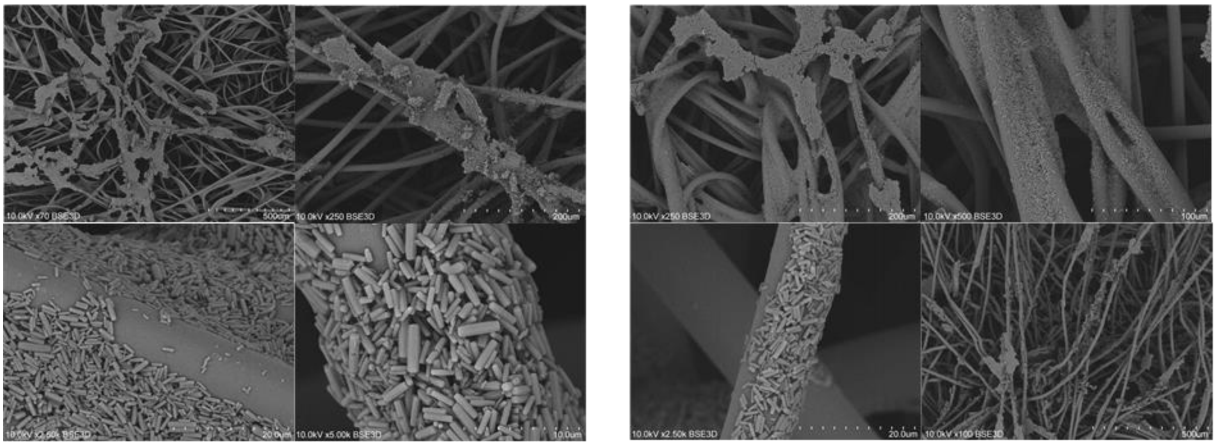

Figure S44. SEM images of the **NU-1000/PMMA/PP** composite before (left) and after (right) water treatment.

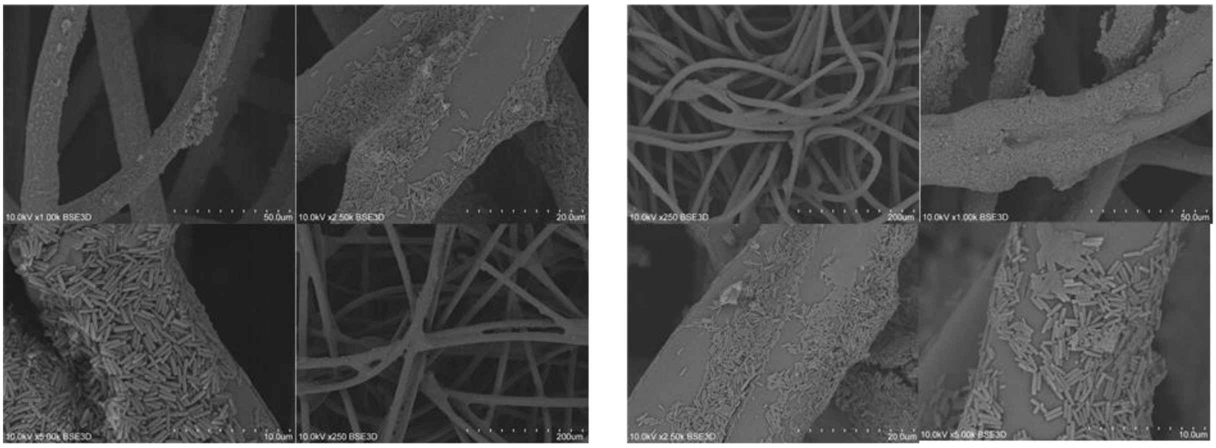

Figure S45. SEM images of the **NU-1000/PDMAM/PP** composite before (left) and after (right) water treatment.

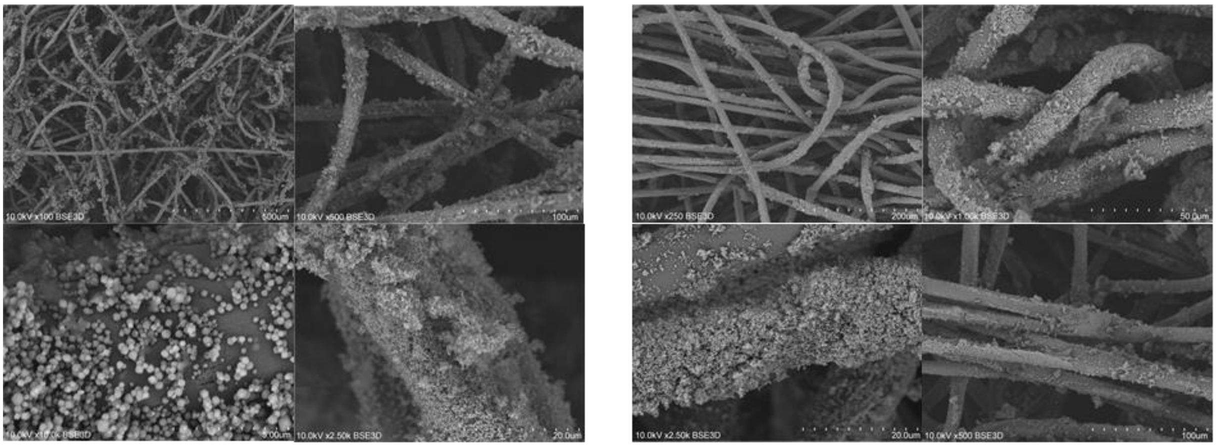

Figure S46. SEM images of the **MOF-808/PP** composite, prepared with the pristine **MOF-808**, before (left) and after (right) water treatment.

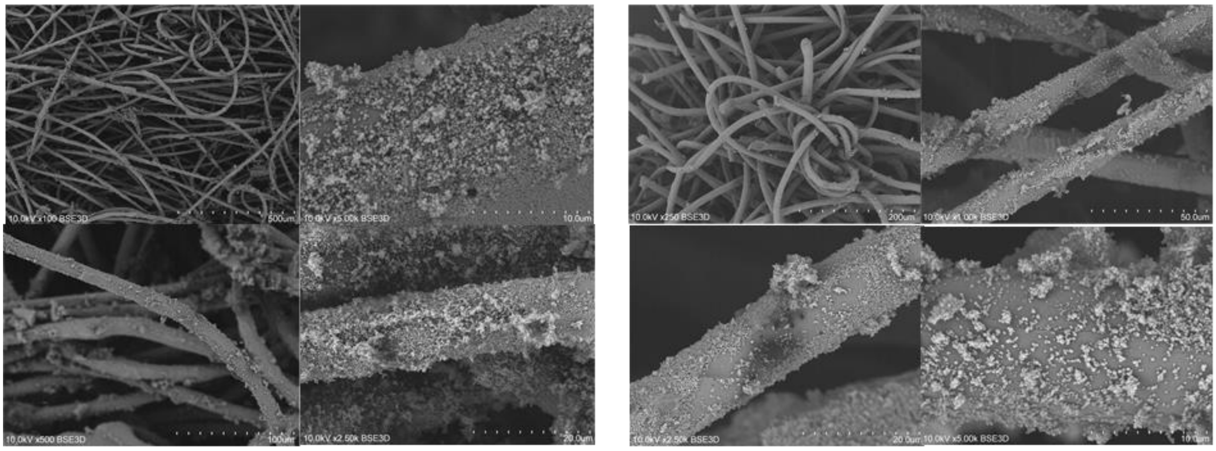

Figure S47. SEM images of the **MOF-808/PMMA/PP** composite before (left) and after (right) water treatment.

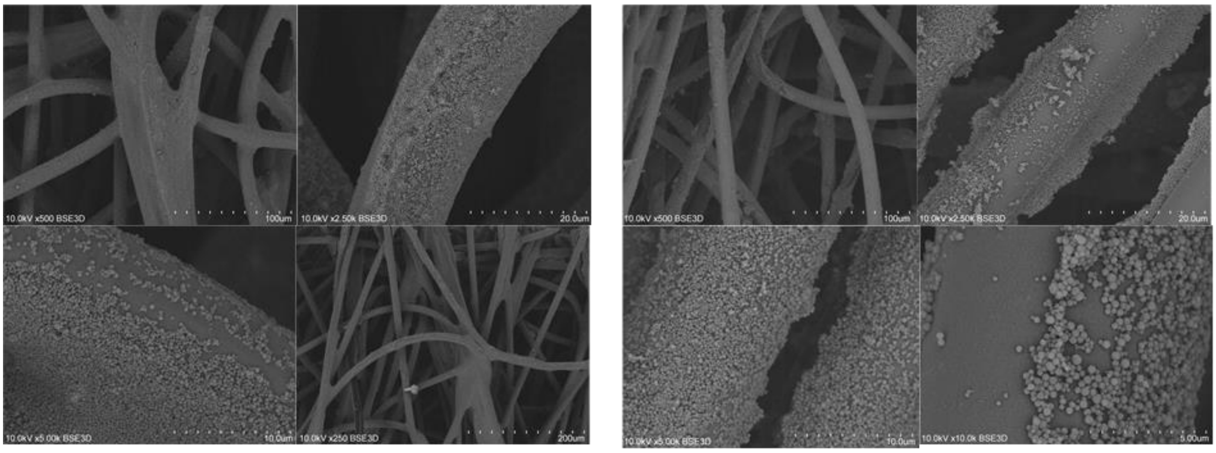

Figure S48. SEM images of the **MOF-808/PDMAM/PP** composite before (left) and after (right) water treatment.

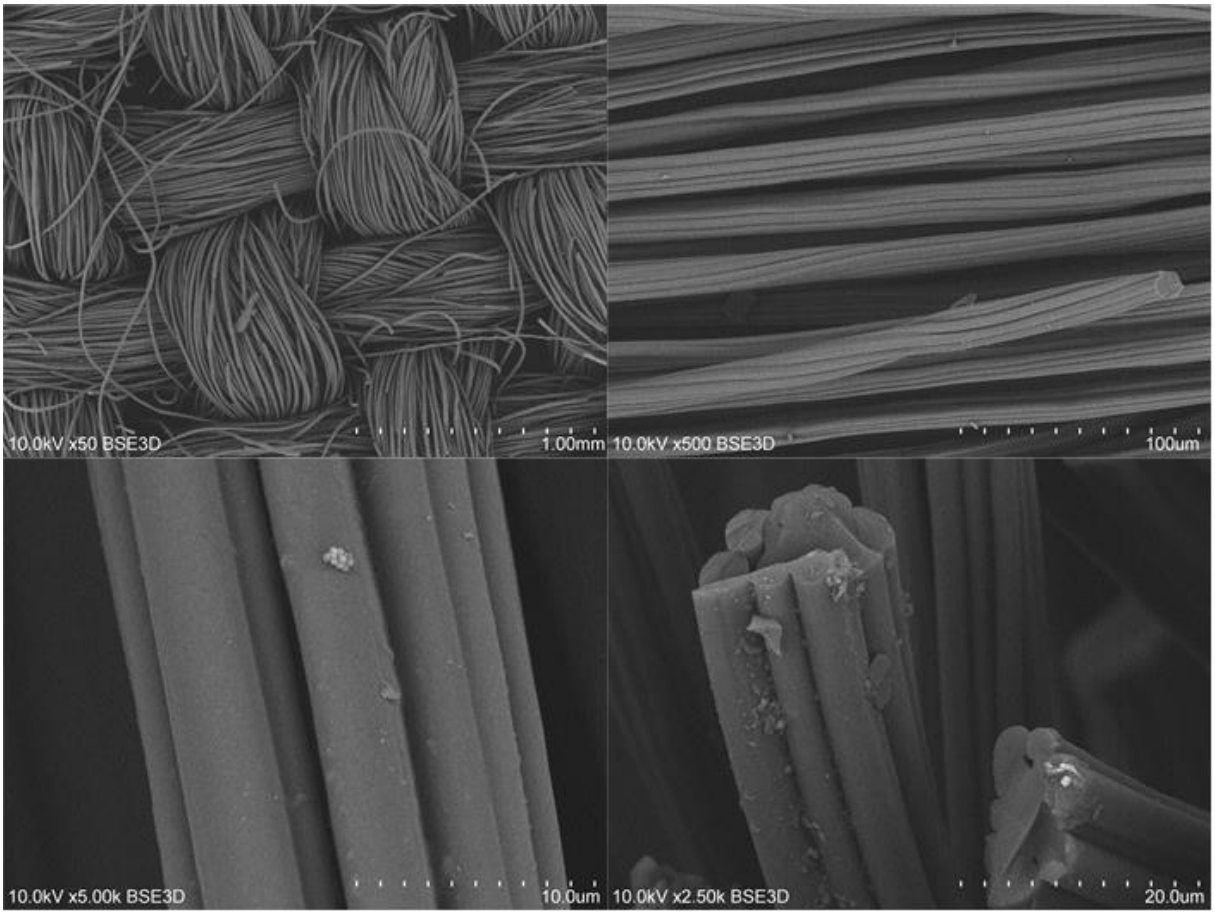

Figure S49. SEM images of the pristine activated carbon (AC) fabric.

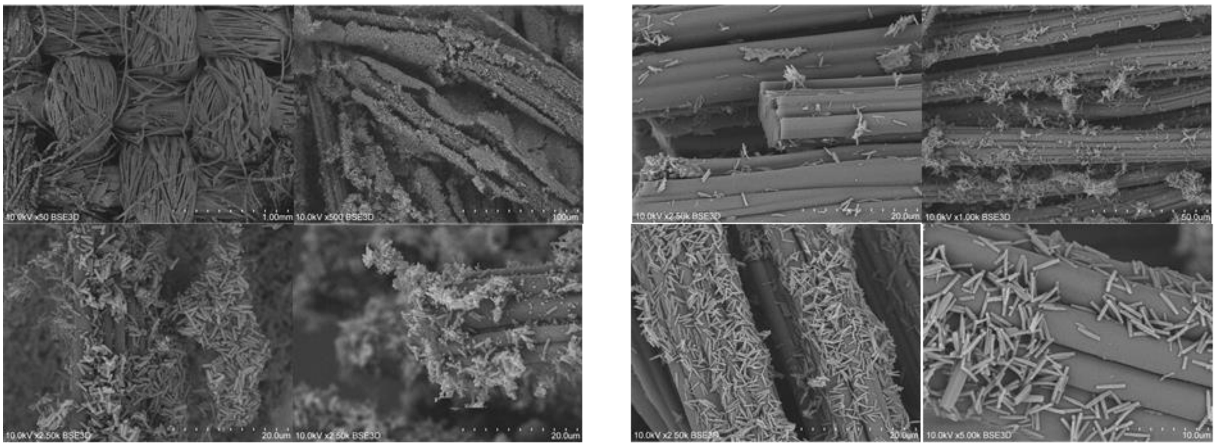

Figure S50. SEM images of the **NU-1000/AC** composite, prepared with the pristine **NU-1000**, before (left) and after (right) water treatment.

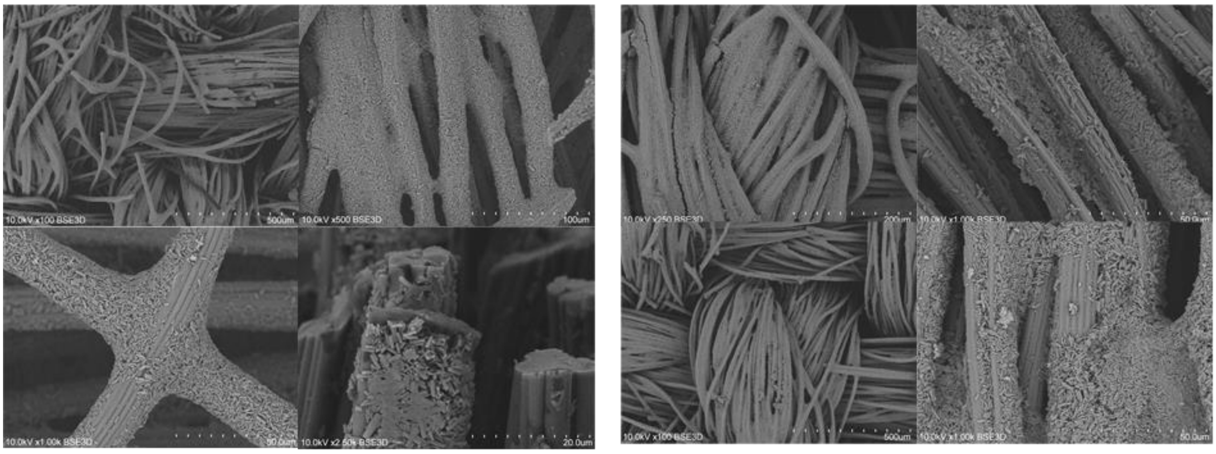

Figure S51. SEM images of the **NU-1000/PMMA/AC** composite before (left) and after (right) water treatment.

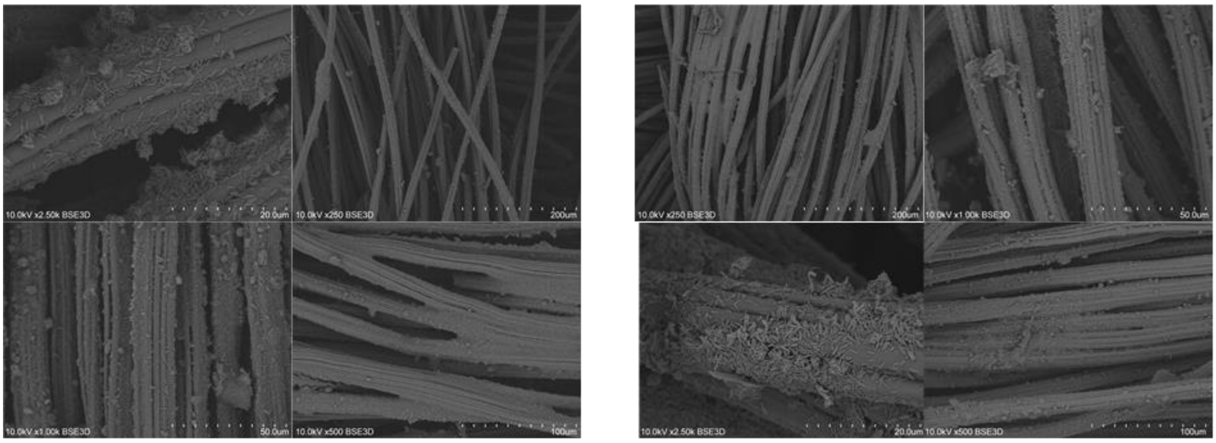

Figure S52. SEM images of the **NU-1000/PDMAM/AC** composite before (left) and after (right) water treatment.

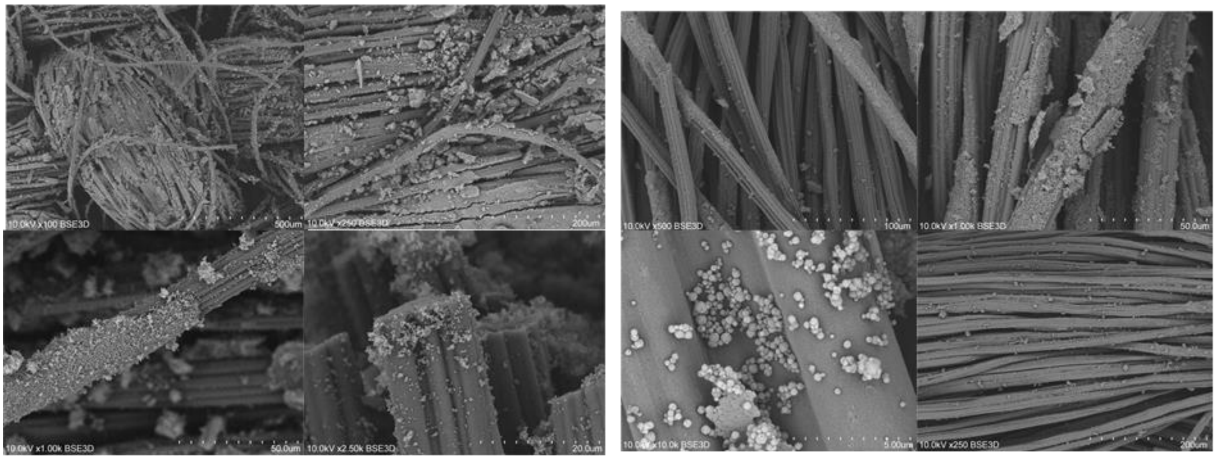

Figure S53. SEM images of the **MOF-808/AC** composite, prepared with the pristine **MOF-808**, before (left) and after (right) water treatment.

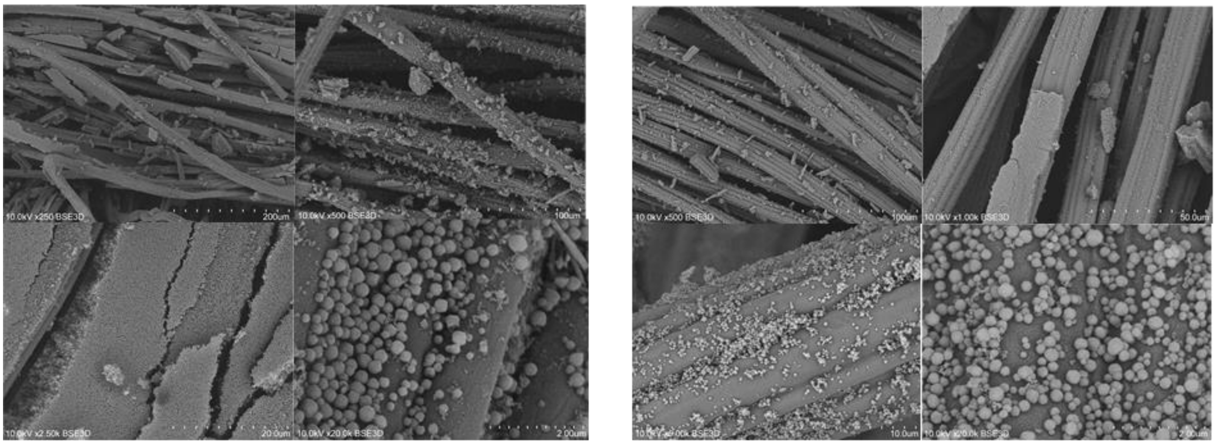

Figure S54. SEM images of the **MOF-808/PMMA/AC** composite before (left) and after (right) water treatment.

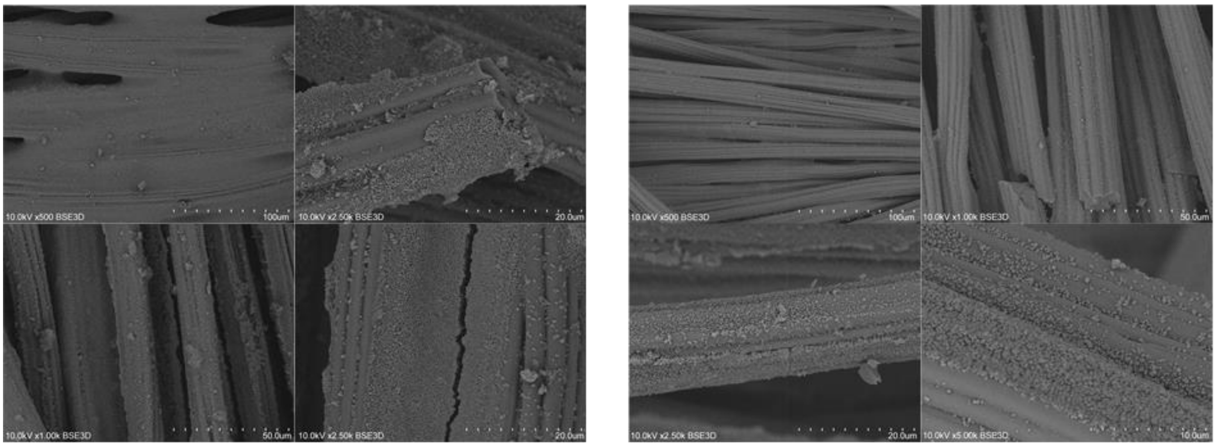

Figure S55. SEM images of the **MOF-808/PDMAM/AC** composite before (left) and after (right) water treatment.

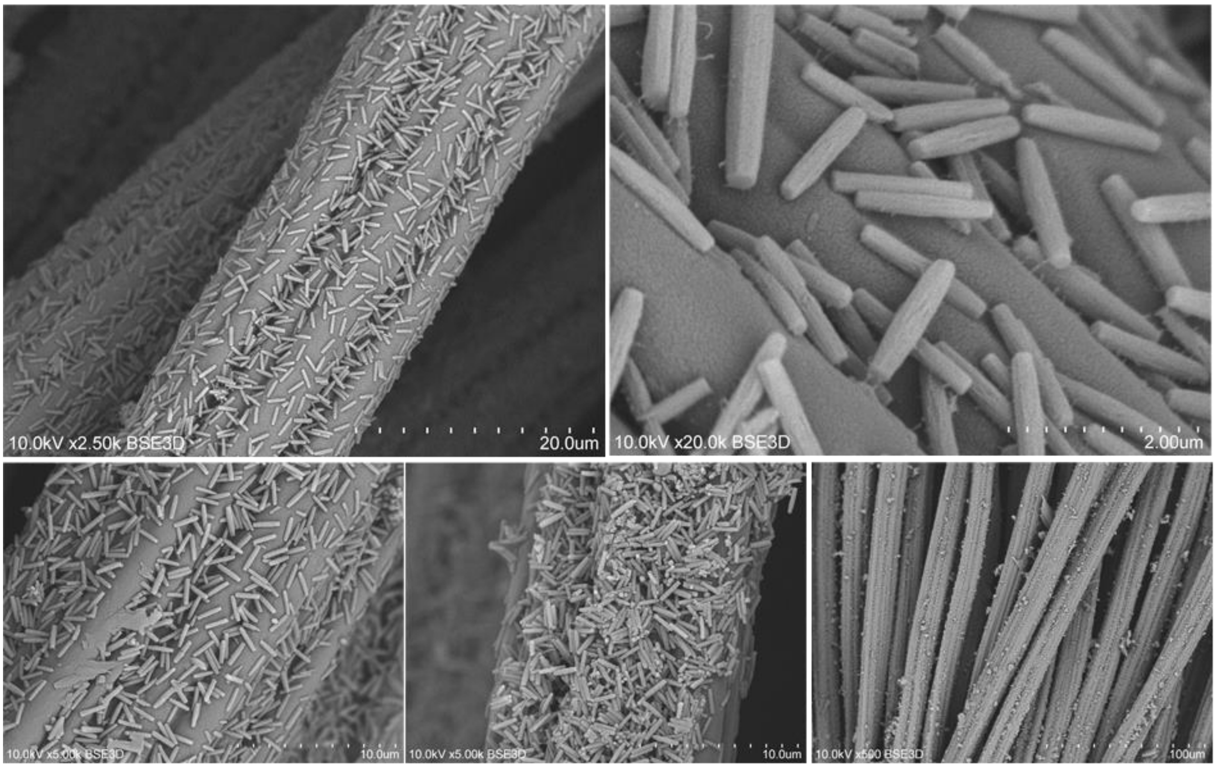

Figure S56. SEM images of the **NU-1000/PDMAM/AC** composite prepared using the dispersion of **NU-1000/PDMAM** hybrid in water.

S11. TGA-DTG analysis

Thermogravimetric analysis (TGA) was conducted in the range of 30-1000 °C under oxidizing conditions (O_2/N_2 = 20/80) with a heating rate of 5 °C/min, giving as the only final product zirconium oxide (ZrO_2)[9]. The weight loss at the beginning of all of the TGA curves corresponds to the removal of solvent molecules (up to ca. 200 °C). The decomposition of the parent MOF occurs in the temperature range of 400-500 °C. The TGA curves presented on **Figures S57-S59** were normalized in respect to the residual ZrO_2 mass (100%)[9].

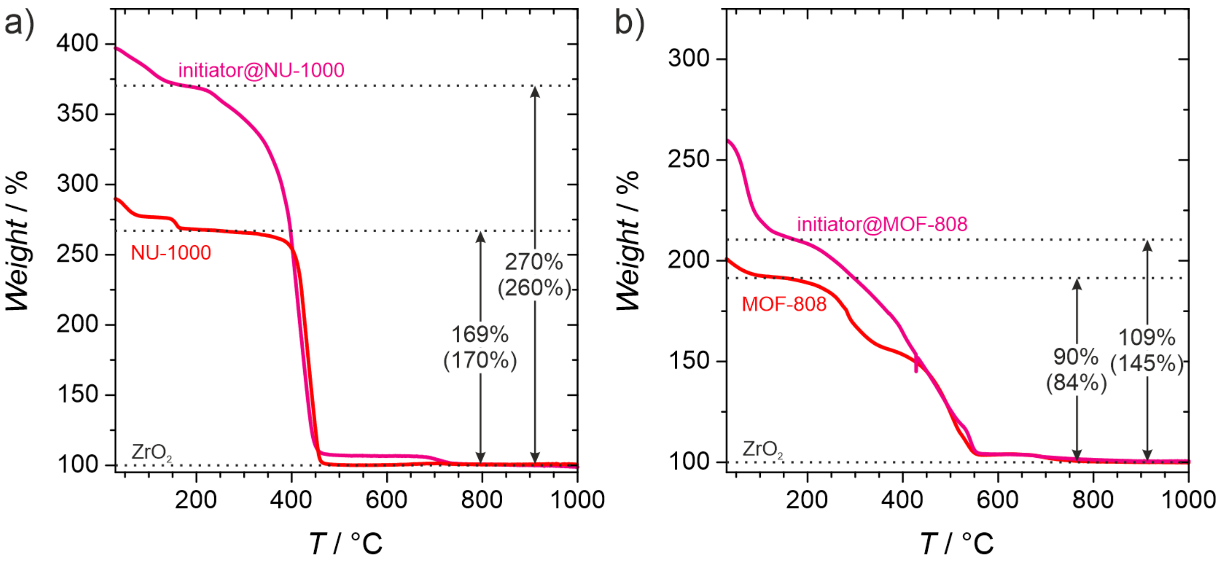

Figure S57. TGA profiles of a) **NU-1000** (red line) and **initiator@NU-1000** (pink line) and b) **MOF-808** (red line) and **initiator@MOF-808** (pink line). The values in the parentheses are calculated theoretical weight losses based on the chemical formula of selected MOFs (see Table **S4**).

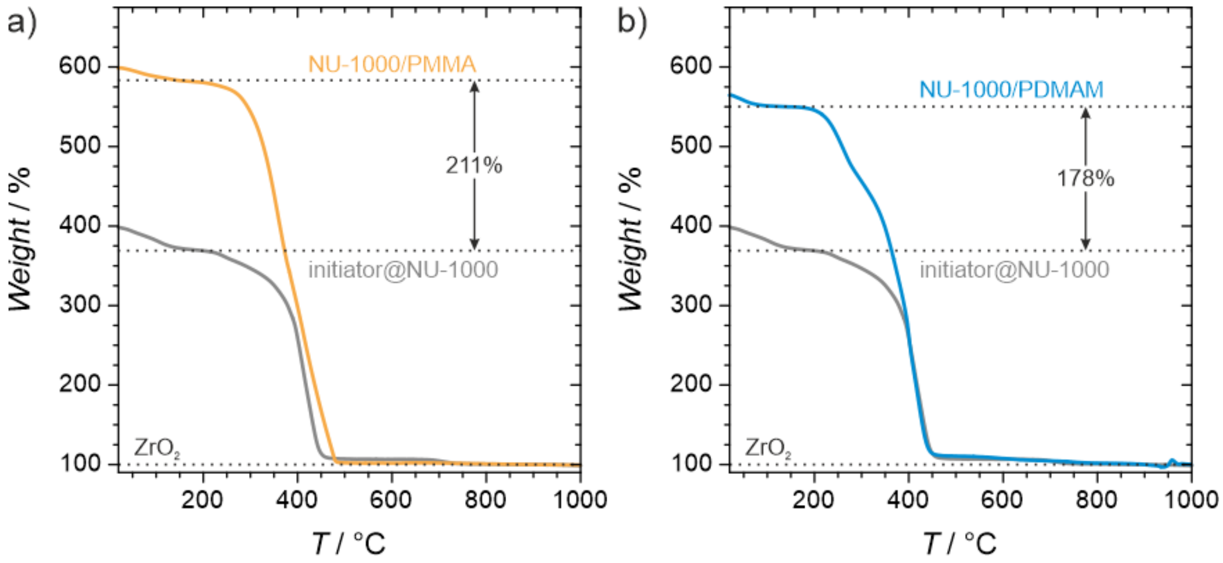

Figure S58. TGA profiles of a) **NU-1000/PMMA** (orange line) and b) **NU-1000/PDMAM** (blue line). For comparison the TGA curve measured for **initiator@NU-1000** material is included (grey line). The mass increase of **MOF/polymer** material in respect to the **initiator@MOF** material is annotated with the arrow.

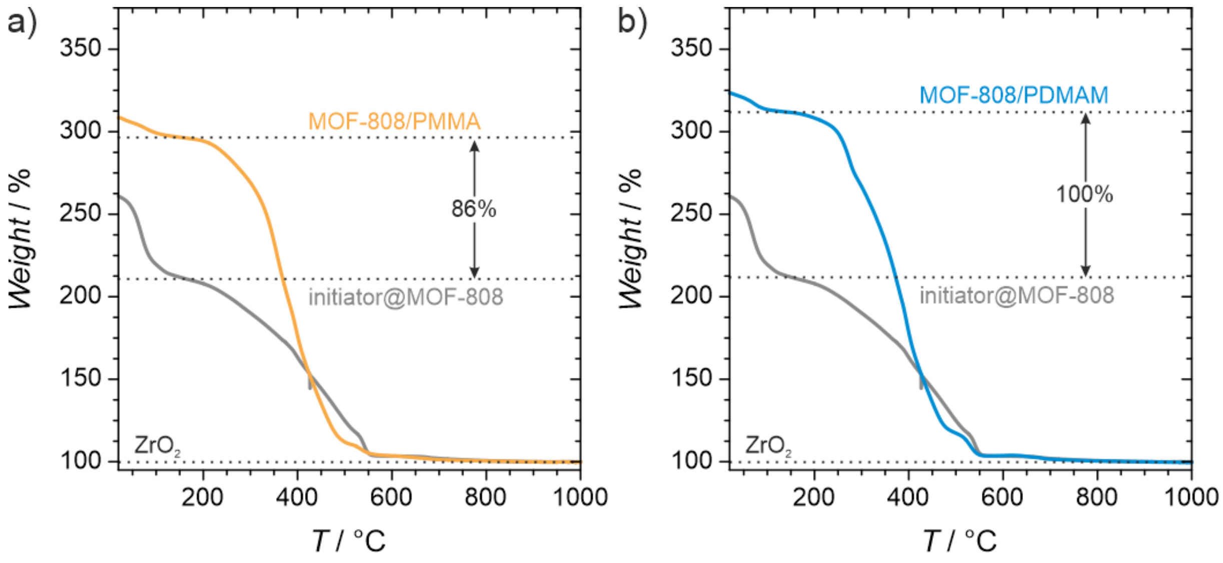

Figure S59. TGA profiles of a) **MOF-808/PMMA** (orange line) and b) **MOF-808/PDMAM** (blue line). For comparison the TGA curve measured for **initiator@MOF-808** material is included (grey line). The mass increase of **MOF/polymer** material in respect to the **initiator@MOF** material is annotated with the arrow.

S12. Composition of MOF/polymer hybrids from TGA and ICP-OES

The quantity of Zr (wt%) in the obtained Zr-based MOF materials and **MOF/polymer** hybrids was calculated from TGA (see section S11). The Zr content (wt%) in obtained materials was calculated using equation (1):

$$\text{Zr (wt\%)} = \frac{m_{900\,°C}}{m_{190\,°C}} \times 100\% \times \frac{M_{Zr}}{M_{ZrO_2}} = \frac{m_{900\,°C}}{m_{190\,°C}} \times 74\% \qquad (1)$$

where:

$m_{900\,°C}$ - mass from TGA analysis at 900 °C (corresponds to ZrO_2);

$m_{190\,°C}$ - mass from TGA analysis at 190 °C (corresponds to solvent-free material);

M_{Zr} - molar mass of Zr (91.22 g/mol);

M_{ZrO_2} - molar mass of ZrO_2 (123.22 g/mol).

The calculated values are collected in Table S4 and are in good agreement with theoretical values. The Zr content measured by ICP-OES (Table S4) is typically lower, due to the presence of solvent trapped in the pores of the MOF materials.

Table S4. The calculated and theoretical Zr content for obtained MOFs and **initiator@MOF** materials.

**sample**	**chemical formula**[1]	**Zr (wt%)**		
		**theoretical**	**exp (TGA)**[2]	**exp (ICP)**[3]
**NU-1000**	$Zr_6O_6(TBAPy^{4-})_2$	27.4	27.8	21.2
**initiator@NU-1000**	$Zr_6O_6(TBAPy^{4-})_2(ACPA)_{2.5}$	20.8	20.0	17.2
**MOF-808**	$Zr_6O_6(BTC^{3-})_2(FA^{-})_6$	41.2	39.0	37.3
**initiator@MOF-808**	$Zr_6O_6(BTC^{3-})_2(ACPA)_3$	30.3	35.5	25.6

[1] theoretical composition of the solvent-free, dehydroxylated MOF; the amount of ACPA in **initiator@MOF** was determined with NMR

[2] based on TGA using equation (1)

[3] based on ICP-OES

The amount of polymer (wt%) in **MOF/polymer** hybrids was estimated from the normalized TGA curves (see section S11). The weight difference (%) between **initiator@MOF** and **MOF/polymer** material at 190 °C (Figures **S58**-**S59**) corresponds to the weight of the polymer. The amount of polymer (wt%) was calculated using equation (2):

$$\text{polymer (wt\%)} = \frac{W_{MOF/polymer} - W_{initiator@MOF}}{W_{MOF/polymer}} \times 100\% \qquad (2)$$

where:

$W_{MOF/polymer}$ - weight (%) of **MOF/polymer** composite at 190 °C from TGA;

$W_{initiator@MOF}$ - weight (%) of **initiator@MOF** material at 190 °C from TGA.

Table S5. The calculated composition and Zr content for **MOF/polymer** hybrids.

**sample**	**Zr (wt%)**[1]	**Zr (wt%)**[2]	**polymer (wt%)**[3]	**monomer/Zr_6-node**[4]	**monomer/Zr_6-node**[5]
**NU-1000/PMMA**	12.7	9.9	36	15.5	1013
**NU-1000/PDMAM**	13.5	9.6	33	13.4	800
**MOF-808/PMMA**	25.1	20.3	29	4.0	707
**MOF-808/PDMAM**	23.9	20.6	32	4.7	558

[1]based on TGA using equation (1); [2]based on ICP-OES; [3]based on TGA using equation (2); [4]molar ratio of the estimated amount of monomer in the corresponding polymer to 6 Zr in MOF/polymer hybrid; [5]starting ratio of monomer to initiator@MOF for *FRaP-in-MOF*.

S13. Reproducibility tests of the *FRaP-in-MOF* protocol

To confirm the reproducibility of the *FRaP-in-MOF* procedure, four batches of **NU-1000/PMMA** were prepared and characterized with SEM (Fig. S60) and N_2 sorption studies (Fig. S61). Additionally, the TGA (Fig. S62) and ICP-OES measurements were made for quantitative analysis, and the calculated **NU-1000/PMMA** composition is collected in Table S6.

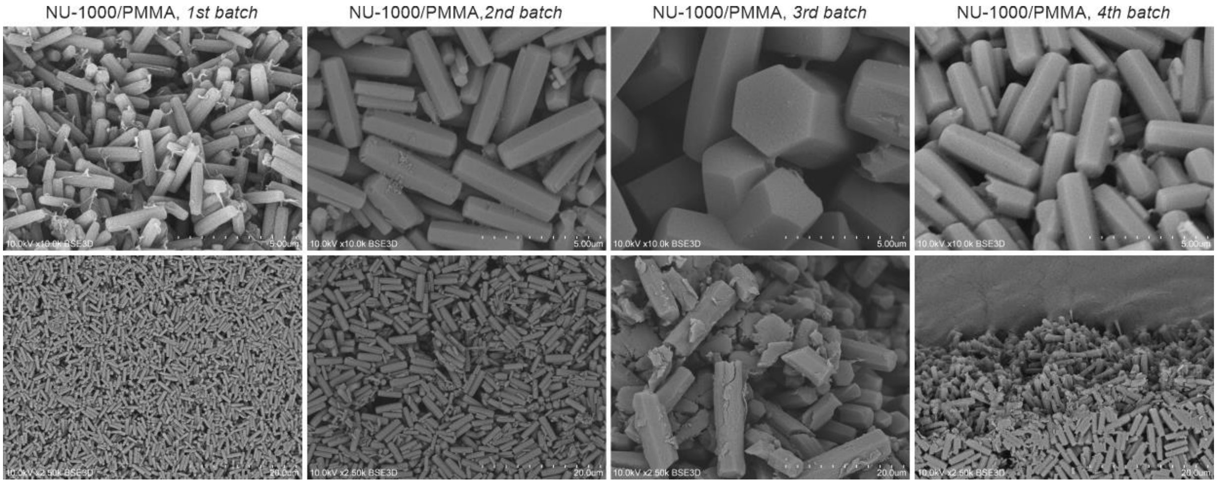

Figure S60. SEM images of prepared batches of **NU-1000/PMMA**.

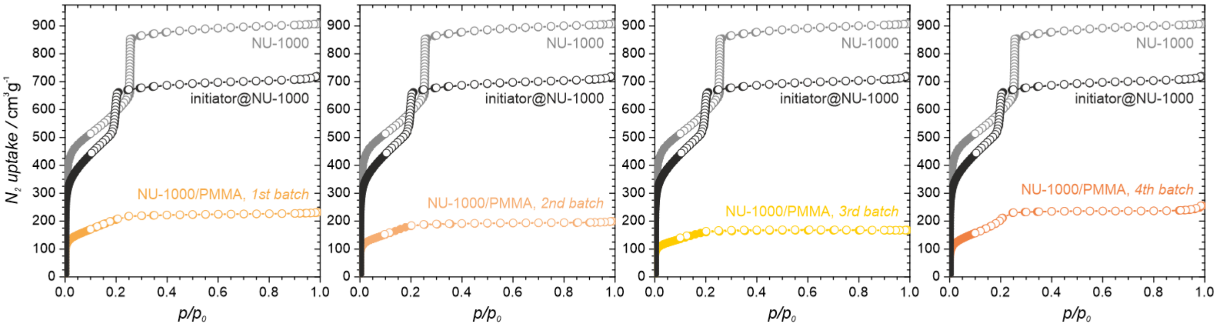

Figure S61. N_2 sorption isotherms measured at 77 K for separate batches of **NU-1000/PMMA**. The isotherms of **NU-1000** and **initiator@NU-1000** are shown for comparison (filled symbols - adsorption, open symbols - desorption).

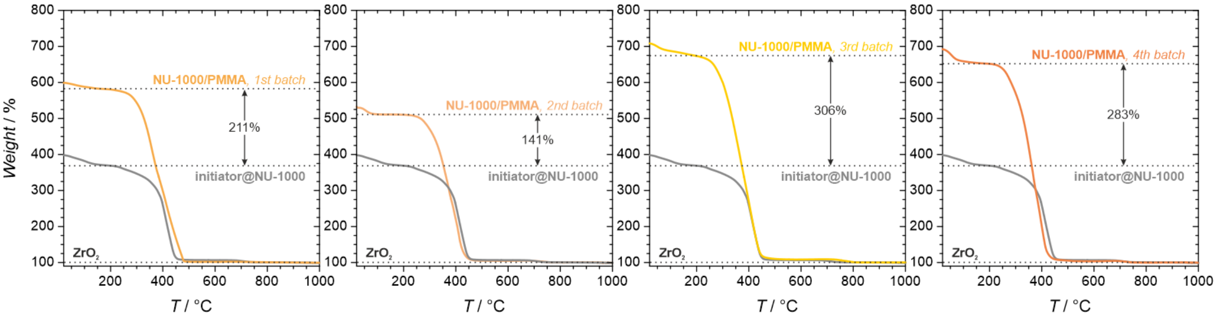

Figure S62. TGA profiles of separate batches of **NU-1000/PMMA** compared with TGA curve measured for **initiator@NU-1000** (grey line). The mass increase of **MOF/polymer** material in respect to the **initiator@MOF** material is annotated with an arrow.

Table S6. The sorption data, calculated composition and Zr content for prepared batches of **NU-1000/PMMA** materials.

NU-1000/PMMA sample	Zr (wt%)[1]	Zr (wt%)[2]	polymer (wt%)[3]
**1st batch**	12.7	9.9	36
**2nd batch**	14.5	10.8	28
**3rd batch**	11.0	10.3	45
**4th batch**	11.3	8.0	43

[1] based on TGA analysis, following the equation (1)
[2] based on ICP-OES measurement
[3] based on TGA analysis, following the equation (2)

S14. Preparation of MOF/polymer hybrids - control experiments

For control experiments 1 ml of respective monomers (9.4 mmol of MMA, 5.9 mmol of DMAM) were stirred at 70 °C (for MMA) or 50 °C (for DMAM) for 48 hours. No visible changes of the reaction mixture were observed after that time, and the remained clear solution was analyzed with ^{1}H NMR analysis confirming presence of only unreacted monomers (Fig. S63, S65).
In the same reaction conditions, 2 ml of MMA (18.8 mmol) or DMAM (11.9 mmol) were stirred in the presence of 50 mg of pristine **NU-1000** or **MOF-808**. The collected ^{1}H NMR spectra (Fig. S64 and S66) of the supernatant of the reaction mixture confirm Zr-MOF inactivity in the polymerization reaction.

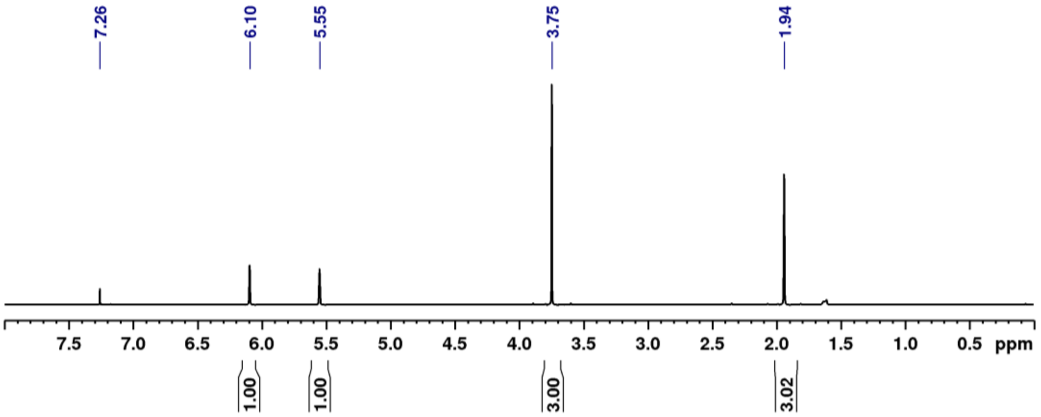

Figure S63. ^{1}H NMR spectrum of reaction mixture after incubating MMA monomer at 70 °C for 48 h.

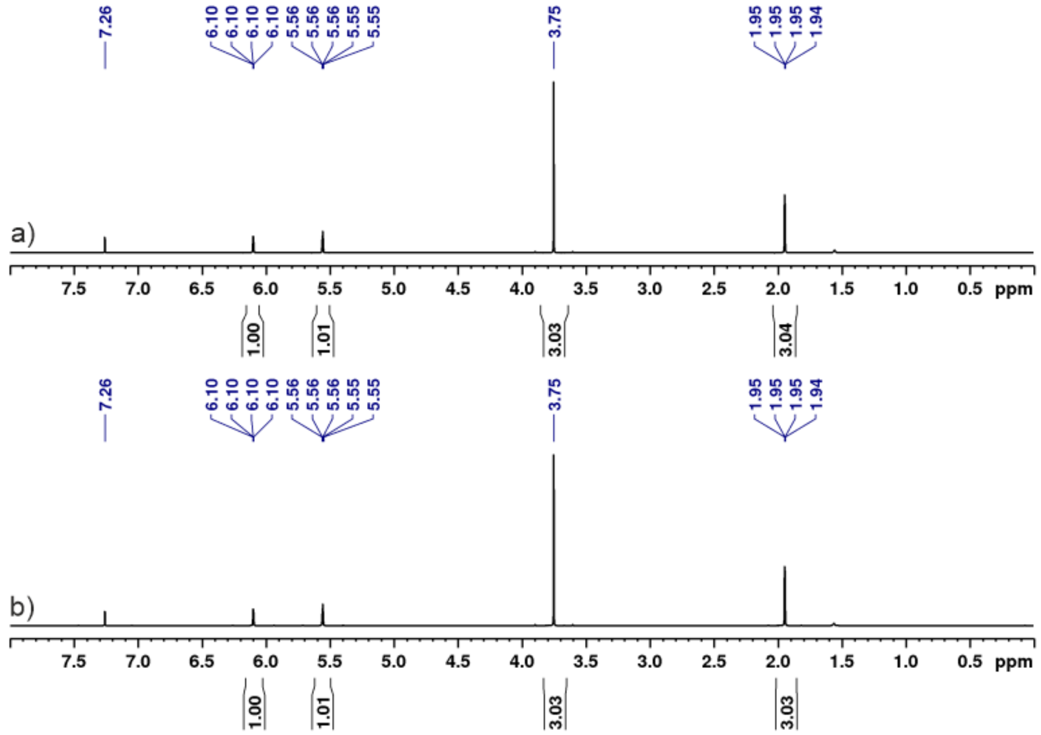

Figure S64. ^{1}H NMR spectrum of the supernatant of the reaction mixture of MMA monomer at 70 °C for 48 h in the presence of pristine **NU-1000** (a) and **MOF-808** (b).

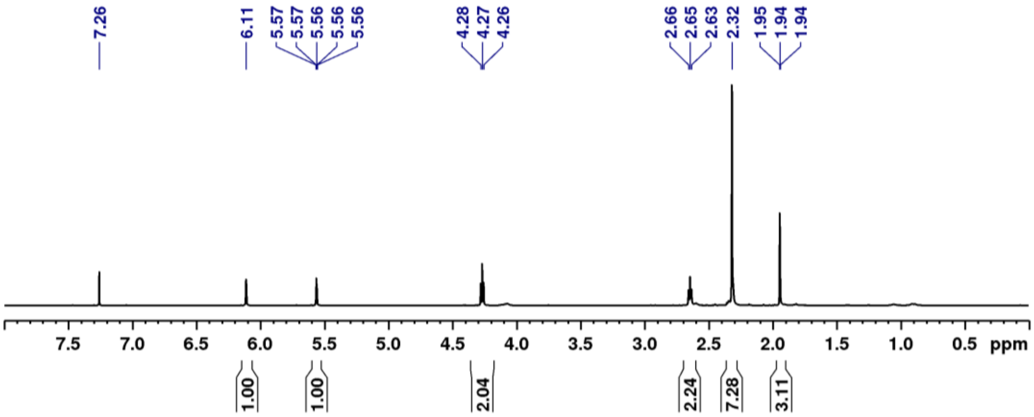

Figure S65. ^{1}H NMR spectrum of reaction mixture after incubating DMAM monomer at 50 °C for 48 h.

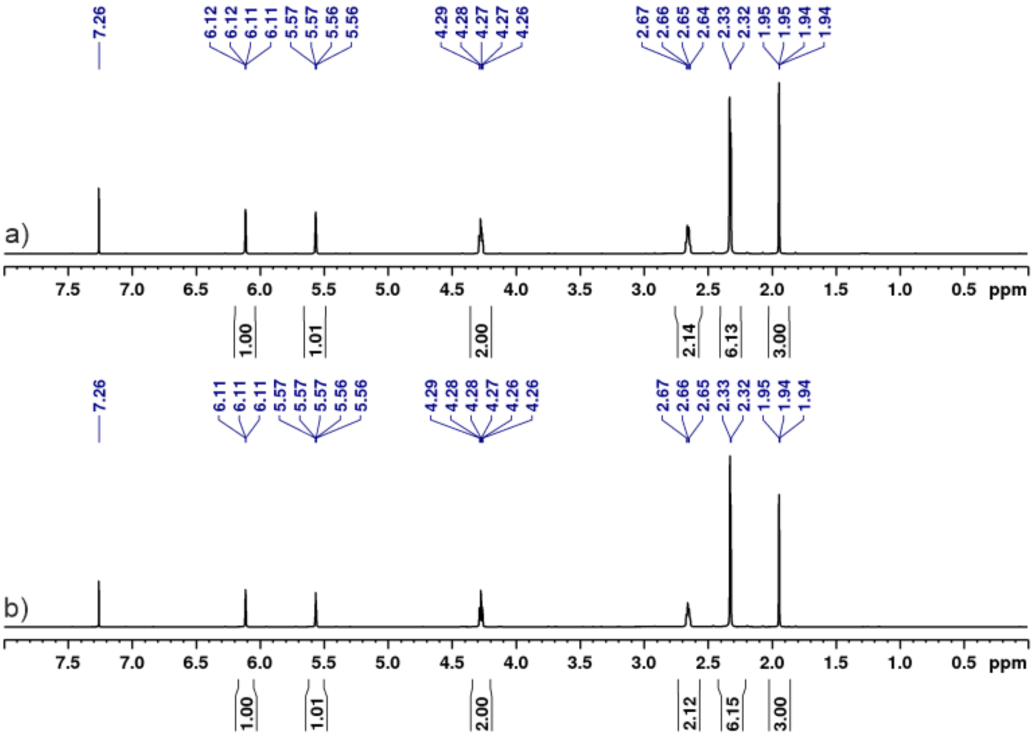

Figure S66. ^{1}H NMR spectrum of the collected supernatant of the reaction mixture of DMAM monomer and pristine **NU-1000** at 50 °C for 48 h.

The reference sample of **NU-1000/PMMA*** before washing procedure with acetone (removal of excess of PMMA polymer chains) was analyzed with TGA and SEM analysis (Fig. S67). The determined composition of **NU-1000/PMMA*** sample (calculations were made following the equations (1) and (2)) was 2.9 wt% of Zr and 86 wt% of PMMA polymer.

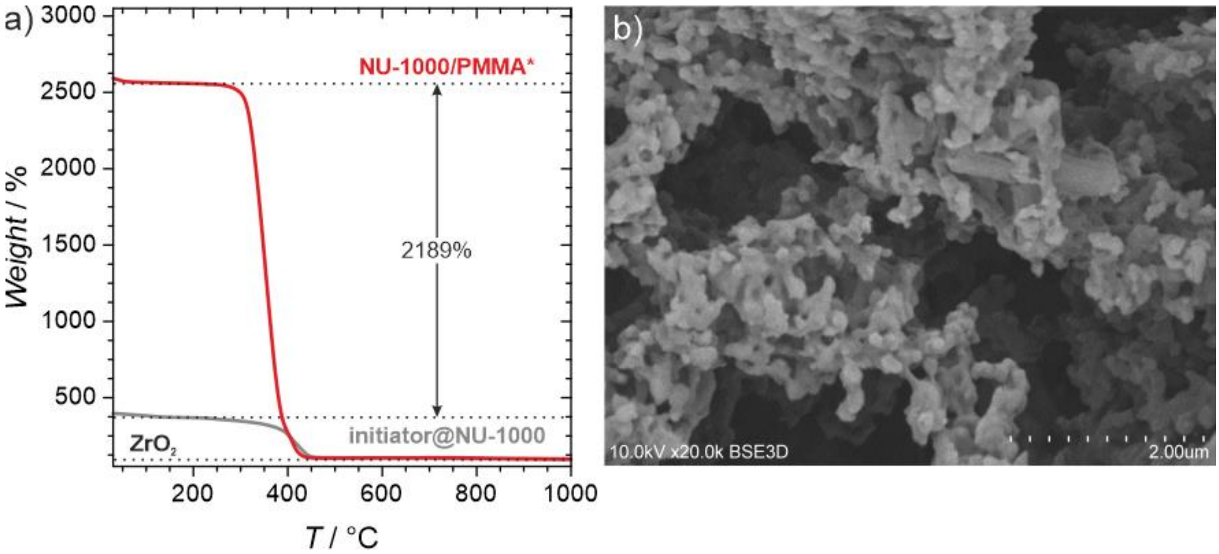

Figure S67. TGA profile (a) and SEM image (b) of **NU-1000/PMMA** before washing with acetone.

S15. Stability of the MOF/polymer/fiber composites

A simple water soaking test was designed to assess the stability of **MOF/polymer/fiber** composites. A 1x0.5 cm piece of the fabric was suspended on 2 mL of water and stirred by hand. Then, the supernatant was removed, and this process was repeated 2 more times. After that, the **MOF/polymer/fiber** samples were dried at 100 °C overnight. The performance of **MOF/polymer/fiber** composites was compared with the sample of **MOF/fiber** composites where instantaneous detachment of MOF was observed (Figure S68b, S69b). The Zr content before and after water treatment was followed using ICP-OES analysis (Tables S7-S8).

Table S7. The Zr content of prepared **NU-1000**-based **MOF/polymer/fiber** determined before and after water treatment.

**Composite sample**	**Zr (wt%)[1]**	
	**as prepared**	**after water treatment**
**NU-1000/PP**	5.9	1.4
**NU-1000/PMMA/PP**	1.8	1.9
**NU-1000/PDMAM/PP**	1.1	0.8
**NU-1000/AC**	2.7	0.8
**NU-1000/PMMA/AC**	0.8	0.8
**NU-1000/PDMAM/AC**	0.4	0.4

[1] based on ICP-OES measurement

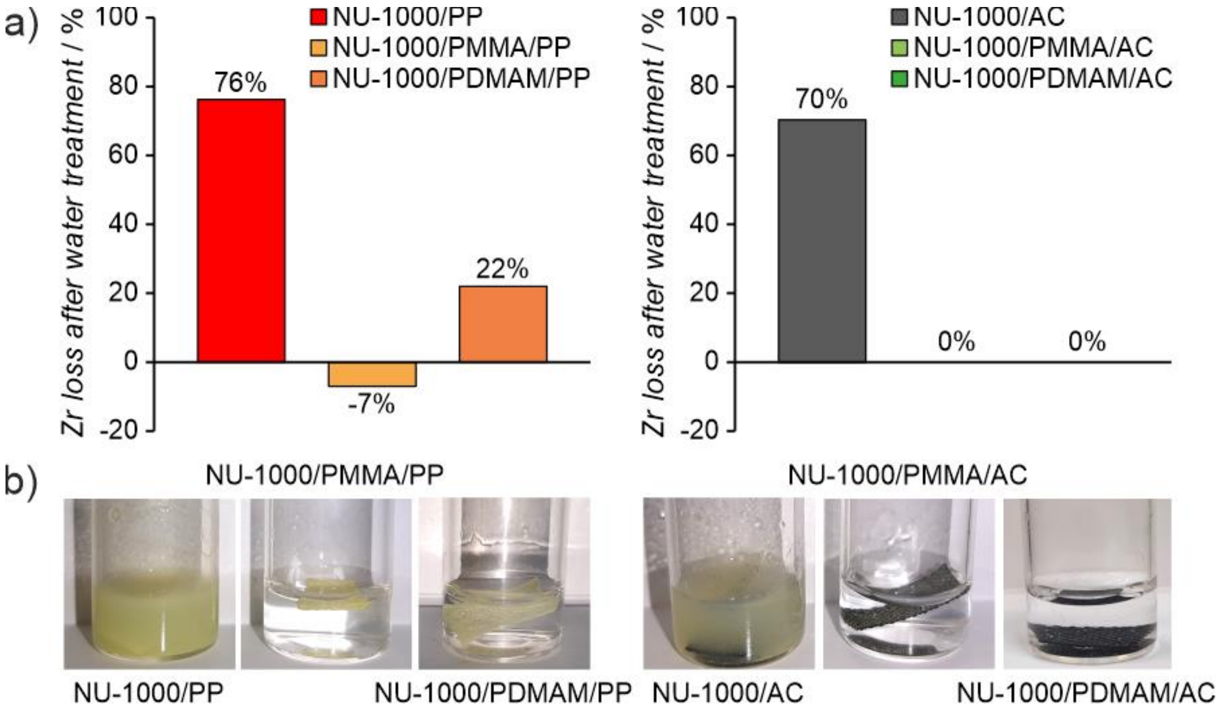

Figure S68. a) Zr loss determined with ICP-OES analysis of **NU-1000/polymer/fiber** composites before and after water treatment (the estimated error of that analysis is 10%). b) Photographs of respective **NU-1000/polymer/fiber** composites immersed in 2 ml of water.

Table S8. The Zr content of prepared **MOF-808**-based **MOF/polymer/fiber** determined before and after water treatment.

Composite sample	Zr (wt%)[1]	
	as prepared	after water treatment
**MOF-808/PP**	8.2	3.1
**MOF-808/PMMA/PP**	7.8	7.0
**MOF-808/PDMAM/PP**	10.0	10.4
**MOF-808/AC**	3.7	0.5
**MOF-808/PMMA/AC**	3.6	2.8
**MOF-808/PDMAM/AC**	5.9	4.7

[1] based on ICP-OES measurement

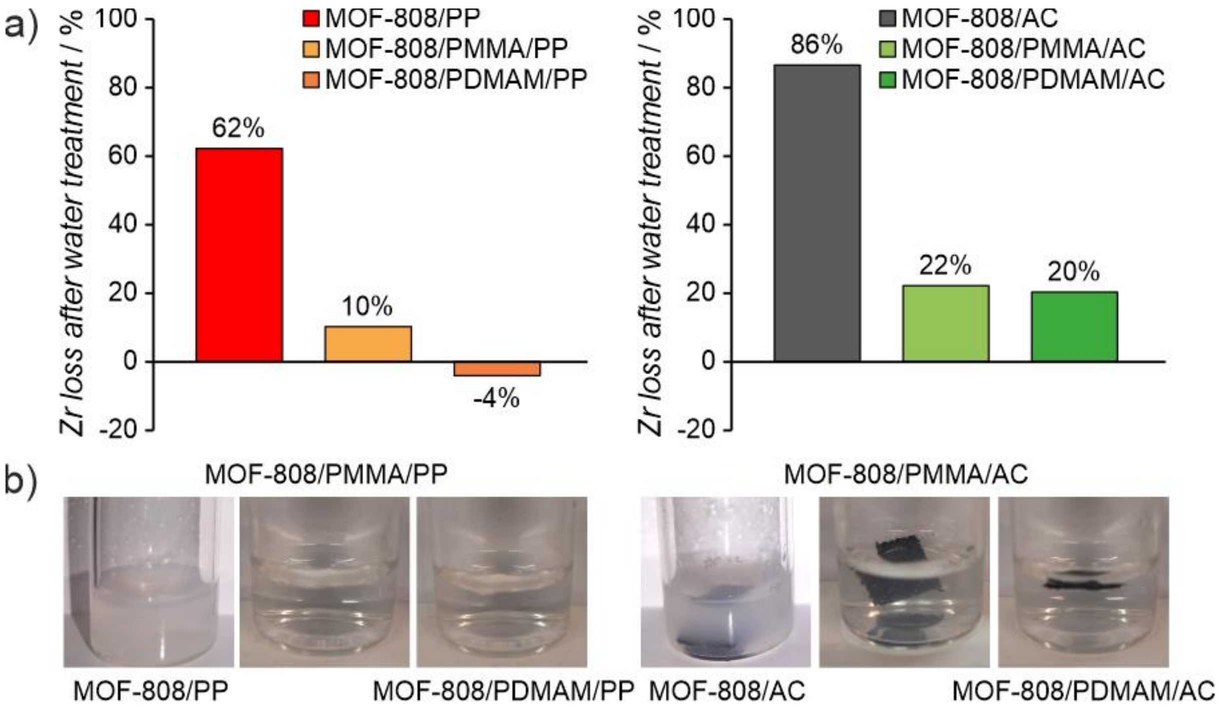

Figure S69. a) Zr loss determined with ICP-OES analysis of **NU-1000/polymer/fiber** composites before and after water treatment (the estimated error of that analysis is 10%). b) Photographs of respective **NU-1000/polymer/fiber** composites immersed in 2 ml of water.

S16. Catalytic studies with diisopropylfluorophosphate (DIFP)

S16.1. Reaction of DIFP with MOF/polymer hybrids

The diisopropylfluorophosphate (DIFP) hydrolysis was tested in the presence of pristine Zr-MOFs or **MOF/polymer** hybrids. Typically, the MOF or **MOF/polymer** sample was placed in the vial with 0.5 ml of water and 1.25 µL of dimethylacetamide (DMA, internal standard). Then, 1.25 µL of DIFP (7.2 µmol) was added and the DIFP conversion in time was monitored using GC analysis. The amount of Zr-MOF or **MOF/polymer** hybrid was fixed in respect to the DIFP:MOF ratio of 2:1. The reactions were carried out at room temperature for 24 hours and the collected data are presented on Figure S70 and Table S9.

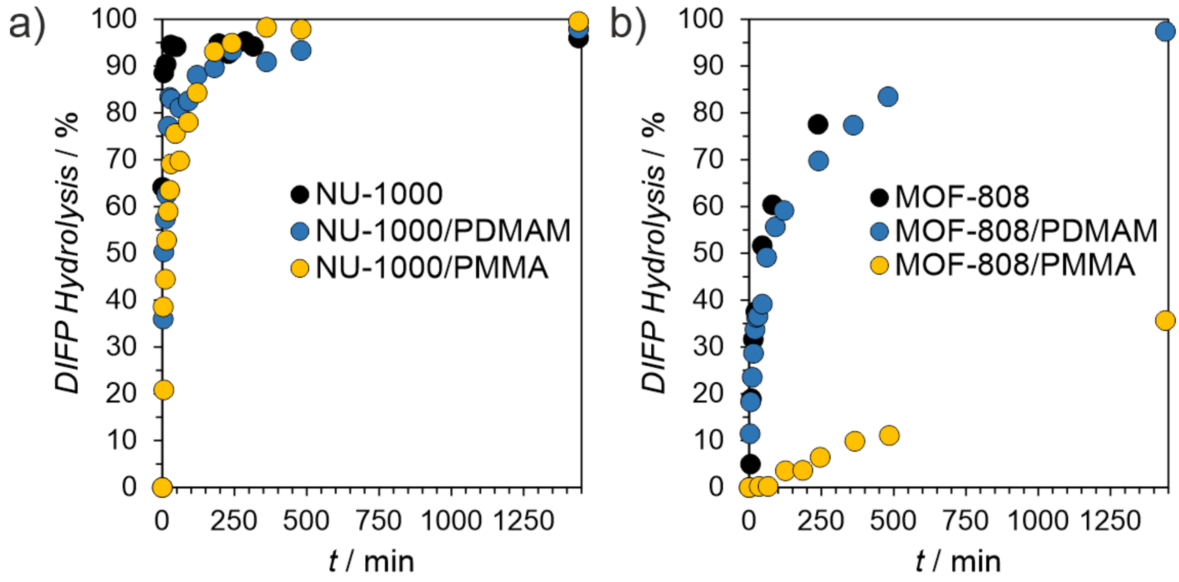

Figure S70. DIFP degradation kinetics for a) **NU-1000/polymer** and b) **MOF-808/polymer** hybrids carried out at room temperature

Table S9. The DIFP hydrolysis after 24 h at room temperature catalyzed by MOF/polymer samples, for comparison the pristine Zr-MOF activity was included.

Zr-MOF or MOF/polymer sample	DIFP hydrolysis (%)	pH of the starting material[2]
**NU-1000**	96	3.8
**NU-1000/PMMA**	100	5.6
**NU-1000/PDMAM**	98	6.9
**MOF-808**	94	2.9
**MOF-808/PMMA**	36	4.8
**MOF-808/PDMAM**	97	7.0

[1] after 24 h, based on GC

[2] MOF/polymer sample was suspended in deionized milliQ water for 2 hours before reading the pH value

S16.2. Reaction of DIFP with MOF/polymer/fiber composites

The reactions of DIFP hydrolysis in the presence of **MOF/polymer/fiber** composites were carried out at 30 °C (for **MOF/polymer/PP** samples) or 60 °C (for **MOF/polymer/AC** samples) for 24 hours. For each reaction, pieces of **MOF/polymer/fiber** with dimensions of 6 cm × 2 cm were cut and weighted in a glass vial. Then, 2 µL of water was added, followed by 0.3 µL of DIFP (1.7 µmol) and the vial was sealed tightly. Depending on the Zr content in the **MOF/polymer/fiber** composites (determined by ICP-OES analysis, Tables S7-S8), the DIFP:MOF molar ratios was approximately 15:1 (for **NU-1000/polymer/fiber**) and 5:1 (for **MOF-808/polymer/fiber**). After 24 hours of incubation at 30 °C or 60 °C, the reagents were extracted with a solution of 2 ml of dichloromethane and 0.3 µL of dimethylacetamide (DMA, internal standard). This extraction was done for 2 h and at the same temperature as the catalysis was carried out. The DIFP conversion after 24 h was determined with GC analysis and the obtained results are presented in Tables S10-S11.

Table S10. The DIFP hydrolysis after 24 h at 30 °C catalyzed by **MOF/polymer/PP** samples.

Zr-MOF or MOF/polymer sample	DIFP hydrolysis (%)
**NU-1000/PMMA/PP**	0
**NU-1000/PDMAM/PP**	66
**MOF-808/PMMA/PP**	5
**MOF-808/PDMAM/PP**	97

[1] after 24 h, based on GC

Table S11. The DIFP hydrolysis after 24 h at 60 °C catalyzed by **MOF/polymer/AC** samples.

Zr-MOF or MOF/polymer sample	DIFP hydrolysis (%)
**NU-1000/PMMA/AC**	0
**NU-1000/PDMAM/AC**	0
**MOF-808/PMMA/AC**	57
**MOF-808/PDMAM/AC**	42

[1] after 24 h, based on GC

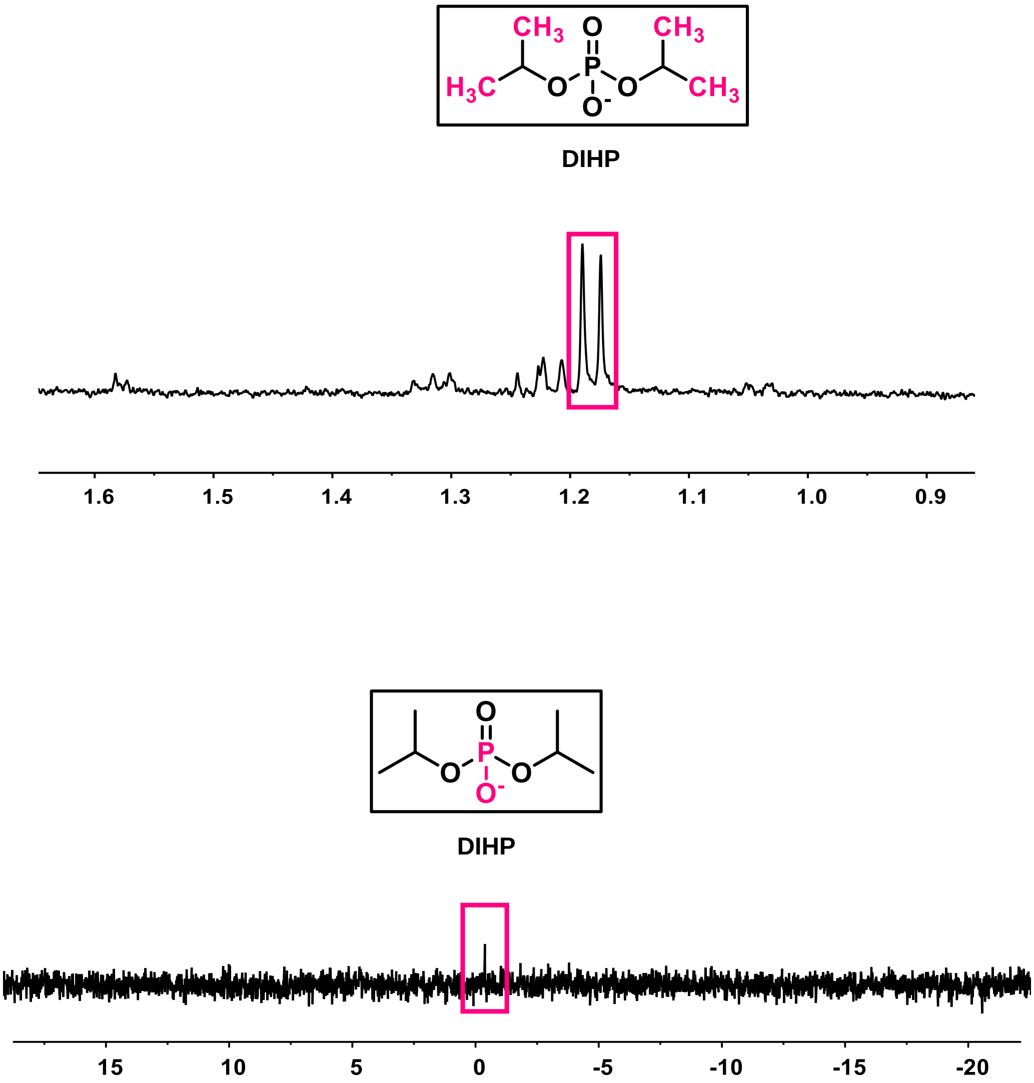

Figure S71. ^{1}H (top) and ^{31}P (bottom) NMR of hydrolysis product (diisopropylphosphate, DIHP) of DIFP degradation by **MOF-808/PDMMA/PP** in water.

S17. MALDI-MS spectra of MOF/polymer hybrids

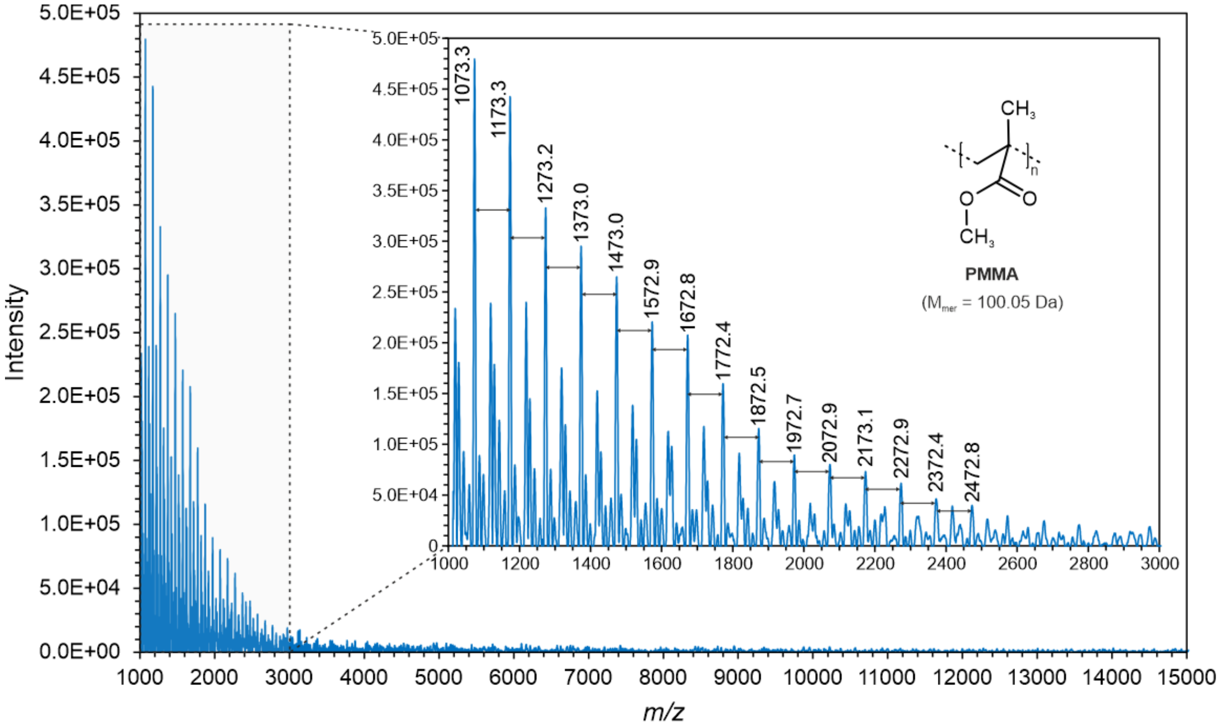

Figure S72. MALDI-MS spectrum of PMMA polymer isolated from decomposed sample of **NU-1000/PMMA.**

S18. References

(1) Mondloch, J. E.; Bury, W.; Fairen-Jimenez, D.; Kwon, S.; Demarco, E. J.; Weston, M. H.; Sarjeant, A. A.; Nguyen, S. T.; Stair, P. C.; Snurr, R. Q.; Farha, O. K.; Hupp, J. T. Vapor-Phase Metalation by Atomic Layer Deposition in a Metal-Organic Framework. *Journal of the American Chemical Society* **2013**, *135* (28), 10294–10297. https://doi.org/10.1021/ja4050828.

(2) Morcombe, C. R.; Zilm, K. W. Chemical Shift Referencing in MAS Solid State NMR. *Journal of Magnetic Resonance* **2003**, *162* (2), 479–486. https://doi.org/10.1016/S1090-7807(03)00082-X.

(3) Rouquerol, J.; Llewellyn, P.; Rouquerol, F. Is the BET Equation Applicable to Microporous Adsorbents? In *Studies in surface science and catalysis*; Elsevier B. V., 2007; Vol. 160, pp 49–56. https://doi.org/10.1016/S0167-2991(07)80008-5.

(4) Walton, K. S.; Snurr, R. Q. Applicability of the BET Method for Determining Surface Areas of Microporous Metal-Organic Frameworks. *Journal of the American Chemical Society* **2007**, *129* (27), 8552–8556. https://doi.org/10.1021/ja071174k.

(5) Islamoglu, T.; Otake, K.; Li, P.; Buru, C. T.; Peters, A. W.; Akpinar, I.; Garibay, S. J.; Farha, O. K. Revisiting the Structural Homogeneity of NU-1000, a Zr-Based Metal–Organic Framework. *CrystEngComm* **2018**, *20* (39), 5913–5918. https://doi.org/10.1039/c8ce00455b.

(6) Dai, S.; Simms, C.; Dovgaliuk, I.; Patriarche, G.; Tissot, A.; Parac-Vogt, T. N.; Serre, C. Monodispersed MOF-808 Nanocrystals Synthesized via a Scalable Room-Temperature Approach for Efficient Heterogeneous Peptide Bond Hydrolysis. *Chem. Mater.* **2021**, *33* (17), 7057–7066. https://doi.org/10.1021/acs.chemmater.1c02174.

(7) Deria, P.; Bury, W.; Hupp, J. T.; Farha, O. K. Versatile Functionalization of the NU-1000 Platform by Solvent-Assisted Ligand Incorporation. *Chem. Commun.* **2014**, *50* (16), 1965. https://doi.org/10.1039/c3cc48562e.

(8) Zhou, Y.; Zhang, Z.; Postma, A.; Moad, G. Kinetics and Mechanism for Thermal and Photochemical Decomposition of 4,4′-Azobis(4-Cyanopentanoic Acid) in Aqueous Media. *Polym. Chem.* **2019**, *10* (24), 3284–3287. https://doi.org/10.1039/C9PY00507B.

(9) Shearer, G. C.; Chavan, S.; Bordiga, S.; Svelle, S.; Olsbye, U.; Lillerud, K. P. Defect Engineering: Tuning the Porosity and Composition of the Metal–Organic Framework UiO-66 via Modulated Synthesis. *Chem. Mater.* **2016**, *28* (11), 3749–3761. https://doi.org/10.1021/acs.chemmater.6b00602.
